# Analysis of EYA3 Phosphorylation by Src Kinase Identifies Residues Involved in Cell Proliferation

**DOI:** 10.3390/ijms20246307

**Published:** 2019-12-13

**Authors:** Aura E. Ionescu, Mihaela Mentel, Cristian V.A. Munteanu, Livia E. Sima, Eliza C. Martin, Georgiana Necula-Petrareanu, Stefan E. Szedlacsek

**Affiliations:** 1 Department of Enzymology, Institute of Biochemistry of the Romanian Academy, Splaiul Independentei 296, 060031 Bucharest, Romania; aura.ionescu@yahoo.com (A.E.I.); mentelmihaela@gmail.com (M.M.); georgianapetrareanu@yahoo.com (G.N.-P.); 2 Department of Bioinformatics and Structural Biochemistry, Institute of Biochemistry of the Romanian Academy, Splaiul Independentei 296, 060031 Bucharest, Romaniaelizamartinc@yahoo.com (E.C.M.); 3 Department of Molecular Cell Biology, Institute of Biochemistry of the Romanian Academy, Splaiul Independentei 296, 060031 Bucharest, Romania; lsima@biochim.ro

**Keywords:** eyes absent 3, Src kinase, mass spectrometry, protein phosphorylation, cell proliferation

## Abstract

Eyes absent (EYA) are non-thiol-based protein tyrosine phosphatases (PTPs) that also have transcriptional co-activator functions. Their PTP activity is involved in various pathologies. Recently, we demonstrated that Src tyrosine kinase phosphorylates human EYA3 by controlling its subcellular localization. We also found EYA3′s ability to autodephosphorylate, while raising the question if the two opposing processes could be involved in maintaining a physiologically adequate level of phosphorylation. Using native and bottom-up mass spectrometry, we performed detailed mapping and characterization of human EYA3 Src-phosphorylation sites. Thirteen tyrosine residues with different phosphorylation and autodephosphorylation kinetics were detected. Among these, Y77, 96, 237, and 508 displayed an increased resistance to autodephosphorylation. Y77 and Y96 were found to have the highest impact on the overall EYA3 phosphorylation. Using cell cycle analysis, we showed that Y77, Y96, and Y237 are involved in HEK293T proliferation. Mutation of the three tyrosine residues abolished the pro-proliferative effect of EYA3 overexpression. We have also identified a Src-induced phosphorylation pattern of EYA3 in these cells. These findings suggest that EYA3′s tyrosine phosphorylation sites are non-equivalent with their phosphorylation levels being under the control of Src-kinase activity and of EYA3′s autodephosphorylation.

## 1. Introduction

EYA proteins belong to a family of evolutionarily conserved transcription factors and cofactors, referred to as the Pax-Six-Eya-Dach Network (PSEDN). This network has important roles in the development and homeostasis of various tissues and organs such as eye, kidney, nervous system, ear, and muscle [1,2] as well as in limb formation [3,4], gonadogenesis [5], and neurogenesis [6,7]. Loss-of-function mutations in the eyes absent genes (*EYA*) can lead to several congenital syndromes (cardiofacial syndrome [8], bronchio-oto-renal syndrome [9], oto-facio-cervical syndrome [10], congenital cataract [11], and late onset deafness [12]). On the other hand, overexpression of *EYAs* has been detected in various types of cancers such as colorectal [13], breast [14,15], and epithelial ovarian cancer [16], Wilms’ tumor [17], lung and esophageal adenocarcinoma [18,19], and malignant peripheral nerve sheath tumors [20].

EYA proteins contain specific domains responsible for transactivation [21] and protein tyrosine phosphatase [22,23] activities. The EYA transcriptional co-activator function resides in the N-terminal domain (NTD), which is a region poorly conserved among vertebrates [1] and absent in plants [24]. The protein tyrosine phosphatase (PTP) activity is localized in the C-terminal domain and contains characteristic motifs of the haloacid dehalogenase (HAD) superfamily, which makes EYA a member of the phosphatase subgroup of HAD [2,22,23]. In addition to its own tyrosine phosphatase activity, EYA has threonine phosphatase activity but only when interacting with the protein phosphatase 2A (PP2A)-B55α holoenzyme. This interaction proved to play a critical role in c-Myc stabilization and late stage metastasis in the breast cancer model [25]. There are four human homologous EYA proteins (EYA 1 to 4), which all contain a highly conserved PTP catalytic domain, termed the Eya Domain (ED) and a variable N-terminal region. EYA homologues have been shown to be involved in various diseases. For example, EYA1′s PTP activity has been implicated in breast cancer tumor growth as well as in cellular proliferation through cyclin D1 transcriptional induction [26]. Similarly, it has been reported that the PTP activity of EYA 1, 2, and 3 is required for transformation, migration, invasion, and metastasis in MCF-7 and MDA-MB-231 breast cancer cell lines [14]. Despite the large number of reports implicating EYA proteins in pathological conditions, limited information is available regarding their substrates. So far, three physiological substrates for EYA’s PTP activity have been identified: histone H2A.X (phosphotyrosine-pY-142) [27,28], estrogen receptor β (pY36) [29], which both have nuclear localization, and WD repeat-containing protein 1 (WDR1), which is a cytoskeletal protein [30].

Tyrosine phosphorylation, which is one of the most important post-translational modifications, regulates diverse cellular processes such as growth, proliferation, differentiation, migration, organelle trafficking, and apoptosis [31,32,33]. Dysregulation of tyrosine kinase signaling pathways is one of the leading causes of cancer progression [34]. For example, c-Src activation has been reported to generate more than 50% of tumors in liver, colon, breast, lung, and pancreas [35]. Recently, we have demonstrated that c-Src phosphorylates tyrosine residues of human EYA1 and EYA3 to control their nuclear and cytoskeletal localization [30]. We have also found that EYA1 and EYA3 are capable of autodephosphorylation [30]. These data indicate a potential implication of EYA tyrosine phosphorylation and autodephosphorylation in regulating physiological processes and contributing to pathological conditions. Thus, EYA proteins have built-in self-regulating capabilities that control their own function. Information on specific phosphorylated residues and the extent to which they are modified is still unknown. Due to the simultaneous action of tyrosine phosphorylation and autodephosphorylation, it is challenging to perform such mapping studies. In this article, we used a combination of native mass spectrometry (MS) [36,37] and bottom-up mass spectrometry [38,39,40] to reveal tyrosine phosphorylation and dephosphorylation sites of human EYA3. High resolution native MS enabled us to evaluate the stoichiometry of phosphorylation at the level of the intact protein, whereas bottom-up mass spectrometry allowed us to determine the specific sites of phosphorylation. We show that in vitro Src selectively phosphorylates 13 tyrosine sites in EYA3. Most of them are located within the N-terminal region. Then, we evaluated the contribution of the identified phosphotyrosine residues to overall EYA3 phosphorylation. To determine the biological relevance of the EYA3 phosphorylation/dephosphorylation-cycle, we investigated the proliferation of HEK293T cells overexpressing wild-type EYA3 (EYA3 WT) or an EYA3 mutant, containing tyrosine to phenylalanine (Y → F) mutations of three residues, which we identified as phosphorylation sites (Y77, Y96, and Y237). Expression of this mutant decreased the proliferation rate of HEK293T cells, which reveals a potential role for the phosphorylated sites in cell proliferation. Cell cycle analysis revealed that these residues play a role in the modulation of the cell cycle distribution. Using nano-high performance liquid chromatography with tandem mass spectrometry (nLC-MS/MS) for mapping tyrosine phosphorylation sites, we have identified a novel phosphorylation pattern of EYA3 in HEK293T cells. Overall, our results reveal the complex nature of the balance between EYA3 phosphorylation and autodephosphorylation and are expected to lead to a better understanding of EYA’s role in pathogenesis.

## 2. Results

### 2.1. Dynamics of Src Kinase-Mediated Phosphorylation of EYA3 and Its Autodephosphorylation, Studied by Native Mass Spectrometry

The interaction between two EYA3 molecules is mediated by the ED and depends on Src-induced phosphorylation [30]. However, it is still unclear how many tyrosine residues of EYA3 are phosphorylated by Src and, subsequently, autodephosphorylated. In addition, the kinetics of these processes has not yet been elucidated. To address these questions, we used native MS to monitor the phosphorylation stoichiometry on the intact EYA3 protein, purified after prokaryotic expression in *E. coli*.

**Figure 1:**
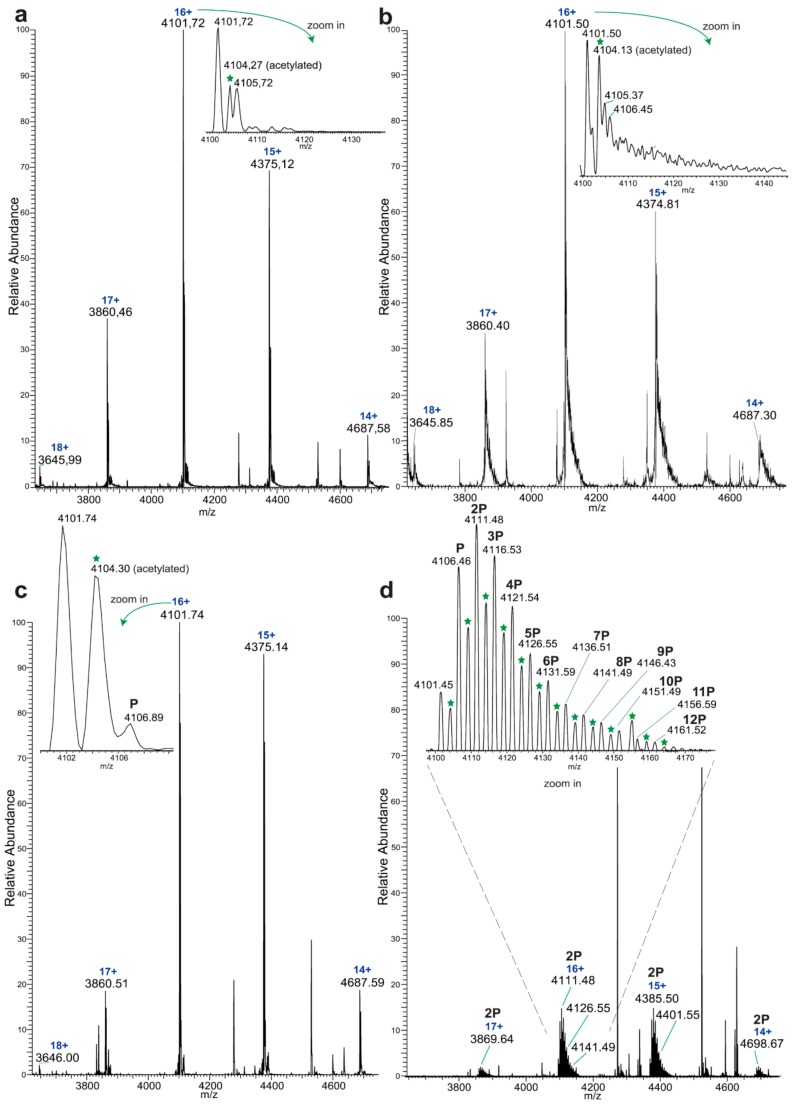
Native mass spectrometry (MS) analysis of N-terminal His-tagged wild-type EYA3 (EYA3 WT) and D311N proteins before and after Src phosphorylation. (**a**), (**b**) Native mass spectrum of purified 6xHis-EYA3 WT (**a**) and 6xHis-EYA3 D311N (**b**). The molecular weights were accurately calculated (using the m/z values of all identified charge states for that protein: 18+, 17+, 16+, 15+, and 14+) and the obtained values correspond to the protein sequences lacking the start Methionine: 65611.22 ± 0.9 Da for 6xHis-EYA3 WT and 65608.09 ± 1.1 Da for 6xHis-EYA3 D311N. The theoretical molecular weights of the purified proteins are 65742.1 Da (6xHis-EYA3 WT) and 65741.1 (6xHis-EYA3 D311N). In the zoomed in images from (**a**) and (**b**), two major peaks were observed, corresponding to two proteoforms of EYA3: (**a**) For charge 16+ of EYA3 WT, there are two m/z values including one with the higher intensity and m/z value of 4101.72, corresponding to EYA3 with the first Methionine cleaved and another one, which is at a m/z difference of 2.55 (m/z = 4104.27), harboring a modification of 41.23 ± 0.8 Da, corresponding to acetylation (green star above the corresponding peak). (**b**) For charge 16+ of 6xHis-EYA3 D311N, the m/z difference is 2.63 (4104.13–4101.50), which results in a modification of 42.73 ± 1.2 Da. This also corresponds to acetylation. The calculated molecular weights for the acetylated proteins are 65652.45 ± 0.3 Da for acetylated 6xHis-EYA3WT and 65650.82 ± 2 Da for the acetylated mutant protein. (**c**) Native mass spectrum of 6xHis-EYA3 WT after in vitro tyrosine phosphorylation with v-Src. Adjoining peaks of the charge state envelope of 6xHis-EYA3 WT after 2 h of incubation with v-Src kinase and ATP. Zoom in on charge 16+: unphosphorylated molecules (4101.74 and acetylated, at 4104.30) and molecules with only one phosphorylated residue (**P**, m/z = 4106.89) were detected. (**d**) Native mass spectrum of 6xHis-EYA3 D311N after 2 h in vitro tyrosine phosphorylation with v-Src (ATP). Adjoining peaks of the charge state envelope reveal the presence of modified EYA3 molecules. For each of the four charge states of 6xHis-EYA3 D311N (17+, 16+, 15+, 14+), the highest intensity is represented by molecules carrying two phosphate moieties (**2P**). Zoom in on charge 16+: molecules with up to 12 phosphorylated residues (**P** to **12P**) were detected. The acetylated molecules have the same maximum number of phosphorylated residues.

Native MS of EYA3 showed a major charge distribution corresponding to a molecular weight of 65,611.22 ± 0.9 Da (mass/charge, noted m/z, of 4101.72 at charge 16+, Figure 1a, Figure S1a,b). Due to the high resolution of the Orbitrap mass spectrometer, two proteoforms of EYA3 were identified with one corresponding to EYA3 that lacks the initial methionine residue (charge 16+: m/z = 4101.72 in Zoom in Figure 1a, charges 15+, 14+, and 17+ in Figure S1c–e, and m/z = 4101.50 in zoom in Figure 1b), and another in which Ser2 is acetylated (charge 16+: m/z = 4104.27 in Zoom in Figure 1a and m/z = 4104.13 in zoom in Figure 1b, peptide SHHHHHHSMDIEENLY, S1-Acetyl, identified by nLC-MS/MS-Figure S2). EYA3 was incubated with Src kinase and tyrosine phosphorylation tracked by native MS (Figure 1c,d, Figures S3 and S4). Incubation of EYA3 WT with Src and ATP for 2 h generated molecules with only one phosphorylated residue (Figure 1c, Figure S3). However, molecules with up to three phosphorylated residues were observed after only 5 min when using a phosphatase-deficient mutant of EYA3 (EYA3 D311N) [23,27,30] (Figure S5). After 2 h, up to 12 phosphorylation sites were observed on EYA3 D311N (Figure 1b versus Figure 1d, when compared to Figure S5, the intensities of the signals corresponding to the unphosphorylated molecules decreased and those of the phosphorylated molecules increased) highlighting the importance of the ED in controlling the phosphorylation status of EYA3.

**Figure 2:**
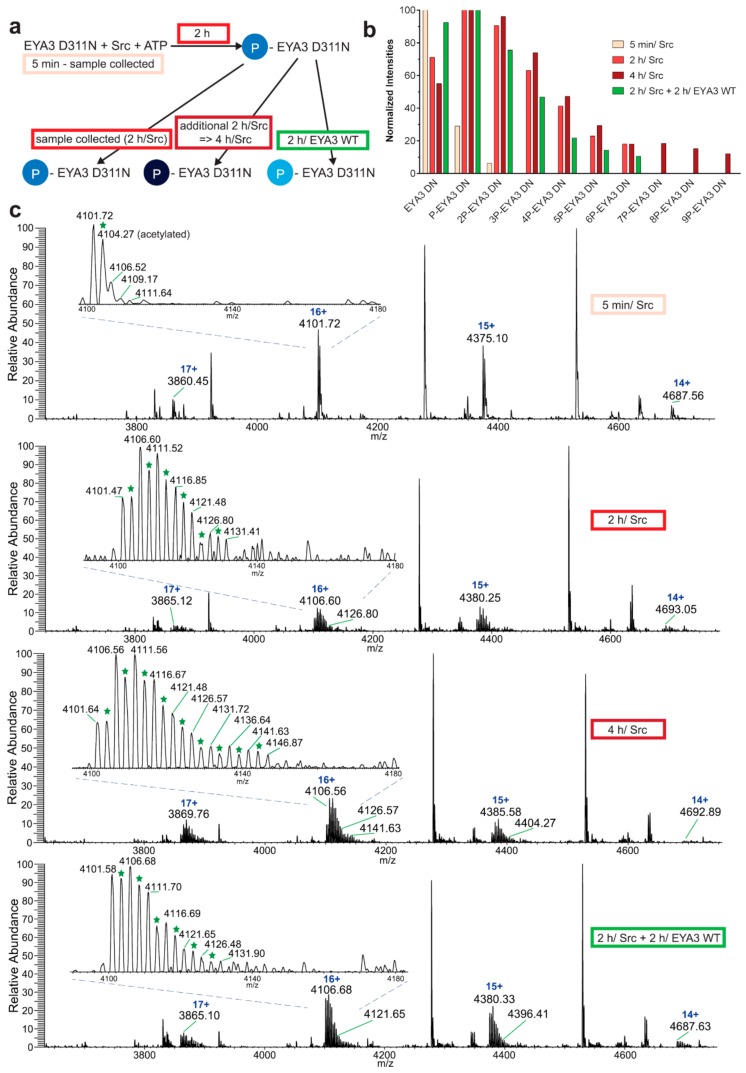

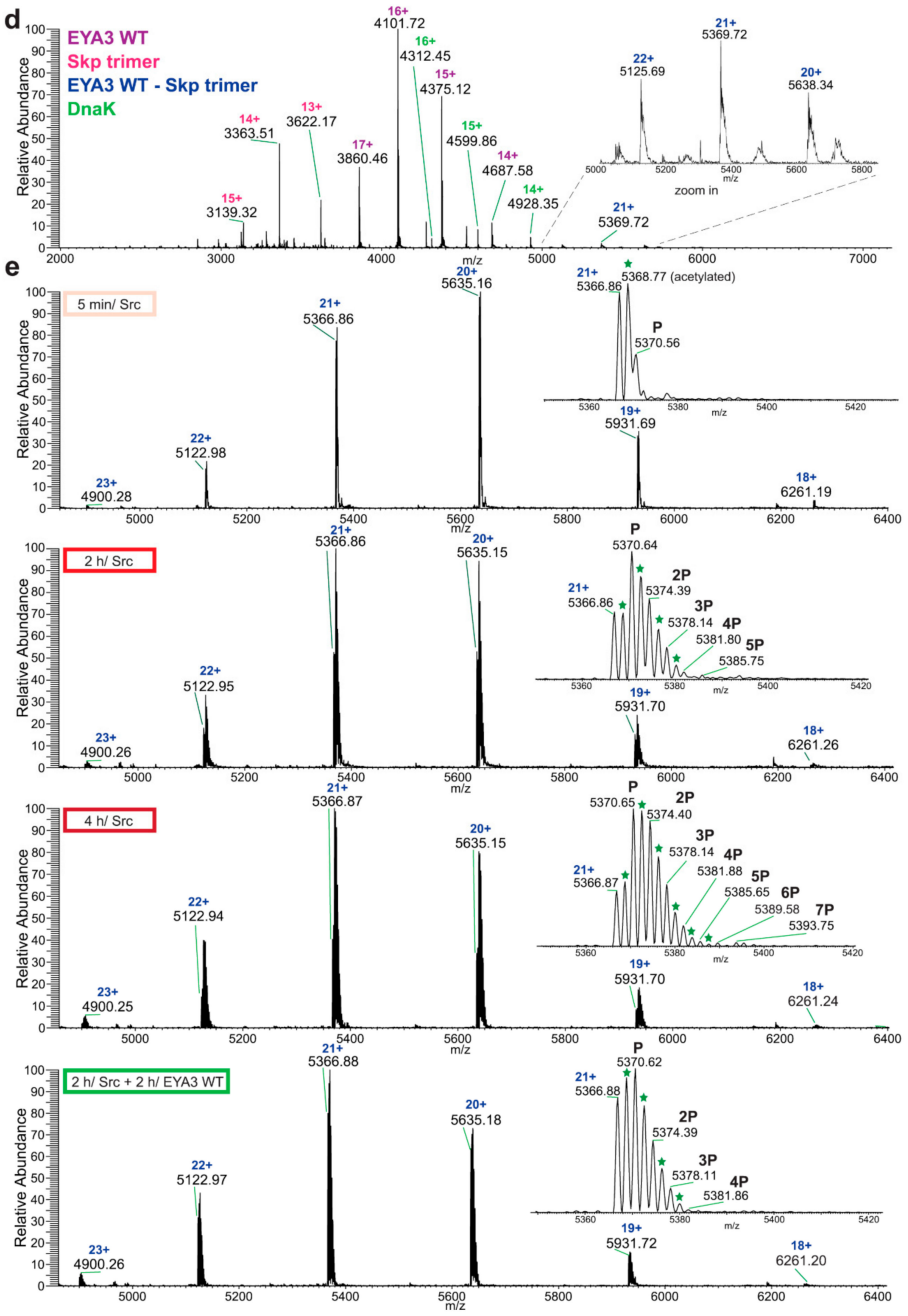
Dynamics of EYA3 tyrosine phosphorylation by Src kinase and its autodephosphorylation, studied by native mass spectrometry. (**a**) Workflow for the dynamics of EYA3 tyrosine phosphorylation and autodephosphorylation analyzed by native MS. (**b**) Quantification of the raw data of 6xHis-EYA3 D311N molecules, which carry charge 16+. (**c**) Native MS raw data of the 6xHis-EYA3 D311N molecules (peaks of the charge state envelope and zoom in on molecules with charge 16+) from each of the four samples. (**d**) Native MS spectrum of purified EYA3. Electrospray ionization-mass spectrometry (ESI-MS) of 6xHis-EYA3 WT at pH 6.8: EYA3 is present with molecular weight (MW) accuracy of +/− 1 Da (loss of methionine, calculated molecular weight of 65611.57 +/- 0.5 Da, charges shown in purple). The calculated molecular weight of 47075.05 ± 0.2 Da (revealed from the charges shown in pink), accurately reveals the existence of a Skp homo-trimer in which the monomer lacks the first 20 amino acids. The molecular weight of 68983 ± 0.2 Da (calculated from the charges shown in green) corresponds to the full-length chaperone protein DnaK lacking the N-terminal methionine, which is present in small amounts. Zoomed in image: charge state envelope of EYA3-Skp complex (charges shown in blue). The molecular weight is calculated below in which case the instrument is especially set up to have a higher accuracy and resolution at higher m/z values. EYA3-Skp was the only complex detected in the spectrum. Purified 6xHis-EYA3 D311N has a similar native MS spectrum at a pH of 6.8 (Skp and DnaK also present). (**e**) Native MS raw data of 6xHis-EYA3 D311N-Skp trimer complex (peaks of the charge state envelope, zoom in is on molecules with charge 21+) from each of the four samples of the experiment on EYA3 phosphorylation dynamics (workflow described above at point (**a**)). The molecular weight of the complex, which is calculated from the charge state envelope (annotated peaks), is 112683.3 ± 0.1 Da (112683.3 Da ≈ 65608.09 Da + 47075.05 Da). In these spectra, for a certain charge state, often the highest peak corresponds to the complex between the Skp trimer and the acetylated EYA3 D311N proteoforms or the complex in which EYA3 D311N is phosphorylated at a single residue (see zoomed in images).

Next, we investigated the dynamics of purified EYA3 tyrosine phosphorylation and autodephosphorylation. Briefly, D311N was initially in vitro phosphorylated by Src kinase. Then the reaction was either stopped or continued for an additional time (2 h) in the presence or absence of WT EYA3 (Figure 2a–c). After 5 min, unphosphorylated EYA3 molecules displayed the highest intensity. Mono-phosphorylated and di-phosphorylated EYA3 molecules were also present, but their intensities were much smaller. After 2 h, the intensity of the unphosphorylated form considerably decreased. The monophosphorylated molecules were the most abundant, but intensities decreased stepwise with the increase in the number of phosphate moieties added to EYA3 (from 2 to 6). Molecules with up to six phosphorylated sites were detected. Even after 4 h of Src incubation, the monophosphorylated form is still the most abundant one and molecules with six, seven, and eight phosphorylated residues have comparable intensities, with a total of nine phosphosites detected. Notably, the phosphorylation signal of molecules with high numbers of phosphate moieties decreased (for 2P-D311N to 6P-D311N molecules) or even disappeared (the 7P-D311N, 8P-D311N, and 9P-D311N molecules) after the 2-h incubation of D311N with WT. The most significant decrease could be observed for molecules with four and five phosphorylated sites. Conversely, the highest signal was detected on molecules having one phosphorylated residue and, remarkably, the intensity of the unphosphorylated molecules increased significantly. Notably, the acetylated proteoform of EYA3 displayed identical characteristics, which suggests that this modification does not affect the overall kinetics of EYA3 tyrosine phosphorylation (Figure S6).

Upon more detailed analysis, native MS (confirmed by bottom-up MS, see Figure S7) of the EYA3 proteins revealed additional peaks corresponding to EYA3 in the complex with the chaperone trimer Skp [38] (Figure 2d, Figures S8 and S9) from *E. coli*. DnaK [39,40], another chaperone protein of *E. coli* was also present, but to a relatively minor extent (Figure 2d and Figure S7) [41]. This suggests that EYA3 was likely in equilibrium between its unbound and chaperone-bound form in our experiments. Thus, we additionally analyzed the phosphorylation of the EYA3 D311N-Skp complex. Interestingly, the EYA3 D311N-Skp complex was also phosphorylated—molecules with up to seven phosphorylated residues were observed, instead of nine (Figure 2e) suggesting that Skp partly hinders tyrosine phosphorylation by Src kinase.

These experiments reveal the complex interplay between Src-induced phosphorylation and EYA3 autodephosphorylation, with potential implications in cellular physiology and pathology.

### 2.2. Detection of Src-Phosphorylated Tyrosine Residues in EYA3 and Identification of EYA3 Autodephosphorylation Sites

**Figure 3:**
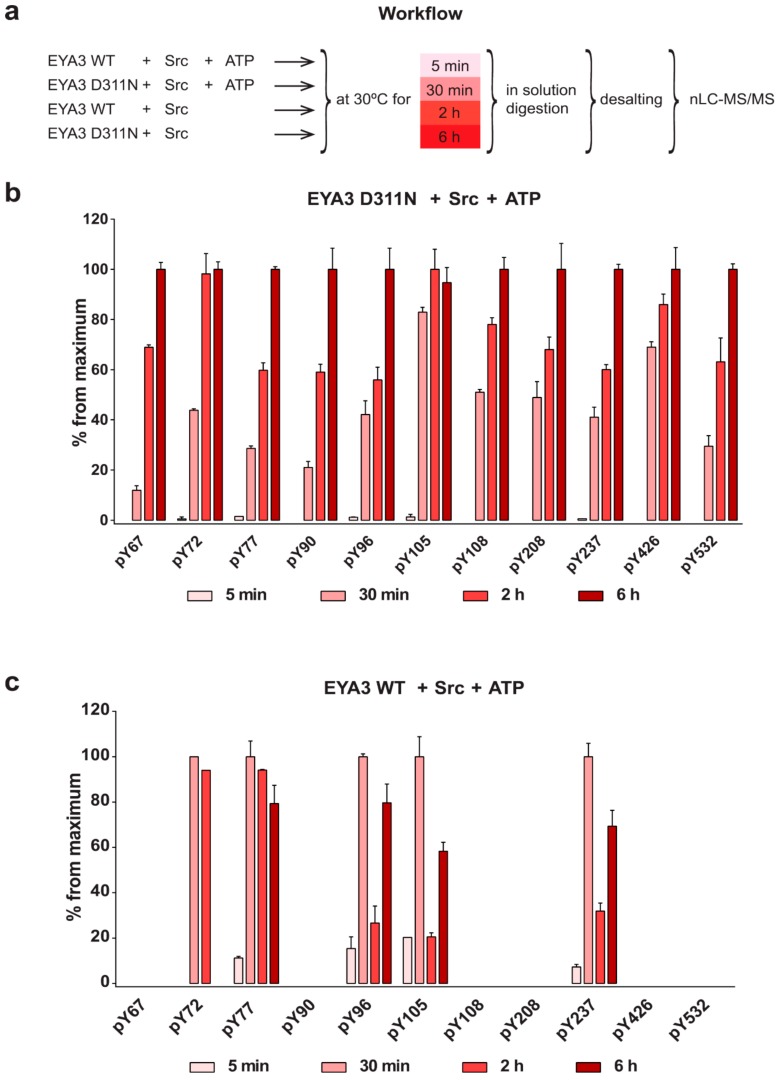
Src-phosphorylated tyrosine residues of EYA3 and autodephosphorylation residues. (**a**) Workflow of the experiment. (**b**) and (**c**) Results obtained from nano-high performance liquid chromatography with tandem mass spectrometry (nLC-MS/MS) analysis followed by data interpretation and quantification: graphical representation of the evolution of phosphorylation for tyrosine residues detected as phosphorylated in the in vitro reactions of EYA3 D311N in the presence of Src and ATP (EYA3 D311N + Src + ATP) (**b**) and EYA3 WT in the presence of Src and ATP (EYA3 WT + Src +ATP) (**c**).

Src-dependent tyrosine phosphorylation and EYA3 autodephosphorylation are opposing processes, which may regulate the cellular functions of EYA3. Initially, we investigated tyrosine phosphorylation in the presence of Src kinase using the catalytically inactive EYA3 D311N mutant (which, consequently, is not capable of autodephosphorylation). The process of autodephosphorylation was studied using EYA3 WT. Incubation of the enzymatically active form of EYA3 with Src kinase allowed monitoring of tyrosine phosphorylation kinetics. The experimental technique used in this purpose was nLC-MS/MS with higher-energy collisional dissociation (HCD) as a fragmentation method. Thus, His-tagged EYA3 WT and EYA3 D311N were incubated with ATP and GST-v-Src, at 30 °C. The reactions were quenched at different time points (5 min, 30 min, 2 h, and 6 h) by the addition of ethylenediaminetetraacetic acid (EDTA) (Figure 3a).

Time-course analyses of D311N phosphorylation with Src revealed an increase in the number of phosphorylated tyrosine residues, from five after 5 min of incubation (Y72, Y77, Y96, Y105, Y237) to 11 after 6 h (Y67, Y72, Y77, Y90, Y96, Y105, Y108, Y208, Y237, Y426, Y532) (Figure 3b, Figure S10a–k). Although all detected tyrosine residues have been phosphorylated within 30 min of incubation with Src, it could be observed that the kinetics of tyrosine phosphorylation is not the same for different residues.

In the case of EYA3 WT phosphorylation with Src, we could detect four tyrosine residues phosphorylated after 5 min (Y77, Y96, Y105, and Y237) and the same sites remained phosphorylated even after 6 h of incubation with the kinase (Figure 3c). The tyrosine residue at position 72 was phosphorylated at 30 min and even after 2 h, but eventually (after 6 h) was fully dephosphorylated. Therefore, the seven tyrosine residues Y67, Y72, Y90, Y108, Y208, Y426, and Y532 not detected in this case but detected in the time course EYA3 D311N Src-phosphorylation, could be considered the main autodephosphorylation sites. Within the first 5 min, phosphorylation was minimal (approximatively 20% from the maximum) for residues Y77, Y96, Y105, and Y237. For the next 25 min, tyrosine autodephosphorylation was practically absent while phosphorylation was intense and reached its peak for all phospho-tyrosine sites. Between 30 min and 2 h, a decrease in phosphorylation for Y96, Y105, and Y237 could be observed (from 100% to around 30%), which suggests that, during this time interval, autodephosphorylation started to play a significant role. Interestingly, the fall was not that steep for Y77 and Y72. On the other hand, the relatively less substantial increase of phosphorylation (Y96, Y105, and Y237) or even its decrease (Y77) between 2 and 6 h suggest that Src-induced phosphorylation and EYA3 autodephosphorylation compensated each other. Comparing the time evolution of EYA3 WT phosphorylation with the one of the inactive EYA3 mutant (Figure 3b,c), we infer that autodephosphorylation of phospho-Y105 is most intense at the 2-h time point. The same comparison reveals that autodephosphorylation of phospho-Y96 and Y237 is not as dramatic as for phospho-Y105. Remarkably, Y77 phosphorylation in the inactive form of EYA3 doubles between 30 min and 2 h. On the other hand, in the same time interval, phosphorylation of Y77 in the catalytically active form of EYA3 remains mainly unchanged. Hence, this finding suggests a consistent autodephosphorylation of phospho-Y77 in the active form of EYA3. The same explanation can be applied to phospho-Y72. However, between 2 and 6 h, phospho-Y72 and Y77 behave differently. Thus, based on a similar way of reasoning, we can infer that, while the autodephosphorylating activity predominates for Y72, for Y77, Src-phosphorylation and autodephosphorylation counterbalance each other (Figure 3b versus Figure 3c).

Due to these results, we wondered if Src-phosphorylation of EYA3 WT might have an impact on its protein tyrosine phosphatase activity, which provides autodephosphorylation capacity. Thus, we performed an experiment in which, initially, 6xHis EYA3 WT was phosphorylated or not with Src kinases, for 2 h. Then we assessed the enzymatic activity of EYA3 WT for 1 h, using para-nitrophenylphosphate (pNPP) as a substrate. Results show that Src-phosphorylated EYA3 WT has slightly increased PTP activity (Figure S11). Based on these results, we can re-analyze the phosphorylation trend of Y72, Y77, Y96, Y105, and Y237 from EYA3 WT incubated with v-Src (Figure 3c). We can assume that, in the first 30 min, EYA3 is intensely phosphorylated. Next, tyrosine phosphorylated EYA3 WT, which has higher PTP activity, manages to dephosphorylate most of the phospho-Y96, Y105, and Y237 residues and, to a lesser extent, phospho-Y72 and Y77. Then, the initial process is somehow repeated, which develops toward a steady state.

The four tyrosine residues on EYA3 WT that remain phosphorylated over time (Y77, Y96, Y105, and Y237) could be considered tyrosine residues with increased resistance to autodephosphorylation. Analyzing the phosphorylation trend of Y72, Y77, Y96, Y105, and Y237 from EYA3 WT incubated with v-Src, we can assume that 30 min is the time point at which phosphorylation reaches its peak while 2 h is the moment when the dephosphorylation is most intense (Figure 3c).

### 2.3. Dynamics of In Vitro EYA3 D311N Tyrosine Phosphorylation and Dephosphorylation

Our native MS data show that wild-type EYA3 is capable of dephosphorylating the Src-phosphorylated form of the phosphatase-deficient EYA3 D311N. The bottom-up approach enabled us to characterize the phosphorylation kinetics of each of the phosphotyrosine residues identified, both for wild-type and phosphatase-deficient mutant EYA3 proteins. Based on the evidence gathered, our next goal was to identify the order in which the phosphorylated residues detected on EYA3 D311N are dephosphorylated by active PTP. The difference between the experimental approach in this case is that the kinase is inactivated, so that EYA3 D311N phosphorylation would not interfere with its dephosphorylation. 

**Figure 4:**
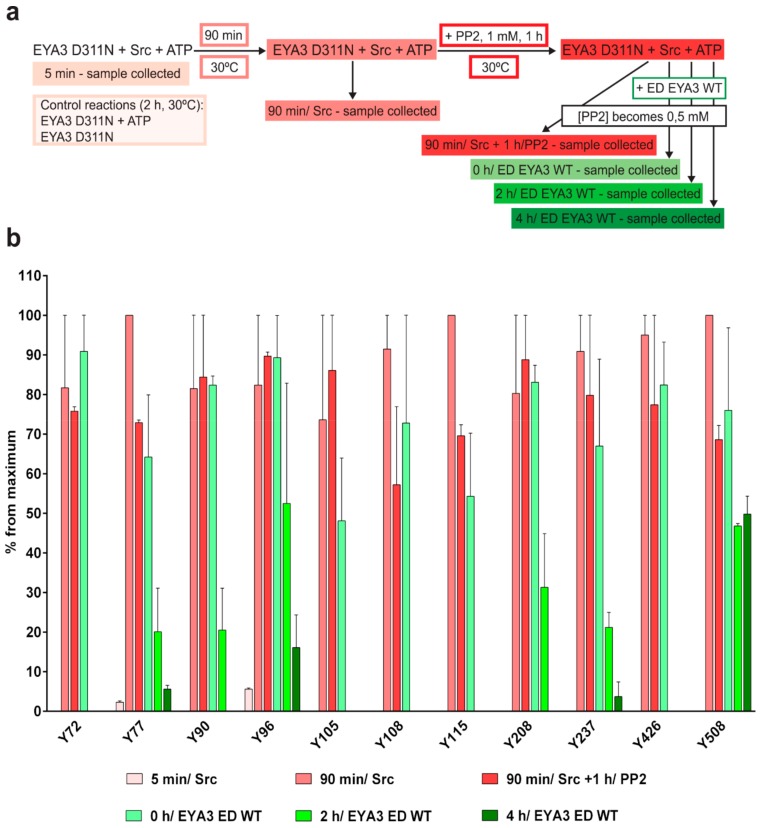
The dynamics of in vitro EYA3 D311N tyrosine phosphorylation (by v-Src) and dephosphorylation (by ED EYA3 WT), analyzed by nLC-MS/MS. (**a**) Workflow of the experiment, which was further analyzed by nLC-MS/MS, in order to establish dynamics of in vitro EYA3 D311N tyrosine phosphorylation and dephosphorylation. (**b**) Site-specific relative quantification of the changes in the phosphorylation status of the tyrosine residues detected on EYA3 D311N. Characterization of the phosphorylation evolution for a certain tyrosine residue was made by relative quantification. The bar chart shows the mean ± SEM corresponding to two independent experiments, each having two technical replicates (*n* = 4).

Initially, we performed in vitro phosphorylation of D311N by v-Src N-terminally fused to GST (GST-v-Src). Next, we inactivated the kinase using PP2 [42], a Src-family selective tyrosine kinase inhibitor. Dephosphorylation of the tyrosine phosphorylated inactive mutant was eventually accomplished using a catalytically active form of EYA3, ED EYA3 WT (workflow presented in Figure 4a). After Western blot confirmation of phosphorylation and dephosphorylation (Figure S12), the D311N bands were in-gel digested with sequencing grade chymotrypsin. Extracted peptides were analyzed by nLC-MS/MS using collision-induced dissociation (CID) and electron-transfer dissociation (ETD) fragmentation methods. The variation in phosphorylation for each of the detected phosphotyrosine residues was evaluated by relative quantification. ED EYA3 WT was chosen instead of full length EYA3 WT because EYA3 WT would otherwise co-migrate with EYA3 D311N and prevents us from distinguishing peptides originating from EYA3 D311N.

Following this approach, 11 tyrosine residues were identified as being phosphorylated, including Y115 and Y508 (Figure S13), which are new ones. Phospho-Y67 and Y532 were not detected in this case. Y105 was intensively dephosphorylated in EYA3 WT when it was incubated with Src and ATP (Figure 3c), and was found dephosphorylated here after 2 h of incubation with the active phosphatase. Only six residues (Y77, Y90, Y96, Y208, Y237, and Y508) were still phosphorylated after 2 h of incubation with ED EYA3 WT while, after 4 h of incubation, only four of the 11 phosphotyrosine residues were still found phosphorylated. Unlike phospho-Y77, Y96, and Y237, which have similar dephosphorylation kinetics (0 to 4 h/ ED EYA3 WT), Y508 has the same level of phosphorylation after 2 and 4 h of incubation with ED EYA3 WT. These results suggest that phospho-Y508 is also a residue with high resistance to autodephosphorylation, and the only residue from the C-terminal part of EYA3, which displays resistance to this process.

Based on all our data obtained from the nLC-MS/MS experiments (Figure 3b,c, and Figure 4), it can be concluded that 13 tyrosine residues of EYA3 can be phosphorylated in vitro by Src kinase: Y67, Y72, Y77, Y90, Y96, Y105, Y108, Y115, Y208, Y237, Y426, Y508, and Y532. Nine of them were detected in both experiments, but phospho-Y67 and Y532 were found only in the case of Src-phosphorylation time course (up to 6 h) phosphorylation of D311N (Figure 3b). Phospho-Y115 and Y508 were only identified in experiments shown in Figure 4. Three of these tyrosine residues (Y77, Y96, and Y237) were not completely dephosphorylated even after 6 h (Figure 3c) or 4 h (Figure 4b) of incubation with the catalytically active form of EYA3 (EYA3 full length or ED EYA3 WT), which strengthens the idea that these three residues have increased resistance to autodephosphorylation. Concerning Y508, although it was not detected as phosphorylated in the time course of in vitro reactions (Figure 3), the fact that it was still found phosphorylated after 4 h of incubation of phospho-EYA3 D311N with ED EYA3 WT (Figure 4b) suggests it is an additional site with increased resistance to autodephosphorylation.

### 2.4. Evaluation of the Identified Phosphotyrosine Sites’ Contribution to the Overall EYA3 Phosphorylation

Having identified 13 Src-phosphorylated tyrosine residues of EYA3, we aimed to elucidate their contribution to overall EYA3 phosphorylation. To address this, Y→F mutations of the previously identified phosphotyrosine residues were successively introduced in the sequence of EYA3 D309N (DN, inactive PTP mutant [2,22,23]) through site-directed mutagenesis (T0 to T11, Table S1). Both mutations, D309N and D311N have similar ability to inactivate dephosphorylation of EYA3. The contribution of a certain tyrosine residue to EYA3 phosphorylation can be evaluated by comparing two mutants that differ only by a single Y→F mutation at the tyrosine residue in question. Previously, we showed [30] that, when using single Y→F mutations in EYA3 D309N, we were unable to show the contribution of the phosphorylation of individual tyrosine residues to overall EYA3 phosphorylation. That is why, given the high number of identified phosphotyrosine sites, successive Y→F mutations were necessary to evaluate the cumulative contribution of several tyrosine residues to overall phosphorylation.

**Figure 5:**
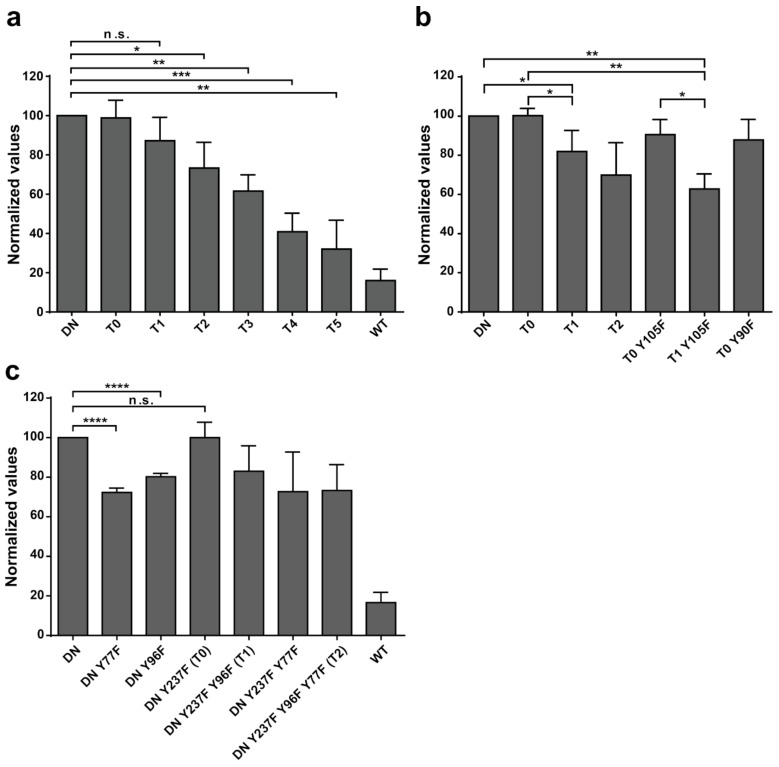
Tyrosine phosphorylation of various EYA3 constructs, co-transfected with c-Src Y527F in HEK293T cells. (**a**) EYA3 tyrosine-phosphorylation quantified for EYA3 D309N constructs containing up to six Y→F mutations. (**b**), (**c**) Quantification of EYA3 tyrosine-phosphorylation performed with the purpose of evaluating the relative contribution to the total EYA3 phosphorylation of the residues mutated in the EYA3 T4 mutant. C-Myc-EYA3 proteins were co-expressed with c-Src Y527F in HEK293T cells. After Western blot verification of the lysates, the EYA3 proteins were immunoprecipitated using anti-c-Myc antibody. Then the samples were verified by a Western blot. For each immunoprecipitated EYA3 construct, the intensity of the band obtained from the anti-pY Western blot was divided by the one from the anti-c-Myc Western blot. Then, every value obtained was normalized to the value corresponding to EYA3 D309N. Bar graphs represent values of the quantified phosphorylation of the samples normalized to the quantified phosphorylation of EYA3 D309N. Quantification of the Western blot bands was conducted using ImageJ software [45]. For each of the experiments described (**a**, **b**, **c**), the values represent the mean ± SD of three independent experiments (*n* = 3) with statistics (unpaired *t*-test, two-tailed) (n.s. for not significant, * *p* < 0.05, ** *p* < 0.01, *** *p* < 0.001, **** *p* < 0.0001).

Transient co-transfections of vectors encoding for EYA3 WT, EYA3 D309N, or its Y→F mutants and the highly active tyrosine-protein kinase Src (accession number P00523) with a Y527F mutation [43,44], were performed in HEK293T cells. Following immunoprecipitation (IP) of EYA3, quantified anti-pY Western blot analyses were performed for the EYA3 mutants (Figure 5). Notably, T0 to T3 contain Y→F mutations of residues identified as having increased resistance to autodephosphorylation (Y77, Y96, Y105, and Y237, Figure 3c, Figure 4b).

For DN proteins with up to six successive Y→F mutations, a gradual decrease in phosphorylation can be observed between DN and the consecutive Y→F mutants of this protein (from T1 to T5) (Figure 5a, Figure S14a). These data show that phosphorylation of tyrosine residues Y96, Y77, Y105, Y90, and Y115 has a measurable contribution but does not allow assessment concerning the relative impact of each individual residue. For all other mutants, obtained by introducing subsequent mutations into the sequence of T5 mutant (T6- to T11), quantification and normalization of the data revealed inconsistent variations of the phosphorylation level and no trend could be noticed because the relatively large number of mutations generated conformational changes that further affected EYA3′s phosphorylation propensity.

Aiming at a more accurate evaluation of the contribution of the phosphotyrosine residues identified on EYA3, additional mutants derived from DN, T0, and T1 were obtained and similar experiments were performed to evaluate their relative phosphorylation (Figure 5b,c, and Figure S14b). Thus, we observed that, among the mentioned tyrosine phosphorylation sites, Y77 and Y96 have the highest contribution (see the phosphorylation of T0 Y105F versus T1 Y105F, denoted T0 Y105F > T1 Y105F, DN > DN Y96F, and also DN > DN Y77F). Y237 does not seem to have any contribution to the EYA3 phosphorylation status (DN ~ T0, DN Y96F ~ T1, and DN Y77F ~ DN Y237F Y77F). No significant decrease in intensity was detected when Y90F or Y105F were introduced in the T0 mutant (T0 vs. T0 Y90F, T0 vs. T0 Y105F). The contribution of Y90 and Y105 can be observed only when residues such as Y77 and Y96, with high contributions to overall EYA3 tyrosine phosphorylation, are mutated (T1 > T2 > T3 > T4, Figure 5a).

The phosphorylation of constructs containing multiple Y→F mutations is generally not summing up the contributions of individual mutations. For example, the normalized value for T2 is similar to that of DN Y77F (Figure 5c), even though it contains mutations of two phosphosites (Y77 and Y96), with each one contributing to the overall EYA3 tyrosine phosphorylation levels. The explanation could be that phosphorylation of multiple tyrosine residues is not a sum of completely independent phosphorylation events. Possibly, phosphorylation of a given tyrosine residue is influenced by the other, previously phosphorylated tyrosine residues. From all the mutated tyrosine residues, Y77 and Y96 stand out by the fact that they have the highest contribution to EYA3 phosphorylation. This fact should be taken into consideration in future studies involving EYA3 such as in the identification and characterization of the signal transduction pathway.

### 2.5. Cellular Implications of Tyrosine to Phenylalanine Mutation of EYA3 Phosphotyrosine Sites

It has been shown that EYA proteins are implicated in cell transformation, proliferation, migration, and invasion [14,46,47]. In this regard, we investigated whether certain EYA3 tyrosine residues found phosphorylated by Src kinase could be implicated in any of these cellular events. In this respect, we focused on tyrosine residues of EYA3 having increased resistance to autodephosphorylation. Our reasoning was that these residues could be involved in signaling by tyrosine phosphorylation.

**Figure 6:**
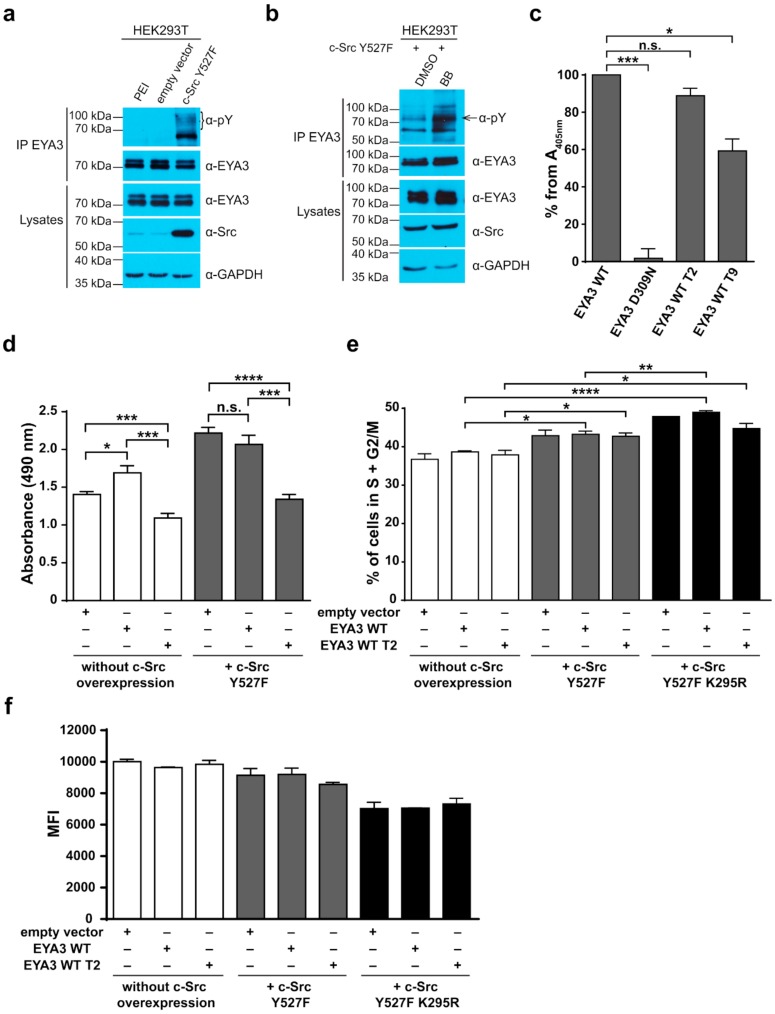
Cellular implications of tyrosine to phenylalanine mutation of EYA3 phosphotyrosine sites. (**a**) Endogenous EYA3 is tyrosine phosphorylated in HEK293T. Cells were transiently transfected with empty vector (pCiNeo), vector expressing c-Src Y527F (pSLX-c-Src Y527F) or treated only with the transfection reagent polyethylenimine (PEI). In lysates, EYA3, Src, and loading control (GAPDH) were detected by a Western blot. Endogenous EYA3 was immunoprecipitated and pY and EYA3 were detected by a Western blot. Two independent experiments were performed. (**b**) HEK293T cells, which overexpressed c-Src Y527F, were treated with benzbromarone (BB) versus dimethyl sulfoxide (DMSO, solvent for BB) to prove endogenous EYA3′s autodephosphorylation capacity. Src overexpression was detected by a Western blot, as were EYA3 and the loading control GAPDH. Endogenous EYA3 was immunoprecipitated and anti-pY and anti-EYA3 antibodies were used in a Western blot for the detection of tyrosine phosphorylated EYA3. Results show that EYA3 tyrosine phosphorylation (indicated by an arrow) is much more intense when BB is added in the cell culture media. Two independent experiments were performed. (**c**) Protein tyrosine phosphatase activity of two Y→F mutants of EYA3 tested using the synthetic substrate para-nitrophenylphosphate (pNPP). Myc-tagged EYA3 WT, D309N, WT T2, and WT T9 were transiently expressed in HEK293T cells. After immunoprecipitation (using anti-c-Myc antibody, 9E10), proteins were subjected to an in vitro PTP assay using pNPP as a substrate. The EYA3 WT T2 protein retained 90% of its PTP activity, whereas EYA3 WT T9 retained only 60%. Three independent experiments were conducted, with each having three technical replicates per sample (*n* = 9). For each sample, the absorbance at 405 nm (A_405 nm_) was measured. The results shown in the graph represent the mean ± SEM of the values obtained after normalization to the A_405 nm_ of the EYA3 WT sample. Statistical analysis was made by applying unpaired Student’s t-test (two-tailed) (n.s. - not significant, * *p* < 0.05, and *** *p* < 0.001). (**d**) The MTS proliferation assay of HEK293T cells at 72 h after transfection. The results represent mean ± SEM of three independent experiments, each having three replicates (*n* = 9). Statistical analysis was performed by applying unpaired Student’s *t*-test (two-tailed, * *p*< 0.05, *** *p*< 0.001, and **** *p* < 0.0001). (**e**) Flow cytometry analysis of the cell cycle using HEK293T cells transiently expressing EYA3 WT or EYA3 WT T2 with or without co-expression of c-Src Y527F or c-Src Y527F K295R at 24 h post-transfection. The values represent the cumulated value of cell percentages in the S and G2/M phases. The results represent the mean ± SEM of two independent experiments (*n* = 3). Statistical analysis was performed using unpaired Student’s t-test (two-tailed, * *p* < 0.05, ** *p* < 0.01, and **** *p* < 0.0001). (**f**) Flow cytometry analysis of HEK293T cells stained with carboxyfluorescein succinimidyl ester (CFSE). Bar graph shows the median value of the CFSE intensity (MFI) 48 h after transfection. Values represent the mean ± SD of one representative experiment out of two performed (*n* = 2).

First, we tested whether the endogenous (active) EYA3 is tyrosine phosphorylated in HEK293T cells transiently expressing c-Src Y527F. EYA3 was found tyrosine phosphorylated only when constitutively active Src was overexpressed in HEK293T cells (Figure 6a). In a similar experiment, in which HEK293T cells overexpressing c-Src Y527F were treated with benzbromarone (inhibitor of EYA’s PTP activity, noted BB) [48], an increase of endogenous EYA3′s tyrosine phosphorylation was observed as compared to the control cells (Figure 6b). This finding proves that endogenous EYA3 has the capacity of autodephosphorylation. 

Next, we evaluated whether EYA3 proteins containing multiple Y→F mutations still retain the PTP activity. Based on this reason, we analyzed the catalytically active form of EYA3 T9 (containing all identified Y→F mutations from the N-terminal region) and EYA3 T2 (including mutations of only those tyrosine residues that had increased resistance to autodephosphorylation). The PTP activity of EYA3 WT T2 decreased by only 10% whereas that of EYA3 WT T9 was 40% lower than that of EYA3 WT (Figure 6c). Consequently, only EYA3 WT T2 was chosen for further investigations because it retained most of the PTP activity. This mutant was studied in parallel with EYA3 WT, in HEK293T cells, with and without c-Src Y527F overexpression.

We assessed the proliferative capacity of HEK293T cells overexpressing EYA3 WT or EYA3 WT T2 (Figure S15) using a standard 3-(4,5-dimethylthiazol-2-yl)-5-(3-carboxymethoxyphenyl)-2-(4-sulfophenyl)-2H-tetrazolium, inner salt (MTS) assay. Results showed that, in the absence of c-Src Y527F co-expression, cells which expressed an EYA3 WT T2 mutant had proliferation rates lower than those expressing EYA3 WT or the control (empty vector) (*** *p* < 0.001, Figure 6d). This fact suggests that one, two, or potentially all these tyrosine residues could be involved in EYA3-induced proliferation events. On the other hand, we observed a stimulatory effect of EYA3 WT overexpression on the proliferative rate of HEK293T cells (* *p* < 0.05, Figure 6d), which was abolished by the Src-induced phosphorylation of EYA3 WT (cells co-expressing c-Src Y527F and EYA3 WT had proliferation rates similar to cells overexpressing c-Src Y527F only, Figure 6d).

Because the MTS assay results could be explained not only by increased proliferation rates but also by increased cellular survival or metabolism, we performed cell cycle distribution analysis, aiming to investigate if EYA3 plays a role in HEK293T cell proliferation. The experiments were performed using cells that overexpressed EYA3 WT or WT T2 in three different contexts: alone or together with c-Src Y527F or a dominant negative form of the kinase (c-Src Y527F K295R, inactive kinase) (Figure S16). Cell cycle flow cytometry analysis performed 24 h after transfection (Figure 6e, Figure S16a) revealed that the transient expression of the dominant negative form of c-Src generated the highest proportion of cells in the S + G2/M phases. The smallest proportion of cells in the S + G2/M phases was detected when no c-Src forms were overexpressed. In the presence of c-Src Y527F K295R, EYA3 WT overexpression generated a higher number of cells in the S and G2/M phases than EYA3 WT T2. Interestingly, the accumulation in G0/G1 (reduced number of cells in S + G2/M) generated by EYA3 WT T2 was not observed when the constitutively active form of c-Src was overexpressed. Next, we verified if the increase in the number of cells in the S + G2/M phases was caused by a higher proliferation rate and not by an arrest of the cells in the G2/M phase. To assess this, we stained cells with carboxyfluorescein succinimidyl ester (CFSE) and followed their proliferation by measuring dye dilution with time. Flow cytometry analysis (Figure 6f, Figure S17) showed that, in the absence of c-Src overexpression, cells had the lowest proliferation rates (highest median fluorescence intensity (MFI) values), while overexpression of the dominant negative form of c-Src generated cells with the highest proliferation rates. The MFI values from the CFSE experiment (obtained 48 h post-transfection) reflect the cell cycle distribution (measured 24 h post-transfection). This means that the observed cellular proliferation profiles, as illustrated in Figure 6d, are a consequence of the variations in the cell cycle.

Taken together, our data indicate that modification of the proliferation rates obtained in our MTS assay reflects true modification of the cell cycle distribution and the Y → F mutation of the three residues Y77, Y96, and Y237 abolishes the pro-proliferative effect of EYA3 overexpression. 

### 2.6. Src-Induced Phosphorylation Sites of EYA3 Overexpressed in HEK293T Cells

Based on our results concerning modulation of the cell proliferation as generated by EYA3′s triple Y→F mutant in HEK293T, we aimed to identify the phosphorylation pattern of EYA3 in this cell line. Three EYA3 proteins were subjected to our investigation: the enzymatically active WT protein and the PTP inactive mutants DN and DN Y77F. The latter construct contains the Y→F mutation of the tyrosine residue displaying the highest contribution to EYA3 phosphorylation. In HEK293T, we overexpressed EYA3 proteins with and without co-expression of c-Src Y527F, respectively (Figure S18). EYA3 peptide samples, obtained after phosphopeptide enrichment using TiO_2_ were analysed by nLC-MS/MS with CID and ETD fragmentation methods.

Table 1 shows phosphorylated residues of EYA3 from HEK293T cells. Most of the phosphotyrosine residues identified are the same as the ones found in our in vitro analysis of time-dependent phosphorylation of EYA3 D311N except for phospho-Y325, which is the only tyrosine phosphorylation site from the ED. No tyrosine residue was identified as phosphorylated in the absence of c-Src Y527F overexpression. Three tyrosine residues - Y77, Y108, and Y208 - have an increased resistance to autodephosphorylation. These residues are found phosphorylated in the case of overexpressing EYA3 WT with c-Src Y527F.

Given that TiO_2_ enrichment in phosphopeptides (applied on a proteolytic digest of samples) is not specific for phosphotyrosine containing peptides, we obtained information about phosphoserine residues as well. Three phosphoserine residues were found in HEK293T cells in all overexpressed EYA3 constructs: S138, S225, and S438. S157 was detected as phosphorylated in HEK293T only in the presence of overexpressed c-Src Y527F. As a possible explanation, Src tyrosine kinase could indirectly stimulate S157 phosphorylation. S438 was also identified as phosphorylated in two cancer cell lines, HeLa and K562, in a large scale mass spectrometry analysis in which different strategies were implemented to improve the phosphoproteome coverage and characterization [49]. 

The majority of the Src tyrosine phosphosites of EYA3 found in HEK293T (phospho-Y325 being the only exception) support the results from our in vitro experiments.

## 3. Discussion

In this article, we report the detection and detailed analysis of Src kinase-phosphorylated tyrosine residues of EYA3 as well as EYA3′s capacity to autodephosphorylate these sites. 

Initially, native MS experiments revealed 12 phosphorylated tyrosine residues on EYA3 D311N. Monophosphorylated EYA3 WT molecules were detected after a 2-h incubation period with Src, which proves the autodephosphorylation capacity of the PTP and also that autodephosphorylation exceeds Src phosphorylation on these tyrosine residues. Other examples of PTPs capable of tyrosine autodephosphorylation include RPTPα [50], PTP1C [51], CD45 [52], and PTP2C (SHP-2) [53,54,55]. Regarding the potential role of tyrosine phosphorylation, it has been reported that this post-translational modification produced specific effects like modulation of PTP activity [50,53,54,55], which generates binding sites for SH2-containing proteins [50] or induces various signal transduction processes [51,52,53]. From our perspective, the present work represents the first in-depth analysis of tyrosine phosphorylation and dephosphorylation sites of an EYA homologue.

Native MS analysis of purified EYA3 proteins, pinpointed by nLC-MS/MS experiments (Figures S1, S2 and S7), revealed several interesting facts: lack of the start methionine, acetylation of the second serine residue, and presence of two chaperons, Skp and DnaK. The first one actually forms a complex with EYA3. The N-terminal methionine cleavage in prokaryotic proteins has been shown to be promoted by the presence of Ser, Ala, Gly, Pro, and Thr residues in the second position [56]. On the other hand, N_α_-acetylation is a rare phenomenon in *E. coli* [57,58,59,60,61]. N-terminal acetylation of recombinant mammalian proteins expressed in *E. coli* has also been reported [62]. Analysis of different purified EYA3 forms (WT full length, N-terminal and C-terminal) and mutants (D309N, D311N) showed that association with Skp is dependent on the presence of the N-terminal region of the protein (Figure S19). Accounting for the increased disorder of the N-terminal region of EYA3 (see PrDos [63] prediction of disordered regions in EYA3 - Figure S20) as well as for our finding that EYA3 could not be expressed in MC 4100 ΔSkp cells, we can assume that Skp plays a role in the stabilization of the N-terminal region of EYA3. A lack of a stabilizing effect of Skp in MC 4100 ΔSkp leaves disordered regions exposed to cellular proteases, which leads to proteolysis of the expressed EYA3.

The experiment concerning the time-course of the in vitro phosphorylation shows that a total of 11 tyrosine residues were phosphorylated on EYA3 D311N (Y67, Y72, Y77, Y90, Y96, Y105, Y108, Y208, Y237, Y426, and Y532). Furthermore, most phosphorylated residues belong to the N-terminal part of the protein. In the case of EYA3 WT, four tyrosine residues remained phosphorylated (Y77, Y96, Y105, and Y237) even after 6 h, which suggests that these sites have a higher resistance to autodephosphorylation. The fact that, around the 2-h time point, EYA3 autodephosphorylation dominates Src phosphorylation is supported by our native mass spectrometry experiments, in which only EYA3 WT proteoforms with one phosphorylated residue were detected after 2 h of incubation with Src. Moreover, kinetics experiment reveals a steady state process in the case of time-course phosphorylation of EYA3 WT by Src. EYA3 tyrosine phosphorylation seems to be under the dynamic control of three essential factors: (i) time-dependent EYA3 phosphorylation by Src, (ii) EYA3 autodephosphorylation activity, and (iii) moderate increase of EYA3 tyrosine phosphatase activity upon its Src phosphorylation. The situation becomes even more complicated if we take into account that both Src phosphorylation and EYA3 autodephosphorylation are site-specific. This interplay might produce transient modifications of the phosphorylation pattern of EYA3, which is in agreement with modifications observed in our time dependence experiments described in Figure 3. We consider that the interplay between the mentioned factors confers EYA3 a phosphorylation pattern, which can be modified according to the physiological needs of the cell.

Experiments on the dynamics of Src-dependent phosphorylation of EYA3 D311N, analyzed using nLC-MS/MS, demonstrate that, out of 11 phospho-tyrosine residues, after 4 h of incubation with ED EYA3 WT, only four tyrosine residues remained phosphorylated, which include Y77, Y96, Y237, and Y508. Notably, three of these residues are the most resistant to autodephosphorylation in the experiment described in Figure 3c. The intensive autodephosphorylation of Y105 outlined in the in vitro phosphorylation time-course at the 2-h time point (Figure 3c, Src active) explains its eventual dephosphorylation after 2 h of incubation of D311N with ED EYA3 WT (after Src inactivation with PP2) (Figure 4b). In this time frame (0 to 4 h incubation with ED EYA3 WT), Y508 was shown to be a residue with high resistance to autodephosphorylation (Figure 4b). In our previously published data [30], it is characterized as the only autodephosphorylation residue from the ED region of EYA3, which means that, in vivo, this residue is eventually autodephosphorylated. The apparent contradiction can be explained by the fact that, under in vitro conditions, phosphorylated Y508 has been exposed for a limited time to EYA3 autodephosphorylation activity. Under in vivo conditions, this phosphosite is exposed to long-term autodephosphorylation activity, which leads to complete removal of its phosphate residue. In addition, under in vivo conditions, there are other factors (including inhibitors, activators, etc.), which may modulate Src phosphorylation and/ or EYA3 phosphatase activity.

Additional experiments illustrate the significance of Y77 and Y96 on overall EYA3 tyrosine phosphorylation (Figure 5). Residue Y77 seems to have the highest contribution to the total phosphotyrosine signal followed by Y96. On the contrary, Y237 proved to have a very small contribution to the phosphorylation signal. Variations regarding the phosphorylation pattern of different EYA3 D309N Y→F mutants denote the complex nature of the processes (including phosphorylation) in a mammalian cell line versus in vitro. This was also proven through the results obtained in our proliferation assays and cell cycle analysis. Proliferation assays (Figure 6d,f) showed that c-Src Y527F co-expression in HEK293T cells induced an increased proliferation that masked the contribution of EYA3 WT to this process. This effect was also illustrated by flow cytometry analysis of the cell cycle. The trend toward an increase in the S and G2/M phases of cells overexpressing EYA3 WT versus mock transfected cells was abolished when EYA3 WT was co-expressed with c-Src Y527F. On the other hand, the MTS assay and cell cycle distribution analysis revealed that, by mutating three tyrosine residues from EYA3 (Y77, Y96, and Y237) to phenylalanine, the pro-proliferative effect of EYA3 was abolished. These results request further investigations in non-transformed as well as cancer cells, where it was reported that EYA proteins promote processes related to tumor progression. It remains to be elucidated which of these three tyrosine residues, if not all of them, are implicated in cell proliferation and what is the molecular mechanism generating this effect.

Comparing the phosphorylation pattern of EYA3 WT co-expressed with c-Src Y527F in HEK293T cells to its pattern when only EYA3 WT was expressed (Table 1), we noticed that four residues were still phosphorylated in the presence of c-Src Y527F co-expression (Y77, Y108, Y208, and S157). Y77 emerged as a residue with increased resistance to autodephosphorylation. It is the only tyrosine residue detected as phosphorylated in EYA3 WT in the presence of Src, both in vitro as well as in HEK293T cells. Twelve phospho-residues are reported in PhosphoSitePlus^®^ [64]: S256, S262, S264, S266, T268, T269, S271, T274, T295, S297, S438, and S472. Among all of them, S438 is the only phospho-residue also detected in our experiments made on HEK293T cells. The multitude and variety of phosphosites detected on EYA3 in different studies suggests that this protein is involved in many cellular processes.

**Figure 7:**
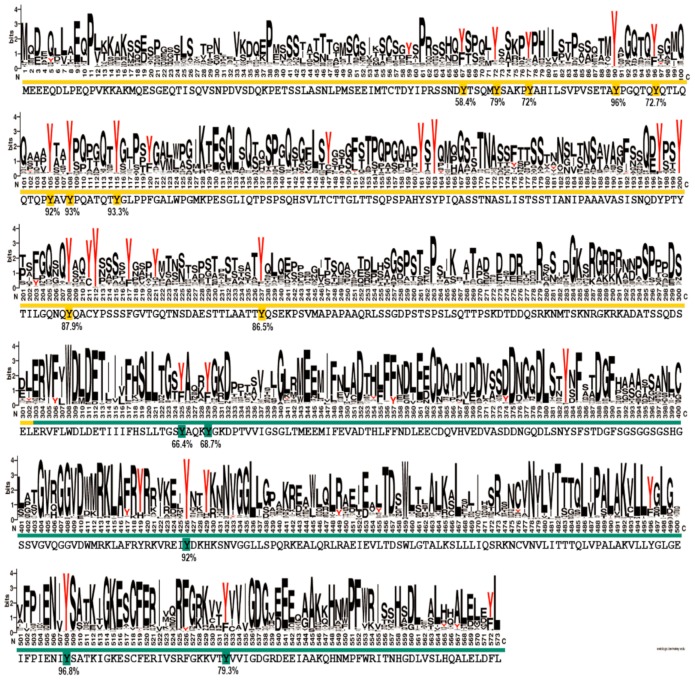
Sequence conservation logo of EYA3 721 homologues from RP55 database, expressed as relative entropy (bits). Higher heights show a higher degree of conservation at that position in sequence. Tyrosine residues detected as phosphorylated in the manuscript are highlighted in yellow in the N-terminal sequence and in green in the Eya Domain (ED), respectively. The relative frequency of these tyrosine residues is also specified below each highlighted residue (percent of tyrosine occurrences from the total alignment, excluding the gaps). The sequence conservation logo shows the relative entropy, i.e., the amount of information (bits) encoded by every position in the amino acid sequence. Higher heights of an amino acid letter correspond to a higher divergence from the background distribution of amino acids and indicate a higher conservation of that particular site.

Pertaining to the phosphotyrosine residues identified in this study, an important question could be raised as to which of these sites are functionally relevant. In principle, in vitro identification of a phosphorylation site does not necessarily mean a particular phosphosite has a functional role. On the contrary, the result might be only an experimental artefact. An argument resides in the fact that, while thousands of phosphosites have been identified, only several hundred have a clear function [65]. Therefore, it is important to predict which potential phosphosites actually have functional roles. In this respect, there are numerous reports proving that functional phosphosites are significantly more conserved than phosphosites with unknown functions [65]. Apparently, tyrosine residues situated in disordered regions of proteins are frequently off-target phosphorylation sites [66], whereas it has also been shown that phosphosites are enriched in disordered regions in order to be recognized more easily by the cognate kinase [67,68,69]. According to our protein disorder prediction (Figure S20), the N-terminal region of EYA3 is a good candidate for both hypotheses. To make a more accurate prediction, we sought to determine the degree of conservation of the identified EYA3 tyrosine phosphorylation residues (i.e., fifteen sites: Y67, Y72, Y77, Y90, Y96, Y105, Y108, Y208, Y237, Y325, Y329, Y426, Y508, and Y532) assuming that functional sites should be conserved to a higher extent. We performed a variability study on a group of 721 homologues of human EYA3, selected from the Representative Proteomes RP55 database [70] (Figure 7). This gives us a better view of the conserved residues among the 721 sequences, but, most importantly, the possibility of estimating the degree of conservation for EYA3 tyrosine residues. The fact that, in the N-terminal and ED regions of the protein, tyrosine residues are conserved to a higher extent, means they could be more likely implicated in physiological processes. Individual contributions of these residues definitely warrant further investigation. Y72, Y77, Y90, Y105, Y108, and Y208 display high conservation. The fact that these residues were found as phosphosites in HEK293T cells suggests they have a high probability of being functionally relevant. Phosphoresidues specific to one cell line could be part of signal transduction pathways which, among other mechanisms, lead to different effects on proliferation or other cellular events. The tyrosine residues that were not detected as phosphorylated by Src kinase but have a high degree of conservation could also be functional phosphosites. Further experiments should confirm the reliability of these predictions. 

The 13 tyrosine phosphosites detected on EYA3 are not uniformly distributed along its sequence, with the N-terminal part of the protein being preferred for phosphorylation. These residues differ by their time-dependent phosphorylation kinetics, resistance to autodephosphorylation, and contribution to the overall tyrosine phosphorylation of EYA3. Three of these tyrosine residues (Y77, Y96, and Y237) are implicated in EYA3-dependent proliferation events in HEK293T. As supported by our results reported in this article, EYA3 displays a specific, time-dependent phosphorylation pattern that is under the control of Src phosphorylation and EYA3 autodephosphorylation activity. Apparently, this phosphorylation pattern can influence important physiological processes.

## 4. Materials and Methods

### 4.1. Antibodies, Enzymes, Reagents, and Kits

Antibodies used in Western blotting: anti-pY (pY99, sc-7020 HRP), anti-GAPDH (FL-335) HRP (sc-25778 HRP), anti-c-Myc (9E10) HRP (sc-40 HRP), and secondary antibodies goat anti-rabbit IgG-HRP (sc-2004) and rabbit anti-mouse IgG-HRP (sc-358914) from Santa Cruz Biotechnology, Dallas, TX, USA, anti-His antibody (27-4710-01), from GE Healthcare, Chicago, IL, USA, anti-v-Src (clone 327) from Merck Millipore, Burlington, MA, USA, and anti-Actin Ab-5 (Clone C4/actin) from BD Biosciences, Bedford, MA, USA. Anti-c-Myc (9E10) (sc-40) from Santa Cruz Biotechnology and anti-myc tag (clone4A6) from Merck Millipore were used for immunoprecipitation (IP). Anti-EYA3 (21196-1-AP) from Proteintech (Rosemont, IL, USA) was used in Western blot and IP experiments. The Western Blotting Chemiluminescence Luminol Reagent (sc-2048) from Santa Cruz Biotechnology, USA was used for detecting immunoreactive bands. GST-v-Src active kinase protein (S19-10G) was purchased from SignalChem Biotech Inc. (Richmond, BC, Canada). 

For mass spectrometry, we used LC-MS chromasolv solvents (water, acetonitrile–noted MeCN), ammomium bicarbonate, iodoacetamide (I6125), and dithiothreitol (DTT, 43815) from Sigma-Aldrich (St. Louis, MO, USA), formic acid, eluent additive for LC-MS (56302) from Fluka (Honeywell, Romania), sequencing grade Trypsin, Chymotrypsin, and rLysC from Promega, Madison, WI, USA, C18 bulk (Aqua 5 µm C18 200Å bulk, Phenomenex, Torrance, CA, USA), C8 material (3M™ Empore™ C8 disks), Titansphere™ Phos-TiO Kit, 1mg TiO_2_/ 10µL tip (5010-21309), GL-Tip SDB (7820-11200), and GL-Tip GC (7820-11201) all from GL Sciences (Tokyo, Japan) and Amicon Ultra-0.5mL Centrifugal Filter Unit with Ultracel-30 membrane, from Millipore (USA). 

Other reagents and kits include: QuickChange Lightning Site-Directed Mutagenesis Kit (#210519), from Agilent Technologies (Santa Clara, CA, USA), CellTiter 96^®^ AQ_ueous_ One Solution Cell Proliferation Assay System (Promega, USA), Vybrant™ CFDA SE Cell Tracer Kit (V12883, Thermo Fisher Scientific, Dreieich, Germany), Protein G Sepharose^®^ 4 Fast Flow (17-0618-01), GE Healthcare Life Sciences (USA) and also Rec-Protein A Sepharose^®^4B Conjugate (101141), Invitrogen (Carlsbad, CA, USA) - for IP, 3-(N-morpholino)propanesulfonic acid (MOPS, A2947) and phenylmethylsulfonyl fluoride (PMSF, A0999) from AppliChem (Darmstadt, Germany), ethylenediaminetetraacetic acid (EDTA, ED255), adenosine 5′-triphosphate disodium salt (Na_2_ATP, A3377) and polyethylenimine (PEI, 40872-7) from Sigma-Aldrich (USA), Tris-base (648311) and dimethyl sulfoxide (DMSO, 317275) from Calbiochem (San Diego, CA, USA), NP-40 (A1694) from Panreac AppliChem (Germany), protease inhibitor cocktail tablets (sc-29131), and sodium orthovanadate (sc-3540) from Santa Cruz Biotechnology, USA, iodoacetic acid (35603, Pierce™, USA), NativeMark™ Unstained Protein Standard and Spectra™ Multicolor Broad Range Protein Ladder (Thermo Fisher Scientific, Dreieich, Germany), Low Molecular Weight Marker (GE Healthcare, USA), BLUelf Prestained Protein Ladder (GeneDireX-PM008-0500, Taiwan, China), RNAse (R6513, Sigma-Aldrich, USA), propidium iodide solution (S7112, Sigma-Aldrich, USA), and sodium citrate (S4641, Sigma-Aldrich, USA). 

The cell culture includes Dulbecco’s Modified Eagle Medium (DMEM) (4.5 g/L D-Glucose)-GlutaMax™ media (61965059), Fetal Bovine Serum (FBS) (16000044), 100x MEM Non-Essential Amino Acids solution (NEAA) (11140), 100 mM sodium pyruvate (11360070), and 1x phosphate-buffered saline (PBS) (18912-014), which were all from Gibco (Thermo Fisher Scientific, Dreieich, Germany). 

### 4.2. Cell Culture, Transfection, Immunoprecipitation and Western Blotting

HEK293T cells were cultured at 37 °C in a 5% CO_2_ atmosphere incubator, in DMEM (4.5 g/L D-Glucose)-GlutaMAX™ media, which also contained 10% heat inactivated FBS, 1 mM sodium pyruvate, and 1x NEAA. 

The cells were passaged 24 h before transfection, so they would be at 70% confluency the day of the experiment. Transient co-transfections were carried out using PEI as a transfection reagent.

Unless stated otherwise, cell harvesting and lysis were performed as described. The cells were washed two times with ice-cold PBS 1× and then harvested on ice in 50 mM Tris-HCl pH 7.4, 150 mM NaCl, 10 mM MgCl_2_, 1% NP-40, 10% glycerol, 1× protease inhibitor cocktail, 1 mM PMSF (lysis buffer) supplemented with 1 mM sodium orthovanadate and 5 mM iodoacetic acid and lysed by passing the cellular suspension three times through a syringe with a narrow inner diameter, vortexing and incubation for 20 min on ice. After 25 min of centrifugation at 20,000× *g* at 4 °C, the soluble cellular extract was collected, and the total protein concentration was determined by the bicinchoninic acid (BCA) assay. Western blot analyses of the lysates were carried out to verify the expression of each of the proteins of interest (endogenous EYA3, c-myc tagged EYA3, Src, and loading control—G-actin or GAPDH) using anti-EYA3 (21196-1-AP) with goat anti-rabbit IgG-HRP (sc-2004), anti-c-Myc-HRP (sc-40 HRP), anti-v-Src (clone 327), and anti-actin (Ab-5, Clone C4/actin) both with rabbit anti-mouse IgG-HRP (sc-358914), and anti-GAPDH-HRP (sc-25778 HRP) antibodies.

IP of c-myc tagged EYA3 or the endogenous EYA3 protein was achieved by incubating the lysates with anti-c-Myc (9E10, sc-40) or anti-EYA3 (21196-1-AP) antibody for 1 h at 4°C, with rotation, which was followed by the addition of Protein G or Protein A Sepharose beads and further incubation with a rotation for 16 h at 4 °C. The next day, beads were washed three times with 50 mM Tris-HCl pH 7.4, 150 mM NaCl, 10 mM MgCl_2_, 1% NP-40, and 10% glycerol (wash buffer). Proteins were eluted by adding 1x Laemmli sample buffer and incubating the beads for 5 min at 95 °C. The eluates were analysed by a Western blot using the following antibodies: anti-pY (pY99, sc-7020 HRP), then anti-c-Myc (sc-40 HRP) for c-myc tagged EYA3, respectively, and anti-EYA3 antibody (21196-1-AP) with goat anti-rabbit IgG-HRP (sc-2004), for endogenous EYA3. All statistical analyses were conducted using GraphPad Prism6. 

Evaluation of the tyrosine phosphorylation of EYA3 D309N Y → F mutants. Transient transfections were performed in six well plates. The pCS2-MT vector containing the sequence for myc-tagged EYA3 D309N, its corresponding Y → F mutants or EYA3 WT, along with pSLX-c-Src Y527F, were transfected at a ratio of 5 to 1 (2 µg: 0.4 µg). The EYA3 D309N Y → F mutants were obtained by site directed mutagenesis (QuickChange Lightning Site-Directed Mutagenesis Kit). After 24 h, the cells were harvested, lysed, and Western blot analyses of the lysates were performed using anti-c-Myc (sc-40 HRP), anti-v-Src (clone 327), and anti-Actin Ab-5 (Clone C4/actin) with rabbit anti-mouse IgG-HRP (sc-358914) as a secondary antibody. Transiently expressed c-myc-EYA3 protein was immunoprecipitated using the anti-c-Myc (9E10) antibody. The resulting samples were analysed by a Western blot using anti-pY (pY99, sc-7020 HRP) and anti-c-Myc (sc-40 HRP) antibodies. Quantifications were conducted using ImageJ software [45]. Three independent experiments were performed.

The following describes the analysis of endogenous EYA3 tyrosine phosphorylation. Before transfection, cultured HEK293T cells were passaged in 25 cm^2^ flasks. Cells received the following treatment: transfection reagent (PEI) and transient transfection with empty vector (pCiNeo) and pSLX-c-Src Y527F, respectively. Forty hours post-transfection, the cells were harvested and lysed. Following Western blot verification, lysates were subjected to IP using anti-EYA3 antibody (21196-1-AP). The resulting samples were analyzed by a Western blot using anti-pY (pY99, sc-7020 HRP) and anti-EYA3 antibody (21196-1-AP) with goat anti-rabbit IgG-HRP (sc-2004). Two independent experiments were performed.

The following describes the inhibition of endogenous EYA3 PTP activity. Cells were cultured in the same conditions as in the previous experiment. Two flasks were transfected with vector pSLX-c-Src Y527F. Twenty-four hours after having induced transient expression of c-Src Y527F, one of the flasks was treated with 10 μM BB (solubilized in DMSO) and the other one with the corresponding volume of DMSO. In addition, 24 h after treatment, cells were harvested, lysed, and lysates were analysed by Western blotting using anti-EYA3 (21196-1-AP) with goat anti-rabbit IgG-HRP (sc-2004), anti-v-Src (clone 327) with rabbit anti-mouse IgG-HRP (sc-358914), and anti-GAPDH-HRP (sc-25778 HRP) antibodies. The IP of endogenous EYA3 and its Western blot analysis was performed in the same manner as in the previous experiment. Two independent experiments were performed.

All immunoreactive bands were detected by chemiluminescence using Western Blotting Chemiluminescence Luminol Reagent (sc-2048) from Santa Cruz Biotechnology, USA.

### 4.3. Expression and Purification of N-Terminal His-Tagged EYA3 Proteins

Full length proteins (6xHis-EYA3 WT, 6xHis-EYA3 D309N, and 6xHis-EYA3 D311N) as well as the N-terminal and C-terminal fragments of EYA3 protein (6xHis-ΔED EYA3 WT, 6xHis-ED EYA3 WT) were expressed in a prokaryotic system (*E. coli* BL21(DE3)RIL strain) and purified following the protocols described in previous studies [30,71].

### 4.4. In Vitro Src Kinase Reactions

Kinase reactions were performed at 30 °C in 25 mM MOPS pH 7.2 with 10 mM MgCl_2_ and 0.25 mM DTT (kinase assay buffer). The molar ratio between the EYA3 protein and GST-v-Src was 50:1. Na_2_ATP was added in a 150-fold molar excess compared to the concentration of EYA3. Reactions were quenched on ice by the addition of EDTA so that the molarity in the reaction was five times that of MgCl_2_.

### 4.5. Native MS

The following describes the analysis of purified 6xHis-EYA3 WT and 6xHis-EYA3 D311N. Proteins stored at −80 °C were thawed on ice and then centrifuged at 20,000× *g* for 20 min at 4 °C. The soluble protein sample was buffer exchanged to ammonium acetate at a pH of 6.8. Each protein sample was subjected to native mass spectrometry analysis, ESI-MS, using the Orbitrap™ Exactive™ Plus EMR (Thermo Fisher Scientific, Germany) instrument. Interpretation of the raw data obtained was conducted using Xcalibur™ Software (Thermo Fisher Scientific) and MassLynx™ v4.1 Software (Waters Corporation, Milford, MA, USA). In vitro v-Src phosphorylated 6xHis-EYA3 WT and 6xHis-EYA3 D311N (for 5 min and 2 h) were also analyzed as described above.

The following describes the native MS analysis of direct confirmation of dephosphorylation. His-tagged EYA3 D311N in vitro tyrosine phosphorylation by GST-v-Src was performed as described earlier. Samples were taken 5 min and 2 h after the addition of v-Src. The remaining reaction was split in half with one sample being further incubated at 30 °C and the other one was incubated at the same temperature with EYA3 WT in a ratio of 5.61:1 for 2 more hours. All reactions were quenched on ice by adding EDTA to a final concentration that was five times higher than that of MgCl_2_. The buffer was exchanged to ammonium acetate pH 6.8 and samples were analyzed by native ESI-MS (Orbitrap™ Exactive™ Plus EMR, Thermo Fisher Scientific, Germany). Raw data were acquired and analyzed using Xcalibur™ Software (Thermo Fisher Scientific). The peaks corresponding to 6xHis-EYA3 D311N molecules carrying charge 16+ were used to quantify the evolution of tyrosine phosphorylation. The intensity of each peak was taken into consideration for quantification. The acetylated molecules were separately quantified. The data were quantified by taking into consideration the signal for EYA3 WT added in one of the samples (dephosphorylation reaction).

### 4.6. nLC-MS/MS Detection of Phosphorylated Residues and Mass Spectrometry Data Analysis

#### 4.6.1. Detection of Phosphorylated and Autodephosphorylated Tyrosine Residues

6xHis-EYA3 WT and, respectively, 6xHis-EYA3 D311N were incubated either with ATP or with GST-v-Src and ATP, at 30 °C. The kinase reactions were performed as previously mentioned, with 10 µM EYA3 in a 10-µL reaction volume. The reactions were quenched with EDTA, at several time points: 5 min, 30 min, 2 h, and 6 h, and then stored at −80 °C. Regarding the in-solution digestion, after thawing them on ice, the same volume from a buffer containing 8 M urea in 100 mM Tris, pH 8 was added to the samples in order to aid the digestion. Reduction was performed by incubation in 2 mM DTT final concentration for 40 min at 60 °C and alkylation was achieved by incubating in 5 mM iodoacetamide for 40 min, at 25 °C, in the dark. Before adding the protease, the samples were diluted with 100 mM Tris pH 8 to a final volume of 100 µL and the EDTA concentration was adjusted to 20 mM in order make sure Mg^2+^ ions (now at 2.5 mM) were complexed, which inhibits any phosphorylation or autodephosphorylation (EYA’s cofactor is Mg^2+^) from taking place. Sequencing grade chymotrysin was added in a 1:100 (*w/w*) ratio with EYA3 protein and incubated for 15 h at 25 °C. The resulting samples were acidified to 10% formic acid (FA) and peptides were desalted using tips with C18 bulk plugged with C8 material, manufactured in the laboratory. After elution from the tips in 100% MeCN, peptides were dried in a vacuum concentrator to be stored at −80 °C until nLC-MS/MS analysis. In nLC-MS/MS analysis, the samples were analyzed by reversed phase nLC-MS/MS using an LTQ-Orbitrap Elite™ mass spectrometer coupled to a Proxeon EASY-nLC 1000 (nano-high performance liquid chromatograph, Thermo Fisher Scientific). Peptides were resuspended and loaded on the C18 analytical column (75 μm particles) in solvent A (0.1% (*v/v*) FA in H_2_O) and the elution was achieved over a 55-min gradient of solvent B (0.1% (*v/v*) FA in MeCN). Precursor and fragment ion spectra were acquired in the Orbitrap analyzer. Gas phase fragmentation was obtained by applying HCD on the 10 most abundant precursor ions, rejecting those that had a charge state of 1+. Three technical replicates were performed from each sample (*n* = 3). Peptides were identified using Proteome Discoverer v1.4 (Thermo Fisher Scientific) with Sequest HT as an algorithm and the precursor ions area detector node was used to determine the area of each peptide ion. The searches were performed using a modified version of the Human database from UniProtKB/ Swiss-Prot, containing the sequences of purified recombinant EYA3 proteins, and Skp and DnaK. Other settings included: chymotrypsin (full) as enzyme, with a maximum of five missed cleavages [72] (we found that it did not cut at C-terminal of pY), precursor mass tolerance of 10 ppm, fragment mass tolerance of 0.05 Da, serine, threonine, and tyrosine phosphorylation (+79.966 Da) and also methionine oxidation (+15.995 Da) as dynamic whereas carbamidomethylation (+57.021 Da) on cysteine residues as static modification. A decoy database search having reversed protein sequences was enabled. Although validation of the identified peptide spectrum matches (PSMs) was conducted using the target decoy PSM validator node, which allows for a false discovery rate (FDR) of 1%, and the phospho RS 3.1 [73] node was introduced in the workflow for phosphorylation site assessment, phosphorylated peptides were also manually checked. Thus, some were accepted as tyrosine phosphorylated even if they would not fit in the FDR threshold of 1% and/or would not have a value for phosphoRS Site Probabilities above 75%. For a peptide to be designated as tyrosine phosphorylated, it was imperative that the b and/or y ions from the phosphorylation site (several fragment ions before, with and after the respective phosphorylated tyrosine) and also other fragment ions that would prove the specific phosphorylation at that residue and not at other eventual serine or threonine residues, should be identified. The presence of the characteristic immonium ion of phosphotyrosine [74,75] was also taken into consideration when designating a peptide as being phosphorylated at a tyrosine residue (only in this experiment, because HCD fragmentation was implemented in the MS analysis). Regarding relative quantification and normalization, since we detected chymotrypsin could cut after F, Y, W, and L, but not after pY, and this generated heterogenous phosphopeptides (different length, charge, even triple-phosphorylated forms), we chose a representative peptide for every phosphotyrosine residue, which had only that residue phosphorylated. Only a single charge of the peptide was chosen for relative quantification. In the cases where peptides contained methionine (same sequence and charge), both oxidized and unoxidized forms were taken into consideration. The oxidized forms manifested the same phosphorylation trend. Relative quantification was used to characterize the evolution of phosphorylation for a certain tyrosine residue. For each technical replicate, the AUC (area under the curve) of peptides corresponding to identified phospho-tyrosine residues were normalized to the AUC of a peptide that could not be phosphorylated in these in vitro reactions and did not contain C or M residues (susceptible to modification). For normalization of EYA3 D311N in vitro phosphorylation, the peptide GVTGQTNSDAESTTL (charge 2+, belonging to EYA3 D311N) was used. For normalization of EYA3 WT in vitro phosphorylation, peptide ENAEGDRTTPSIIAY (charge 2+) from DnaK (DnaK—copurified from E. coli, low amount in sample—is not phosphorylated by Src) was used. The mean value was determined from three technical replicates. Then, for each tyrosine residue, the average values obtained (at 5 min, 30 min, 2 h, and 6 h) were normalized against the highest average value for that certain residue. The values shown in the bar graphs represent these last values obtained (percent from a maximum) with the standard deviation for the three technical replicates also calculated and shown (did not exceed 10.3297) (*n* = 3).

#### 4.6.2. In Vitro Tyrosine Phosphorylation and Dephosphorylation of 6xHis-EYA3 D311N

Purified 6xHis-EYA3 D311N was subjected to a kinase reaction using GST-v-Src. The molar concentration of EYA3 D311N in all reactions was 10 µM. All reactions were stopped with 5× Laemmli sample buffer. The first sample was taken and quenched after 5 min of incubation and the second sample was taken after 90 min of incubation. At this time point, PP2 was added so its concentration would be 1 mM and the reaction was incubated at 30 °C for another hour. The reaction was divided in four equal reactions after this hour. One was stopped with 5× Laemmli sample buffer whereas the other three were treated with purified 6xHis-ED EYA3 WT, in twice the molar concentration of EYA3 D311N, and incubated for 0, 2, and 4 h at 30 °C. The concentration of PP2 in these four reactions became 0.5 mM. The maximum concentration of DMSO (the solvent for PP2) in every reaction was 10%, in order to avoid ED EYA3 WT inactivation. Tyrosine phosphorylation of EYA3 D311N in every sample was verified by Western blot analysis using anti-pY antibody and loading with the anti-His antibody. Two control reactions were performed by incubating EYA3 D311N with and without ATP for 2 h at 30 °C in a kinase assay buffer. Two independent experiments were performed. Within in-gel digestion, samples were migrated through SDS-PAGE and stained with Coomassie Brilliant Blue. Bands containing EYA3 D311N from each time point were subjected to an in-gel digestion protocol with 10 ng/µL sequencing grade chymotrypsin, at 25 °C for 16 h, as previously described [76,77]. In the nLC-MS/MS analysis, the peptides were resuspended in solvent A (0.1% FA in 2% MeCN) and then injected into an nLC (EASY nLC II, Thermo Fisher Scientific, Germany) that was coupled online with an LTQ™ - Orbitrap Velos Pro™ mass spectrometer (Thermo Fisher Scientific). First, the peptides were trapped and desalted on a C18 (2 cm × 100 µm) column (Thermo Fisher Scientific), and then separated on an analytical C18 column (10 cm × 75 µm) (Thermo Fisher Scientific) prior to MS/MS analysis. A 40-min gradient of 2% to 30% solvent B (0.1% FA in 98% MeCN) with a flow rate of 300 nL/ min was implemented for the elution of the peptides. The acquisition method is comprised of a precursor ion scan (300–1650 m/z interval, resolution of 30,000 at 400 m/z) followed by a data-dependent analysis of the five most intense peaks from the survey scan (having charges +2, +3, and higher) by CID and ETD. For the MS1 survey scan, the lock mass option was set to ON and dynamic exclusion had the following settings: repeat count = 1, repeat duration = 30 s, exclusion list size = 500, exclusion duration = 30, and exclusion relative to reference mass = 10 ppm. The mass spectra of the precursor ions were acquired in the profile mode, in the Orbitrap, whereas the fragment ions spectra were obtained in centroid mode, in the linear ion trap. Data analysis and interpretation were performed as described in the previous experiment, with the following modifications: the sequences for 6xHis-EYA3 WT and D311N without the start methionine, Skp without the first 20 amino acids and DnaK, without the start methionine were introduced in the Human database from UniProtKB/ Swiss-Prot, the fragment mass tolerance was set to 0.6 Da, and a maximum of six missed cleavages were set for chymotrypsin (full). For each technical replicate, a normalization of the AUC of peptides corresponding to each phosphorylated tyrosine residue identified was achieved against the AUC of a peptide from EYA3, GVTGQTNSDAESTTL (charge 2+). This peptide does not contain any tyrosine residues so it could not be phosphorylated by Src kinase in the in vitro experiment described and does not contain C and M, which are residues susceptible to modification. The values resulted after normalization of the two technical replicates were averaged. Then, for each residue, the average values obtained at every time point of the experiment were normalized against the highest average value for that certain residue (noted ”percent from a maximum”). The bar chart shows the mean ± SEM corresponding to two independent experiments (each having two technical replicates, *n* = 4).

#### 4.6.3. Detection of Phosphorylated Residues of Transiently Expressed EYA3

HEK293T cells were passaged 24 h before transfection. In the case where only a pCS2-MT vector encoding for EYA3 WT, EYA3 D309N, or EYA3 D309N Y77F was transfected, 22 µg of DNA were used. When pCS2-MT EYA3 vector was co-transfected with the pSLX-c-Src Y527F vector, a ratio of 1:1 between the two vectors was used, with 18 µg total DNA quantity. The cells were grown for 40 h, (changing the media 24 h post-transfection) and then harvested and lysed. The total protein concentration of the soluble protein extract was measured. The expression for each of the proteins of interest (c-myc-EYA3, c-Src Y527F) and for the loading control (GAPDH) was determined by Western blot using anti-c-Myc, anti-v-Src, and anti-GAPDH antibodies. IP of c-myc tagged EYA3 protein was performed using an anti-c-Myc (9E10, sc-40) antibody, as previously described. The resulting samples were first analysed by Western blot using anti-c-Myc and anti-pY antibodies. In-gel digestion was performed as described earlier. The phosphopeptide enrichment for every peptide sample was performed using Titansphere™ Phos-TiO Kit with 1 mg of TiO_2_/ 10 µL tip (GL Sciences, Tokyo, Japan), according to the manufacturer’s specifications (lactic acid to inhibit non-specific adsorbtion of peptides and elution made first with aqueous ammonia solution followed by pyrrolidine solution) [78,79]. The enriched phosphopeptides were further cleaned-up and desalted using reversed solid phase extraction materials such as styrene divinylbenzene (GL-Tip SDB, GL Sciences) and graphite carbon (GL-Tip GC, GL Sciences) using the protocol described by the manufacturer. The eluates were dried in a vacuum concentrator and stored at −20 °C. nLC-MS/MS analysis was performed as previously described (Section 4.6.2 In vitro tyrosine phosphorylation and dephosphorylation of 6xHis-EYA3 D311N). Two technical replicates were performed from each sample. Data analysis and interpretation were performed as described in the first experiment, with some observations. Regarding ETD, the Non-fragment Filter processing node was introduced in the workflow, having set the removal of precursor peaks within a 4 Da mass window. The removal of charge reduced precursors within a 2 Da mass window and the removal of neutral losses reduced charge-reduced species within a 2 Da mass window (maximum neutral loss mass set to 130 Da). The peaks generated from the two activation types (CID and ETD) were searched using Sequest HT, against the UniProtKB/ Swiss-Prot database in which the sequences for several c-Myc tagged EYA3 proteins (WT, D309N, D309N Y77F) were introduced. The searches were performed with a set of three missed cleavages for chymotrypsin (full) and the fragment mass tolerance at 0.6 Da.

### 4.7. In Vitro PTP Assay

HEK293T cells were transiently transfected with vectors encoding for myc-tagged EYA3-WT, D309N, WT T2, and WT T9. Cells were harvested 24 h post-transfection and then lysed. The total protein concentration of soluble extract was measured. Myc-tagged EYA3 as well as anti-actin Western blot experiments verified EYA3 expression in the lysates. EYA3 IP followed, by incubating the soluble protein extract with anti-c-Myc antibody for 1 h at 4 °C and then adding 10 µL of Protein G Sepharose^®^ and overnight incubation on a rotator at 4 °C. The next day, the PTP assay was performed, according to the protocol described by Lorenz U [80]. Beads were washed (10 min, rotator, 4 °C) three times with 20 mM HEPES pH 7 buffer containing 10 mM MgCl_2_ and two times with phosphatase assay buffer (20 mM MES pH 6.5, 100 mM NaCl, 10 mM MgCl_2_, and 5 mM DTT). Each sample was incubated with 10 mM pNPP in a final volume of 100 µL phosphatase assay buffer. After 30 min at 37 °C with mixing, 500 µL of 3N NaOH were added to stop the reactions and the samples were kept for 5 min at RT and then centrifuged 1 min at 13,000 rpm. The absorbance of the supernatant was measured in triplicate, at 405 nm, in a 96-well microplate using FLUOstar Omega (BMG Labtech, Ortenberg, Germany) microplate reader.

### 4.8. Kinetics of EYA3 WT

First, the bacterially expressed and purified 6xHis-EYA3 WT was incubated either alone, with ATP, with Src kinase, or with ATP and Src, for 2 h at 37 °C. All reactions were performed in a buffer containing 20 mM MES pH 6.5, 100 mM NaCl, 10 mM MgCl_2_, and 5 mM DTT. In the kinase reactions, the ratio between EYA3 and GST-v-Src was 25:1 (*w/w*) (4 µg of 6xHis-EYA3 WT and 0.16 µg GST-v-Src). ATP concentration was 100 µM. The total reaction volume was set at 100 µL.

Next, for the study of Src-phosphorylated or unphosphorylated EYA3 WT activity, pNPP was used as a substrate. Briefly, 20 µL from the previous reaction were incubated with 10 mM pNPP in a final volume of 100 µL. The same buffer as in the first reaction was used. The product formed, para-nitrophenol, was measured at 405 nm for 1 h at 37 °C using a spectrophotometer (FLUOstar Omega Microplate reader, BMG Labtech, Germany). Three technical replicates were made (*n* = 3). 

### 4.9. MTS Assay

The proliferation assay was performed using a colorimetric method, the 3-(4,5-dimethylthiazol-2-yl)-5-(3-carboxymethoxyphenyl)-2-(4-sulfophenyl)-2H-tetrazolium, inner salt (MTS) reagent, and an electron coupling reagent, phenazine ethosulfate—PES (CellTiter 96^®^ AQ_ueous_ One Solution Cell Proliferation Assay). The cells (in six well plates) were transiently transfected with the pCS2-MT empty vector, pCS2-MT EYA3 WT, or pCS2-MT EYA3 WT T2 either alone (3 µg of DNA/ well) or with pSLX c-Src Y527F vector—the DNA ratio between pCS2-MT (EYA3) and pSLX c-Src Y527F was 5:1 (2 µg:0.4 µg). After 24 h, cells were detached, counted, and plated at 10,000 cells/ 100 µL cell growth media, in a 96-well plate. The plate was incubated for 48 h at 37 °C in a humidified, 5% CO_2_ atmosphere. The rest of the cells were analysed by Western blot in order to verify that the proteins of interest (myc-tagged EYA3, c-Src Y527F) were expressed. After the incubation period, 20 µL of CellTiter 96^®^ AQ_ueous_ One Solution Reagent were added and the plate was incubated in the same conditions for up to 4 h. The absorbance at 490 nm was measured in a 96-well plate reader—FLUOstar Omega, BMG Labtech—with the following absorbance spectrometer-based technical specifications: OD range of 0 to 4 OD, accuracy <1% at 2 OD, precision <0.5% at 1 OD, and <0.8% at 2 OD. The values introduced in the calculations were obtained after 2 h of incubation with the MTS reagent. The coefficient of determination (*R*^2^) was verified 48 h after plating from 2500 up to 12,500 cells/well. Even after 2 h and 30 min of incubation with the MTS reagent, the *R*^2^ value was 0.9912.

### 4.10. Cell Cycle Analysis

HEK293T were transfected either with an empty vector (pCS2-MT), a vector encoding for c-myc tagged EYA3 WT or EYA3 WT T2, alone or with a pSLX c-Src Y527F or pSLX c-Src Y527F K295R vector. Twenty-four hours post-transfection, cells were harvested with trypsin, counted, and 2 × 10^5^ cells were used for flow cytometry, with the rest being lysed for Western blot analysis using anti-c-myc, anti-v-Src, and anti-GAPDH antibodies. For flow cytometry, cells were washed with 1 mL of FACS buffer (2% FBS in PBS) and fixed in cold absolute ethanol while vortexing. Cells were stored at −20 °C until staining. Before analysis, they were washed two times with PBS and then stained with propidium iodide (0.05 µg/µL) in a solution containing RNAse A (0.5 ng/µL) and 38 mM citrate buffer. Cells were kept for 30 min at RT in the dark before acquisition on the FACSVerse™ instrument (BD Biosciences, USA). The gating strategy excluded cell debris and doublets. The G0/G1, S, and G2/M phases of the cell cycle were manually assessed. The gating strategy and data processing were performed using the FACSuite software (BD Biosciences).

### 4.11. CFSE Assay 

The cultured HEK293T cells were harvested with trypsin, counted, then centrifuged at 300× *g* for 5 min at 25 °C, and washed once with 0.1% BSA in PBS. After resuspending them in prewarmed 0.1% BSA in PBS, approximatively 2 × 10^5^ cells were taken to be measured at FACSVerse™ instrument (BD Biosciences) as a negative control for the CFDA SE (CFSE) staining. The rest of the cells were subjected to staining with 10 µM CFDA SE (Invitrogen), according to the manufacturer recommendations. Lastly, cells were resuspended in prewarmed complete growth media and plated in six well plates, in order to reach approximately 70% confluency. An aliquot was immediately acquired as day 0 reference. After 4 h, cells were transfected either with an empty vector (pCS2-MT), a vector encoding for c-myc tagged EYA3 WT or EYA3 WT T2, alone or with pSLX c-Src Y527F or the pSLX c-Src Y527F K295R vector. Forty-eight hours post-transfection cells were harvested with trypsin, centrifuged at 300× *g* for 5 min, at 4 °C, washed with 1 mL of FACS buffer (2% FBS in PBS), and then resuspended in 1 mL of the same type of buffer, to be measured on the flow cytometer. Gating strategy excluded cell debris and doublets. CFSE dilution in the proliferating cell pool was assessed as a decrease in median fluorescence intensity (MFI). The remaining cells were lysed for Western blot analysis using anti-c-myc, anti-v-Src, and anti-GAPDH antibodies. FACS analysis was performed using the FACSuite software (BD Biosciences). 

### 4.12. Bioinformatic Sequence Analysis

The variability of tyrosine residues in EYA3 homologues was assessed starting from a set of 721 homologues identified in Reference Proteomes database RP55 [70] using the Jackhmmer algorithm from the HMMER webserver [81]. A number of three iterations were enough for the search to converge at a 10^−4^ e-value threshold using a BLOSUM62 matrix, while the rest of the parameters were kept at the default values. 

The set of 721 homologous proteins identified were realigned with MAFFT [82] using Unipro UGENE v1.22.0 suite [83], with the default and recommended parameters. A sequence conservation logo of the relative entropy of each amino acid position in the alignment was computed using the Schneider and Stephens approach of defining sequence variability [84] as implemented in the WebLogo suite [85].

### 4.13. Mass Spectrometry Data Deposition

The mass spectrometry proteomics data have been deposited to the ProteomeXchange Consortium via the PRIDE [86] partner repository with the dataset identifiers PXD016518, PXD016520, PXD016526, PXD016532, and PXD016596.

## Figures and Tables

**Table 1:** The EYA3 phosphorylation pattern in HEK293T cells.

Experiment	Phosphoresidues Detected										
	Tyrosine							Serine			
	72	77	90	105	108	208	325	138	157	225	438
EYA3 WT								X		X	X
EYA3 D309N								X		X	X
EYA3 D309N Y77F								X		X	X
EYA3 WT + c-Src Y527F		X			X	X		X	X	X	X
EYA3 D309N + c-Src Y527F	X	X	X	X	X	X	X	X	X	X	X
EYA3 D309N Y77F + c-Src Y527F	X		X	X	X	X	X	X	X	X	X

Phosphorylated tyrosine and serine residues of EYA3 (identified by nano-high performance liquid chromatography with tandem mass spectrometry (nLC-MS/MS) analysis after enrichment of phosphopeptides using TiO_2_) corresponding to myc-tagged EYA3 proteins immunoprecipitated from HEK293T. Two independent experiments were performed, with two technical replicates for every sample. Symbol “X” indicates detected phosphoresidues of EYA3 immunoprecipitated from HEK293T cells.

## Supplementary Materials

The following are available online at https://www.mdpi.com/1422-0067/20/24/6307/s1.

## Abbreviations

AUC	area under the curve
BB	benzbromarone
CID	collision-induced dissociation
CFSE	carboxyfluorescein succinimidyl ester
DN	EYA3 D309N
ED	Eya Domain
ESI-MS	electrospray ionisation - mass specrometry
ETD	electron-transfer dissociation
FA	formic acid
FDR	false discovery rate
HCD	higher-energy collisional dissociation
IP	immunoprecipitation
m/z	mass/charge
MeCN	acetonitrile
nLC-MS/MS	nano-high performance liquid chromatography - tandem mass spectrometry
PEI	polyethylenimine
pNPP	para-nitrophenylphosphate
PTP	protein tyrosine phosphatase
pY	phosphotyrosine
