# Supplementary material for: Analysis of EYA3 Phosphorylation by Src Kinase Identifies Residues Involved in Cell Proliferation
Source: Int J Mol Sci. 2019 Dec 13;20(24):6307. doi: 10.3390/ijms20246307 (PMC6940942; doi:10.3390/ijms20246307)
Supplement: Supplementary file 1 [file ijms-20-06307-s001.pdf]

Supplementary Materials:

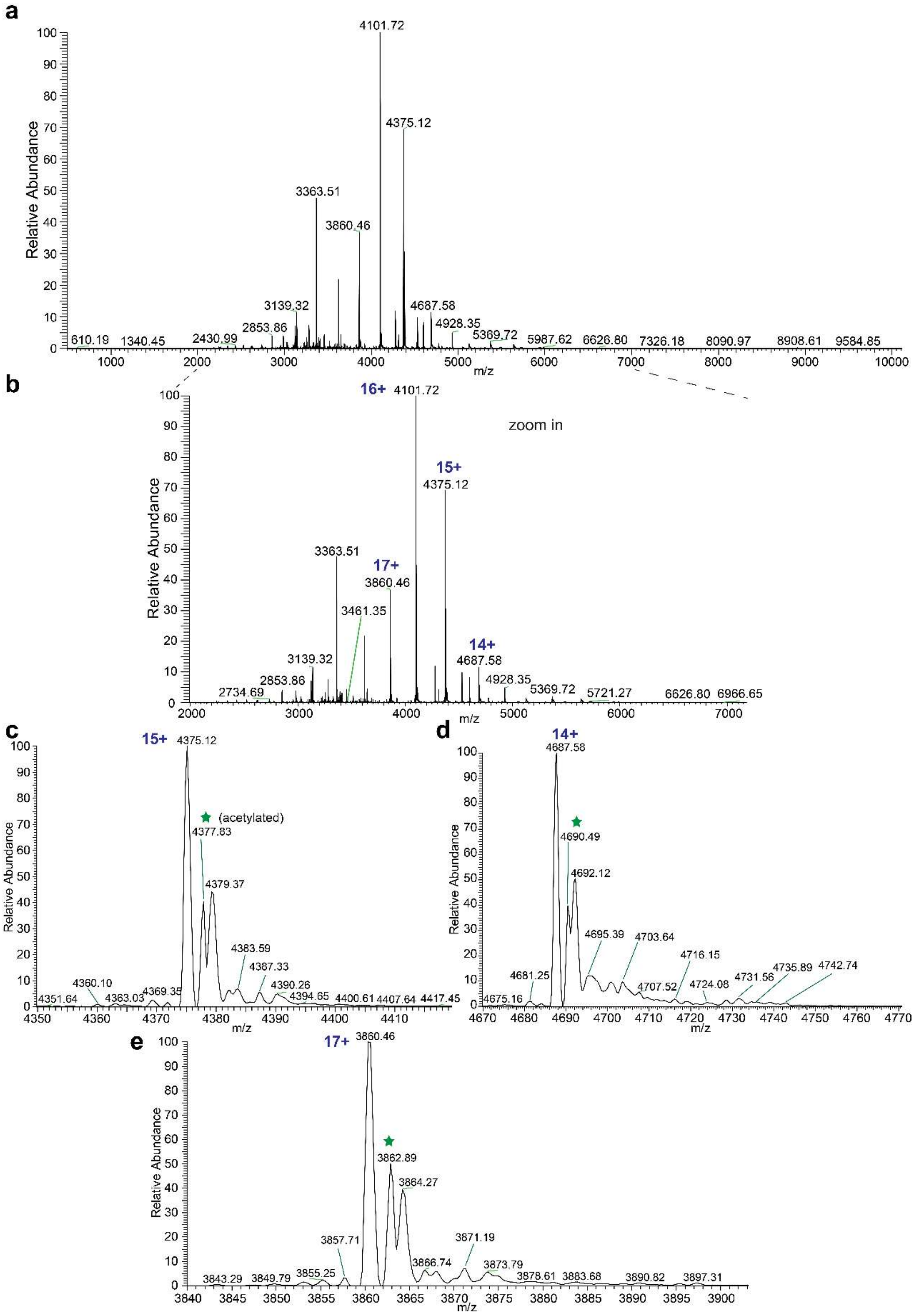

Figure S1. Native MS analysis of N-terminal His-tagged EYA3 WT. (**a**) Native mass spectrum of purified 6xHis-EYA3 WT. (**b**) Zoom in on the native mass spectrum, evidencing 6xHis-EYA3 WT charge state envelope. (**c**), (**d**) and (**e**) Zoom in on charges 15+, 14+ and 17+ corresponding to N-terminal His-tagged EYA3 WT.

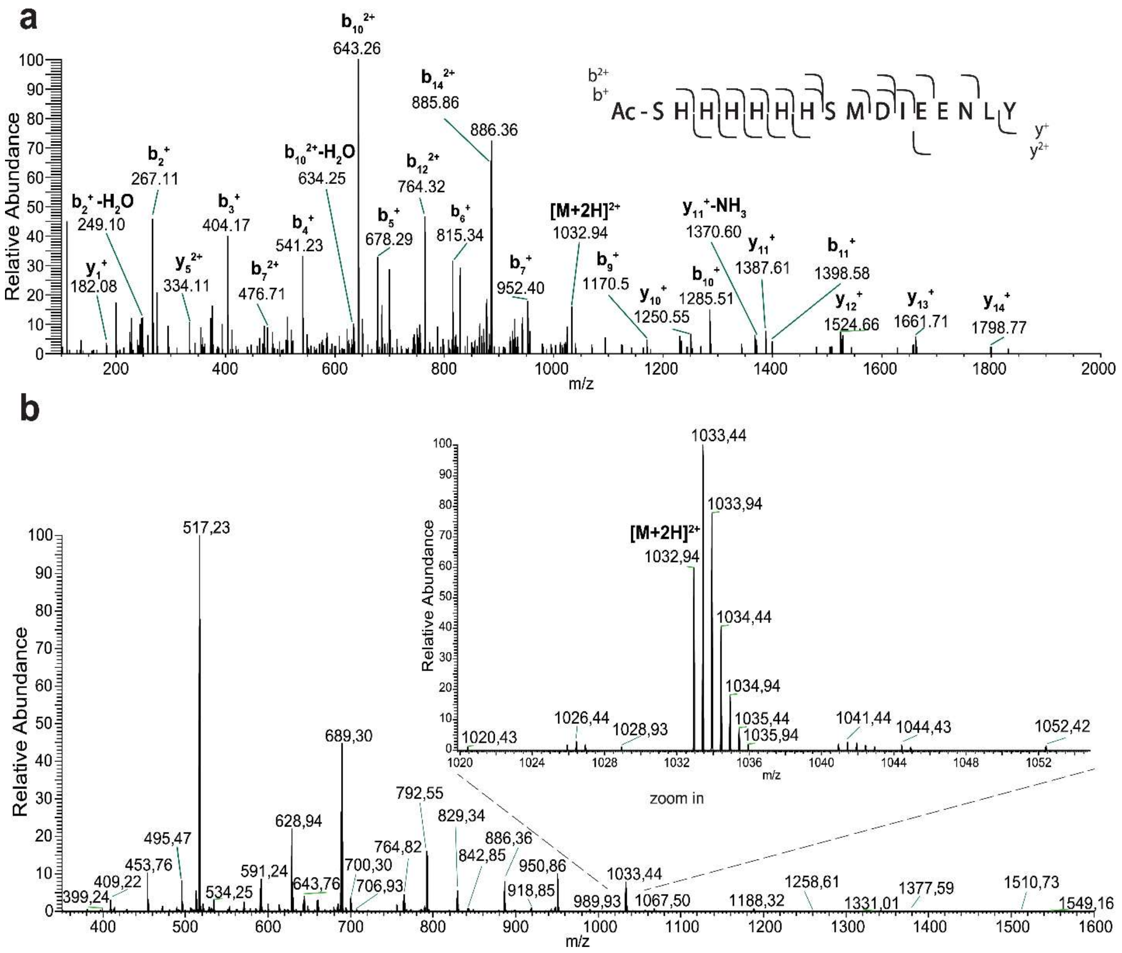

Figure S2. Evidence of Met removal and Ser1 acetylation in 6xHis-EYA3 proteins. The hypothesis of post-translational modification of the purified protein tyrosine phosphatases was searched through bottom-up mass spectrometry, after in-solution digestion of each of the two EYA3 proteins (WT and D311N) using sequencing grade chymotrypsin. Peptides were analysed on an Orbitrap Elite™ Hybrid Ion Trap - Orbitrap™ mass spectrometer coupled to a Proxeon EASY - nLC 1000 (Thermo Fisher Scientific) using HCD as fragmentation method. Raw data were searched with Proteome Discoverer v1.4 using Sequest HT algorithm with the following settings: precursor mass tolerance of 10 ppm, fragment mass tolerance of 0.05 Da, chymotrypsin (full) as enzyme with a maximum of five missed cleavages, methionine oxidation (+15.995 Da) and acetylation (+42,01057 Da) at serine and lysine as dynamic modifications, and also carbamidomethylation (+57.021 Da) on cysteine as static modification. The introduced database was the Human database from UniProtKB/ Swiss-Prot in which the sequences for 6xHis-EYA3 WT and 6xHis-EYA3 D311N without the first methionine were inserted. The peptides designated as acetylated were manually checked. Representative peptide SHHHHHHSMDIEENLY, S1-Acetyl (42.0 Da), charge 2+ (shown here from spectra belonging to 6xHis-EYA3 WT), is evidence that Ser1 - resulted because the first methionine was removed from the sequences of 6xHis-EYA3 WT and 6xHis-EYA3 D311N during prokaryotic expression - is acetylated: (**a**) Fragment mass spectrum with schematic representation of the peptide sequence and the identified fragment ions (b and y, also with neutral losses - H_2O or NH_3 - and even the precursor ion - $[M+2H]^{2+}$). (**b**) Precursor ion scan which includes the peptide ion of this representative peptide (monoisotopic m/z = 1032.94214, z = 2+; isolation = 1033.44 Da/ 1.50 Da) with zoom in on its isotope pattern.

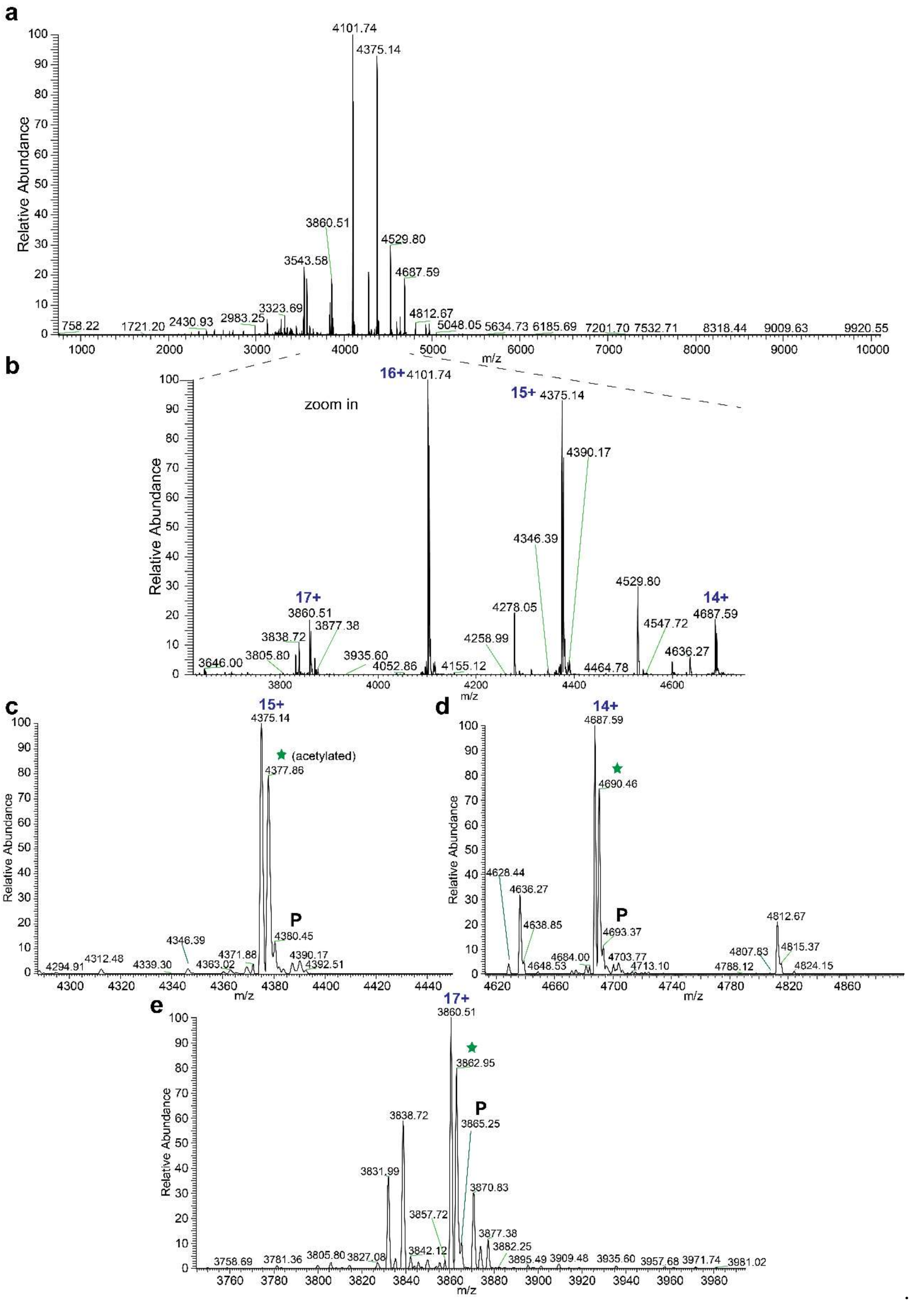

Figure S3. Native mass spectrum of 6xHis-EYA3 WT after 2 h in vitro tyrosine phosphorylation with v-Src. (**a**) Native mass spectrum of sample resulted after 2 h incubation of 6xHis-EYA3 WT with v-Src kinase and ATP. (**b**) Zoom in on the native mass spectrum, evidencing 6xHis-EYA3 WT charge state envelope. (**c**), (**d**) and (**e**) Zoom in on charges 15+, 14+ and 17+ corresponding to N-terminal His-tagged EYA3 WT.

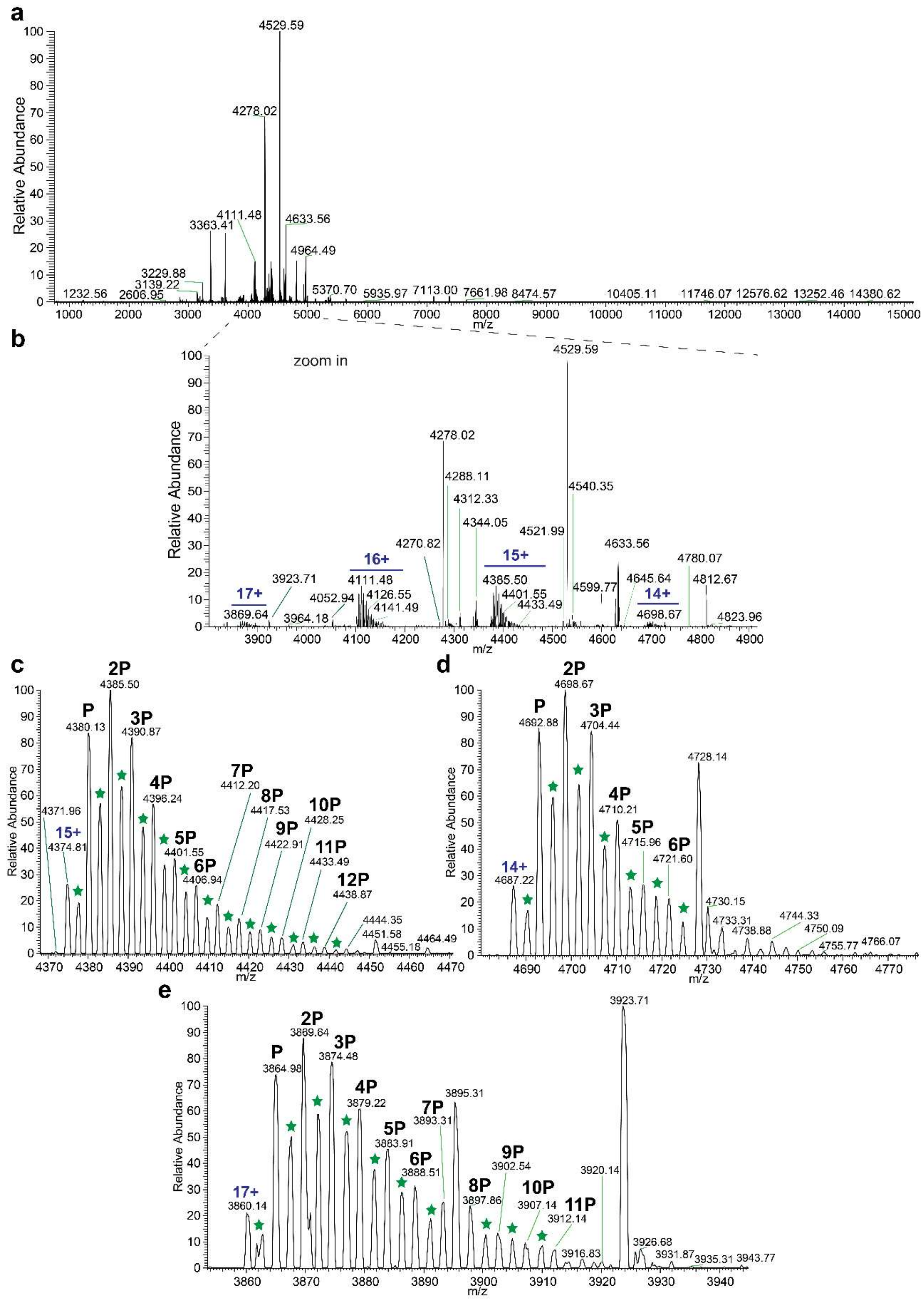

Figure S4. Native mass spectrum of 6xHis-EYA3 D311N after 2 h in vitro tyrosine phosphorylation with v-Src. (**a**) Native mass spectrum of sample resulted after 2 h incubation of 6xHis-EYA3 D311N with v-Src kinase and ATP. (**b**) Zoom in on the native mass spectrum, evidencing 6xHis-EYA3 D311N charge state envelope. (**c**), (**d**) and (**e**) Zoom in on charges 15+, 14+ and 17+ corresponding to N-terminal His-tagged EYA3 D311N.

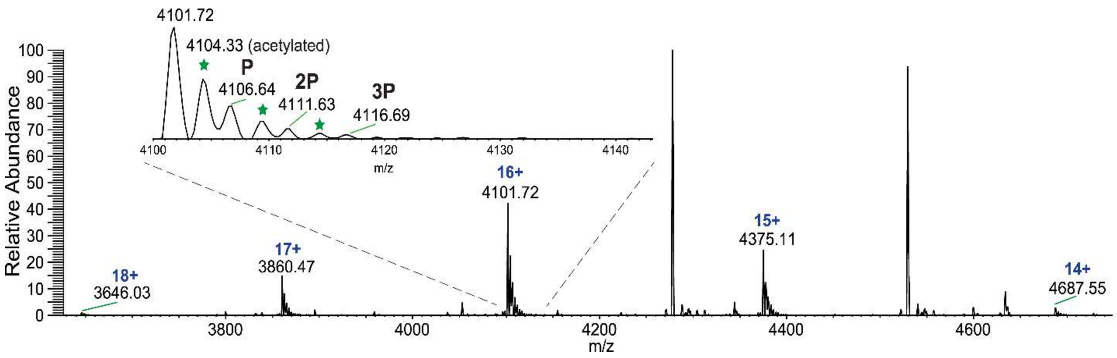

Figure S5. Native mass spectrometry analysis of 6xHis-EYA3 D311N after 5 min of in vitro tyrosine phosphorylation with v-Src. Adjoining peaks of the charge state envelope reveal the presence of modified EYA3 D311N molecules. Zoomed in image on charge 16+ reveals EYA3 D311N molecules carrying up to three phosphate moieties (noted 3P, at m/z = 4116.69).

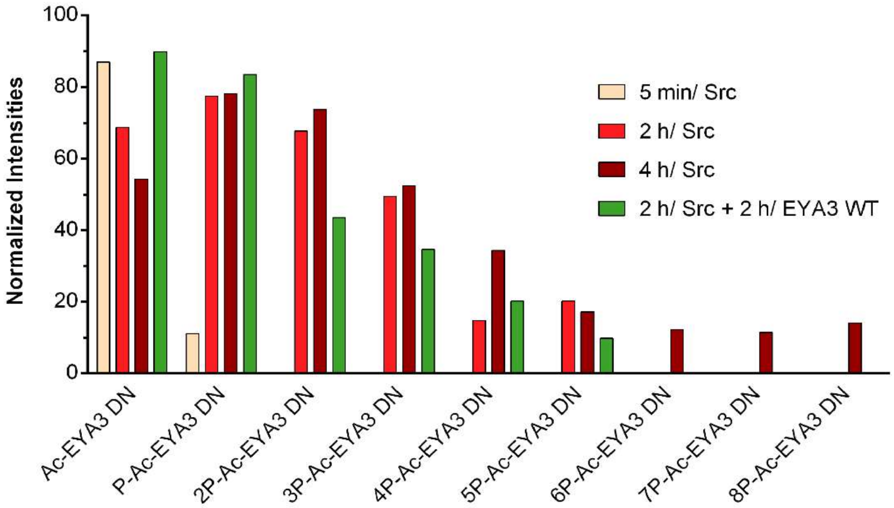

Figure S6. Native MS analysis of the variation of phosphorylation for the acetylated 6xHis-EYA3 D311N molecules. Quantification of the raw data of acetylated 6xHis-EYA3 D311N molecules which carry charge 16+, from the dynamics of EYA3 tyrosine phosphorylation and autodephosphorylation experiment, analyzed by native MS.

Chaperone protein Skp

MKKWLLAAGLGLALATSAQAADKIAIVNMGSLFQQVAQKTGVSNTLENEFKGRASELQRMETDLQAK
MKKLQSMKAGSDRTKLEKDVMAQRQTFAQKAQAFEQDRARRSNEERGKLVTRIQTAVKSVANSQD
IDLVVDANAVAYNSSDVKDITADVLKQVK

Chaperone protein DnaK

MGKIIGIDLGTTNSCVAIMDGTTPRVLENAEGDRTTPSIIAYTQDGETLVGQPAKRQAVTNPQNTLFAI
KRLIGRRFQDEEVQRDVSIMPFKIIAADNGDAWVEVKGQKMAPPQISAEVLKKMKKTAEDYLGEPVT
EAVITVPAYFNDAQRQATKDAGRIAGLEVKRIINEPTAAALAYGLDKGTGNRTIAVYDLGGGTFDISIIEI
DEVDGEKTFEVLATNGDTHLGGEDFDSRLINYLVEEFKKDQGIDLRNDPLAMQRLKEAAEKAKIELS
SAQQTDVNLPYITADATGPKHMNIKVTRAKLESLVEDLVNRSIEPLKVALQDAGLSVSDIDDVILVGGQ
TRMPMVQKKVAEFFGKEPRKDVNPDEAVAIGAAVQGGVLTGDVKDVLLLDVTPLSLGIETMGGVMTT
LIAKNTTIPTKHSQVFSTAEDNQSAVTIHVLQGERKRAADNKSLGQFNLDGINPAPRGMPQIEVTFDID
ADGILHVSAKDKNSGKEQKITIKASSGLNEDEIQKMVRDAEANAEADRKFEELVQTRNQGDHLLHST
RKQVEEAGDKLPADDKTAIESALTALETALKGEDKAAIEAKMQELAQVSQKLMEIAQQQHAQQQTAG
ADASANNAKDDDVVDAEFEEVKDKK

Figure S7. Skp and DnaK co-purify with prokaryotically expressed 6xHis-EYA3 protein. Sequence coverage (from high confidence peptides) of chaperone proteins Skp (Uniprot entry P0AEU7) and DnaK (Uniprot entry P0A6Y8) identified by nLC-MS/MS analysis (made on an LTQ-Orbitrap Elite™ coupled to a Proxeon EASY - nLC 1000, both from Thermo Fisher Scientific, using HCD and ETD as fragmentation methods) of purified 6xHis-EYA3 WT protein sample after in-solution digestion with sequencing grade rLysC and chymotrypsin. Acquired raw data were searched with Sequest HT algorithm in Proteome Discoverer v1.4. The database used for protein identification was *E. coli* from UniProtKB/ Swiss-Prot.

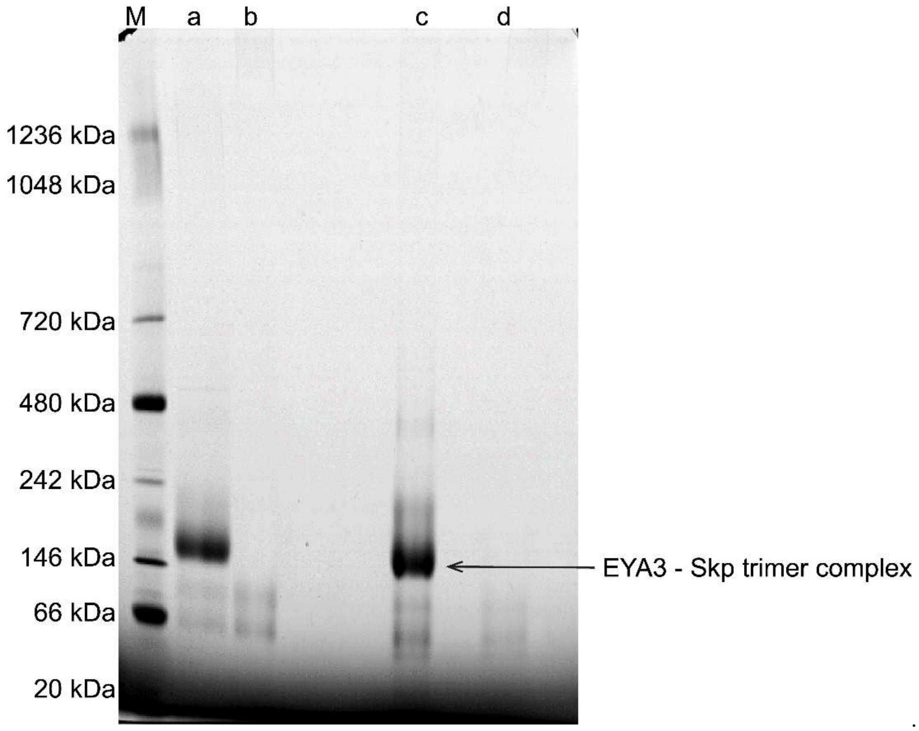

Figure S8. EYA3 - Skp trimer complex dissociates under denaturing conditions. Native protein gel (3-12% Bis-Tris) on purified 6xHis-EYA3 WT and 6xHis-EYA3 D311N samples (quantity: 10 μM in 20 μL/ well): M - NativeMark™ Unstained Protein Standard. (**a**) Native 6xHis-EYA3 WT; (**b**) Denatured 6xHis-EYA3 WT (5 min at 95 °C); (**c**) Native 6xHis-EYA3 D311N; (**d**) Denatured 6xHis-EYA3 D311N (5 min at 95 °C).

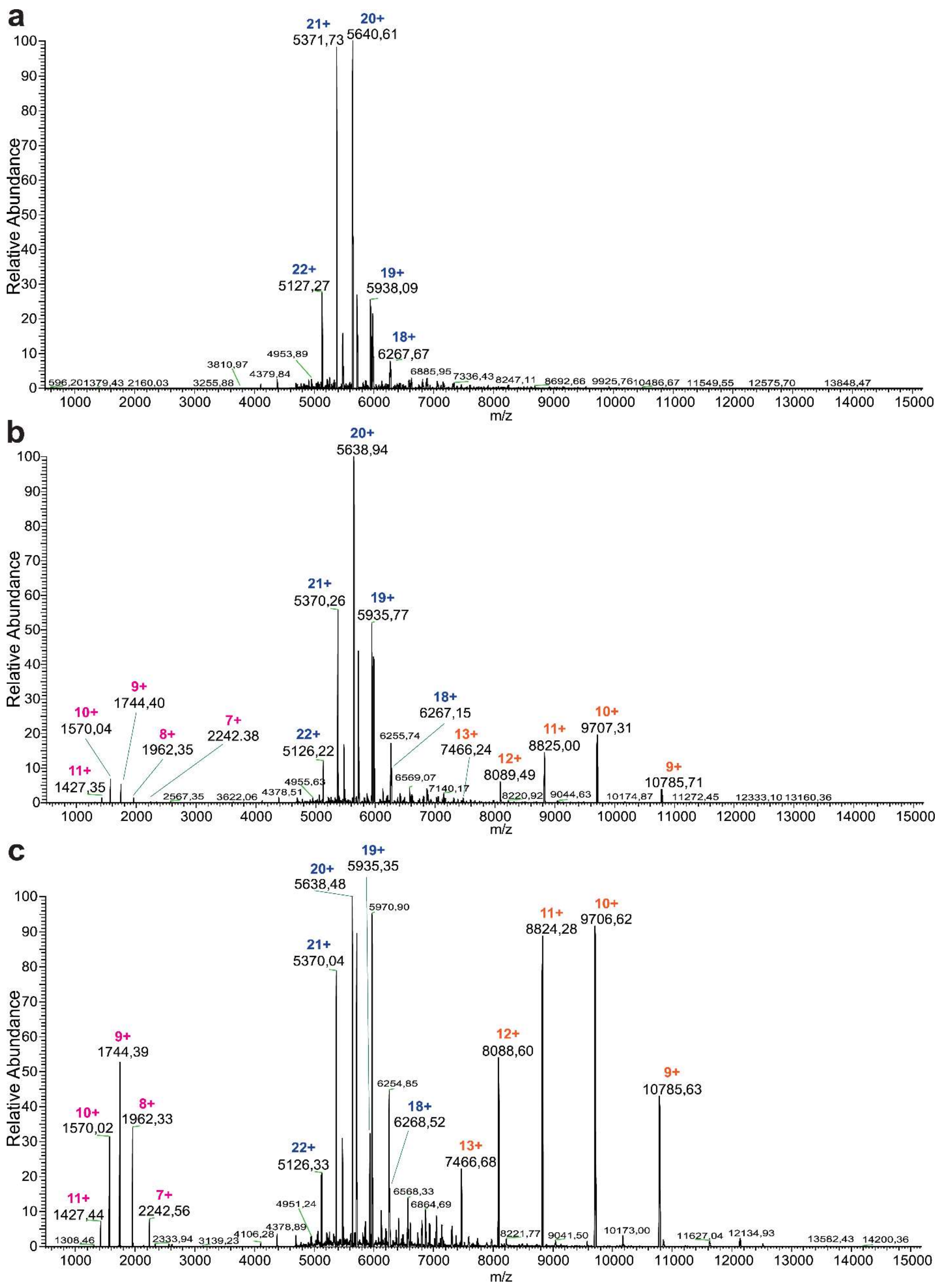

Figure S9. EYA3 - Skp complex in native MS, at increasing energy. An increasing amount of energy is applied to the EYA3 WT - Skp complex, from (**a**) to (**c**). (**a**) EYA3 WT - Skp trimer complex at low energy. The calculated molecular weight (from the peaks of the charge state envelope, charges shown in blue) is 112785.2 ± 7.1 Da and most probably results from the acetylated EYA3 WT (65652.45 ± 0.3 Da, see Figure 1) in complex with Skp trimer (47075.05 ± 0.2 Da, see Figure 2d) and two magnesium ions (Mg^{2+}, atomic weight of 24.305 u). (**b**) When a high energy is applied, the EYA3 WT - Skp trimer complex (also containing a Mg^{2+} ion), having the calculated molecular weight of 112757.2 ± 3 Da, dissociates in the monomer of Skp (charges shown in magenta; with the calculated molecular weight of 15692.62 ± 1.5 Da and having the theoretical molecular weight of 15691.79 Da) and EYA3 WT - Skp dimer complex with Mg^{2+} ion (charges shown in orange; calculated molecular weight of 97059.9 ± 6.6 Da). (**c**) When an even higher energy is applied, the EYA3 - Skp trimer complex with Mg^{2+} ion (112752.3 ± 3.6 Da) dissociated even more into the Skp monomer (calculated molecular weight of 15690.26 ± 0.5 Da) and the EYA3 WT - Skp dimer complex with Mg^{2+} ion (calculated molecular weight of 97055.8 ± 3.9 Da). In both cases, (**b)** and (**c**), the molecular weight of EYA3 WT - Skp dimer complex results from the acetylated EYA3 WT (65652.45 ± 0.3 Da) along with that of Skp trimer (47075.05 ± 0.2 Da) and a Mg^{2+} ion. It can be observed that the proteoform of EYA3 that forms complex with Skp trimer is the acetylated one and that in complex, these molecules retain magnesium ions.

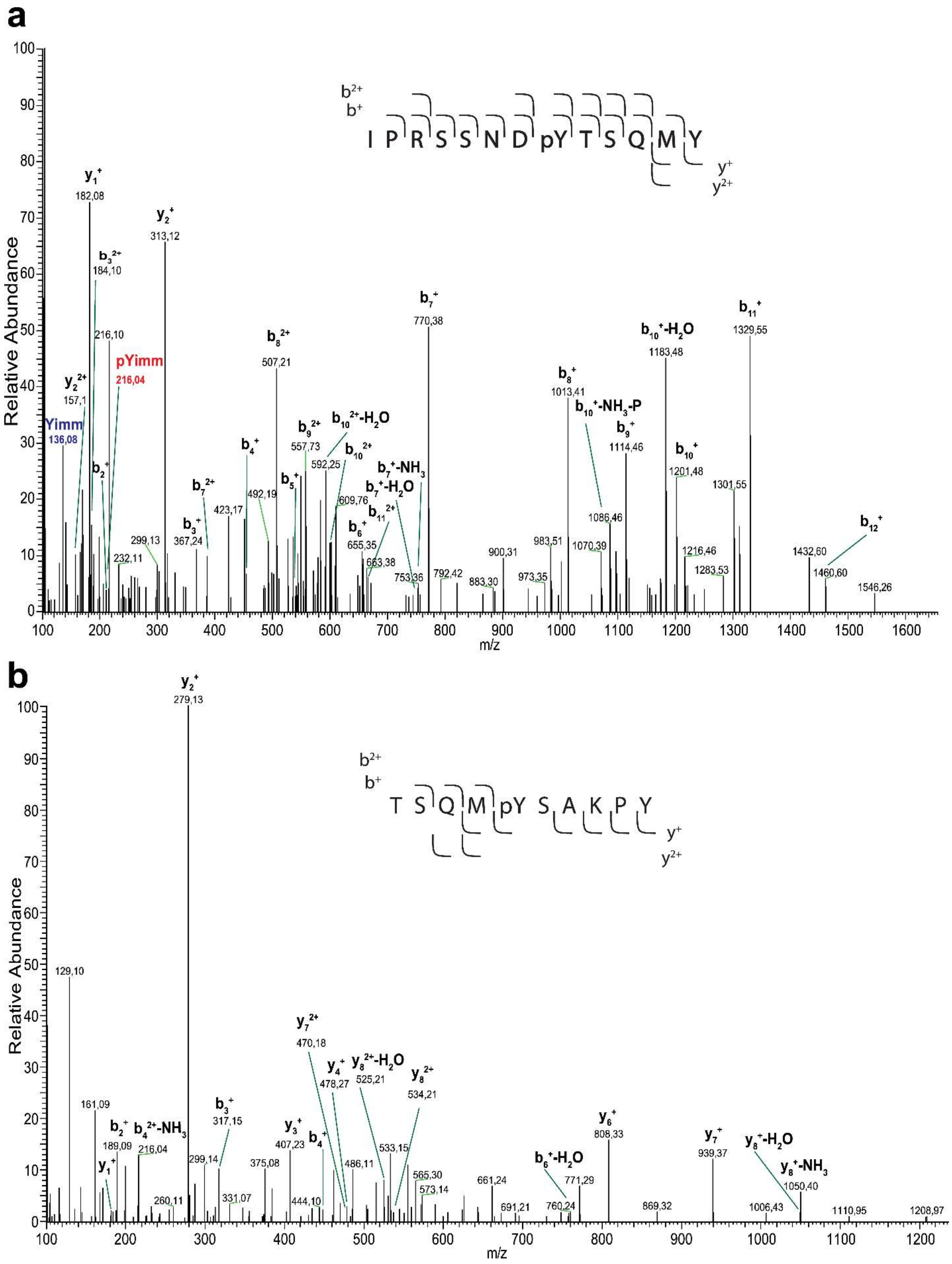
a
b
Relative Abundance
m/z
b2+
b+
I P R S S N D pY T S Q M Y
y+
y2+
T S Q M pY S A K P Y
y1+ 182,08
y2+ 313,12
b32+ 184,10
216,10
pYimm 216,04
Yimm 136,08
y22+ 157,1
b2+
b72+
b3+ 367,24
299,13
232,11
423,17
b4+
492,19
b82+ 507,21
b5+
b92+ 557,73
b102+-H2O
b102+ 592,25
609,76
b6+ 655,35
b112+
663,38
b7+-NH3
b7+-H2O
753,36
b7+ 770,38
792,42
883,30
900,31
973,35
983,51
b8+ 1013,41
b10+-NH3-P
1070,39
1086,46
b9+ 1114,46
b10+-H2O 1183,48
b10+ 1201,48
1216,46
1283,53
1301,55
b11+ 1329,55
1432,60
b12+ 1460,60
1546,26
y2+ 279,13
129,10
161,09
b2+ 189,09
y1+
b42+-NH3 216,04
260,11
299,14
b3+ 317,15
331,07
375,08
y3+ 407,23
444,10
b4+
y72+ 470,18
y4+ 478,27
486,11
y82+-H2O 525,21
533,15
y82+ 534,21
565,30
573,14
661,24
691,21
b6+-H2O 760,24
771,29
y6+ 808,33
869,32
y7+ 939,37
1006,43
y8+-H2O
y8+-NH3 1050,40
1110,95
1208,97

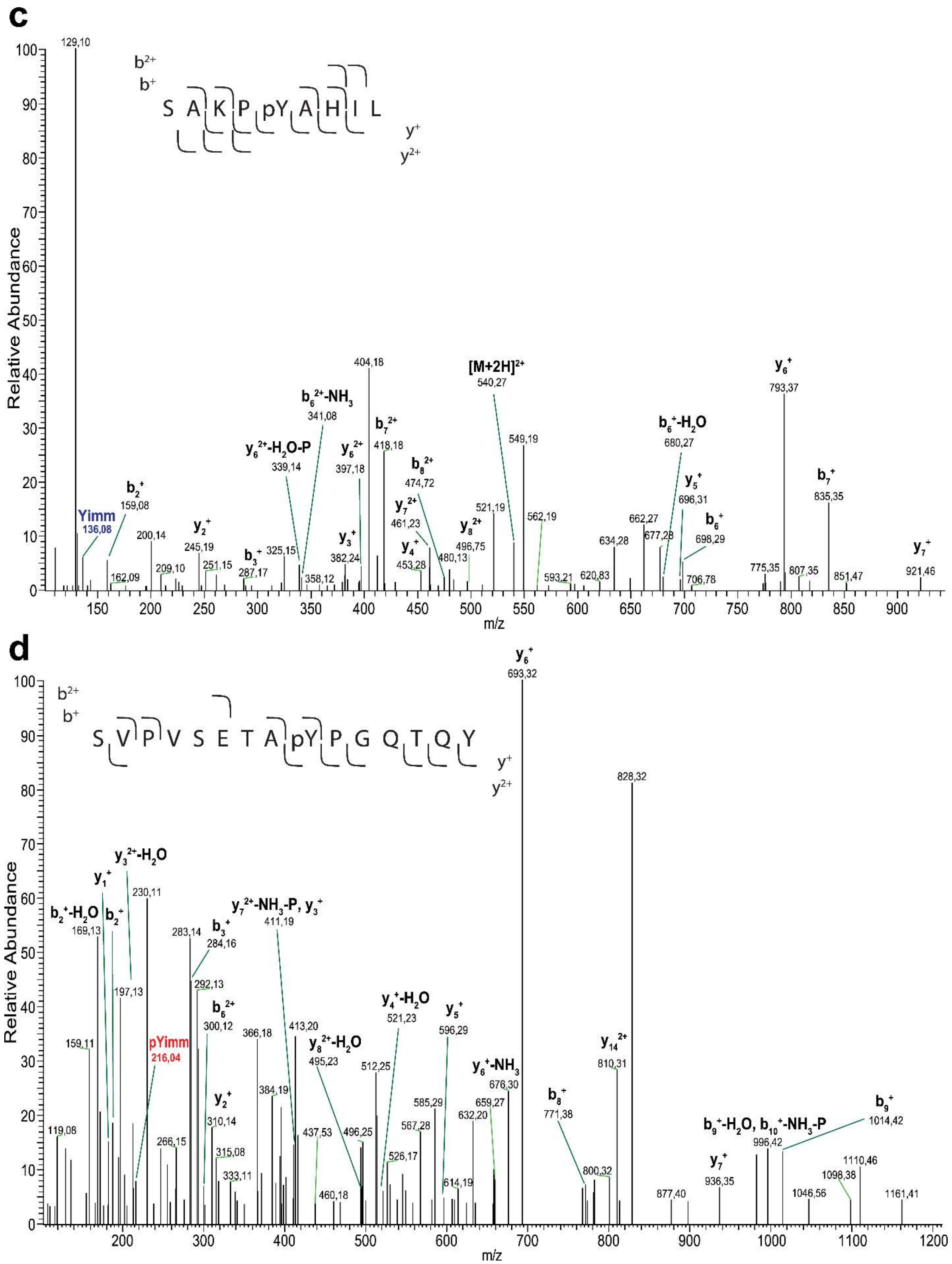
c
SAKPpYAHIL
b2+
b+
y+
y2+
Relative Abundance
m/z
129,10
Yimm 136,08
404,18
[M+2H]2+ 540,27
793,37
835,35
921,46
d
SVPVSETApYPGQTQY
b2+
b+
y+
y2+
Relative Abundance
m/z
693,32
828,32
pYimm 216,04
230,11
1014,42
1161,41

e

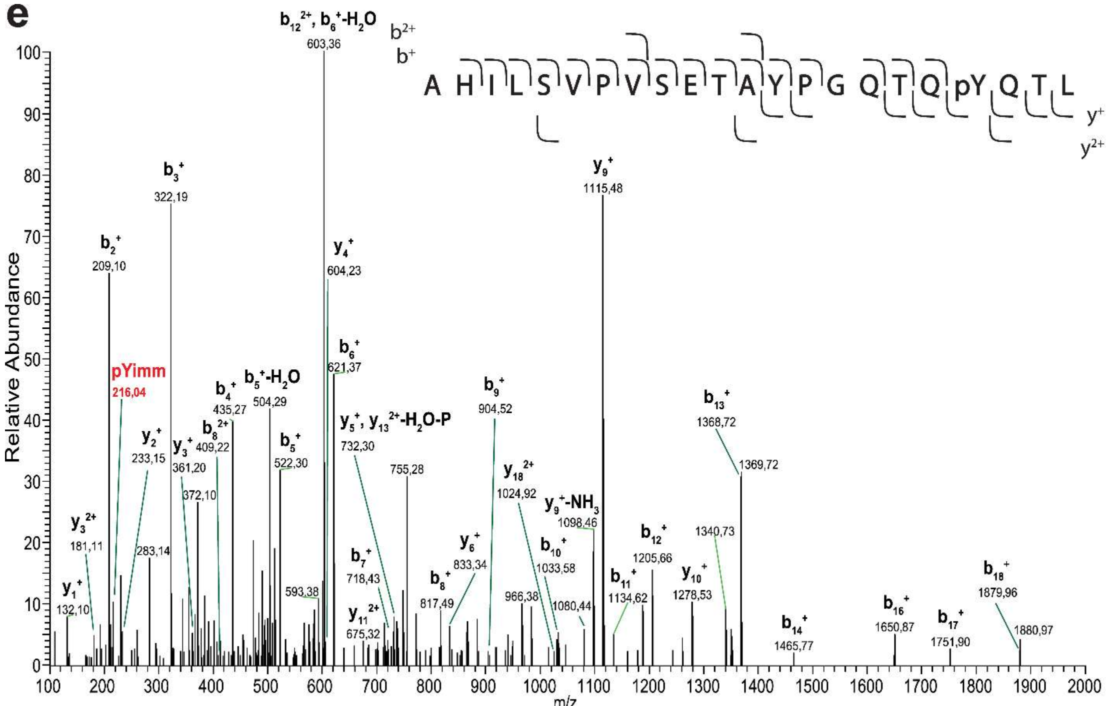
A H I L S V P V S E T A Y P G Q T Q pY Q T L
b2+
b+
y+
y2+
Relative Abundance
m/z
b12 2+, b6+-H2O
603,36
b3+
322,19
b2+
209,10
pYimm
216,04
y9+
1115,48
y4+
604,23
b6+
621,37
b4+
435,27
b5+-H2O
504,29
b8 2+
409,22
y3+
361,20
y2+
233,15
b5+
522,30
y5+, y13 2+-H2O-P
732,30
755,28
b9+
904,52
b13+
1368,72
1369,72
y18 2+
1024,92
y9+-NH3
1098,46
372,10
y3 2+
181,11
283,14
b7+
718,43
y6+
833,34
b10+
1033,58
b12+
1205,66
1340,73
y1+
132,10
593,38
y11 2+
675,32
b8+
817,49
966,38
1080,44
b11+
1134,62
y10+
1278,53
b14+
1465,77
b16+
1650,87
b17+
1751,90
b18+
1879,96
1880,97

f

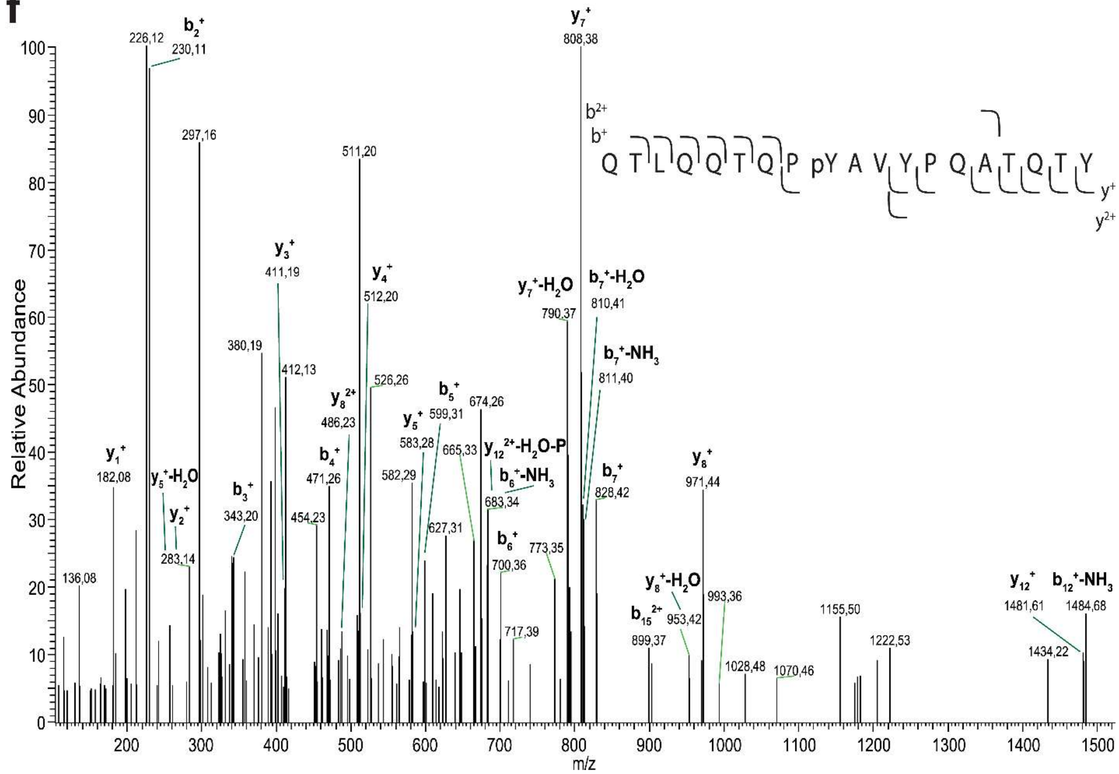
Q T L Q Q T Q P pY A V Y P Q A T Q T Y
b2+
b+
y+
y2+
Relative Abundance
m/z
226,12
b2+
230,11
y7+
808,38
297,16
511,20
y3+
411,19
y4+
512,20
y7+-H2O
790,37
b7+-H2O
810,41
380,19
412,13
b7+-NH3
811,40
526,26
y8 2+
486,23
b5+
599,31
y5+
583,28
674,26
y1+
182,08
y5+-H2O
b4+
471,26
y12 2+-H2O-P
665,33
b6+-NH3
582,29
b7+
828,42
y8+
971,44
b3+
343,20
454,23
683,34
y2+
283,14
627,31
b6+
700,36
773,35
136,08
y8+-H2O
b15 2+
953,42
899,37
993,36
717,39
1155,50
1222,53
y12+
1481,61
b12+-NH3
1484,68
1028,48
1070,46
1434,22

g

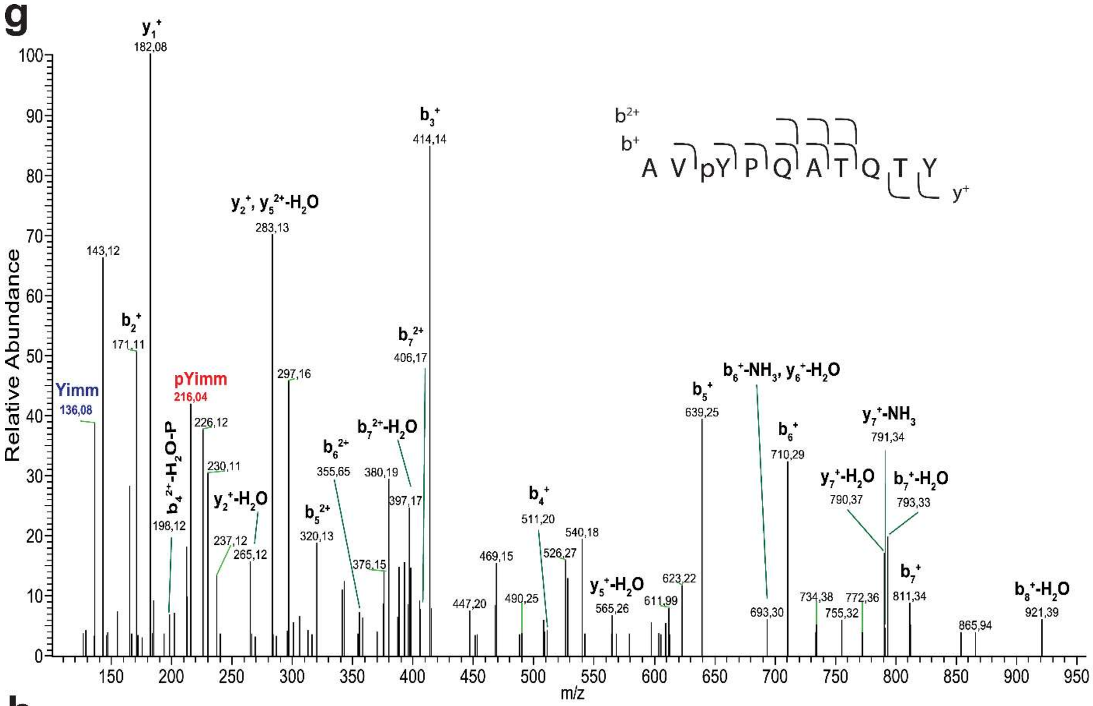

Relative Abundance
m/z
y1+ 182,08
143,12
b2+ 171,11
Yimm 136,08
pYimm 216,04
b4 2+-H2O-P 198,12
226,12
230,11
y2+-H2O 237,12 265,12
y2+, y5 2+-H2O 283,13
297,16
b5 2+ 320,13
b6 2+ 355,65
376,15
b7 2+-H2O 380,19 397,17
b7 2+ 406,17
b3+ 414,14
447,20
469,15
490,25
b4+ 511,20
526,27
540,18
y5+-H2O 565,26
611,99
623,22
b5+ 639,25
b6+-NH3, y6+-H2O 693,30
b6+ 710,29
734,38
755,32
772,36
y7+-H2O 790,37
y7+-NH3 791,34
b7+-H2O 793,33
b7+ 811,34
865,94
b8+-H2O 921,39
b2+ b+ A V pY P Q A T Q T Y y+

h

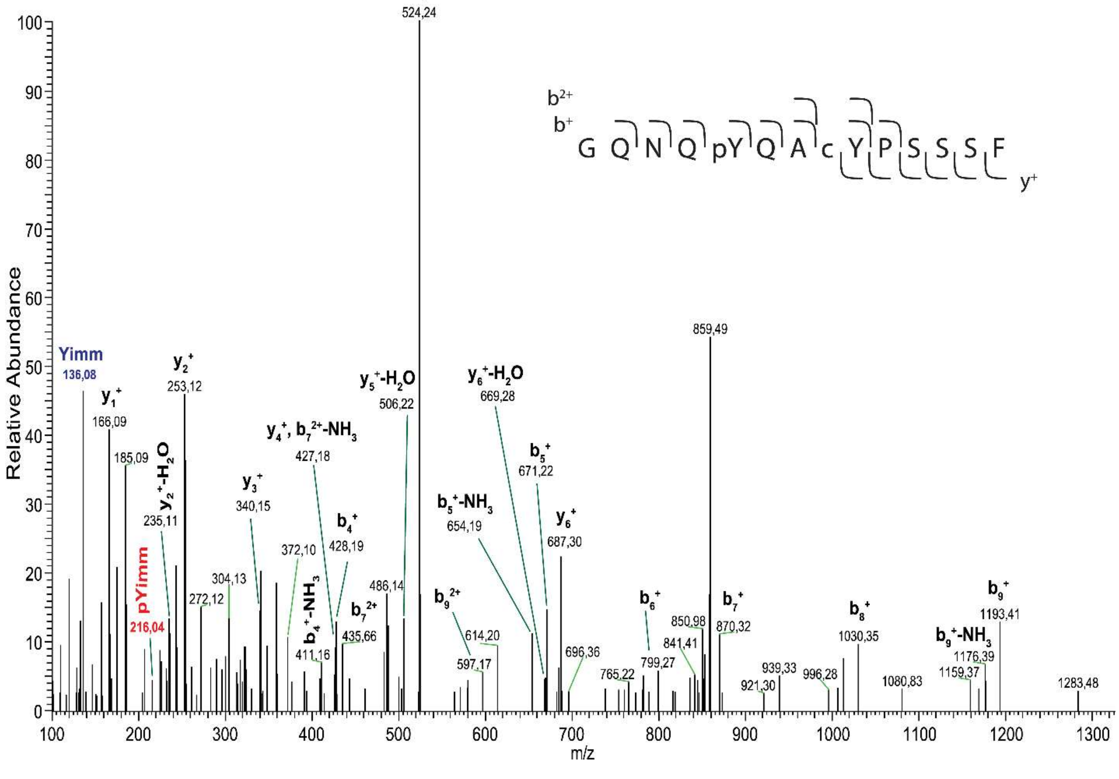

Relative Abundance
m/z
Yimm 136,08
y1+ 166,09
185,09
pYimm 216,04
y2+-H2O 235,11
y2+ 253,12
272,12
304,13
y3+ 340,15
372,10
b4+-NH3 411,16
y4+, b7 2+-NH3 427,18
b4+ 428,19
b7 2+ 435,66
486,14
y5+-H2O 506,22
y5+ 524,24
b9 2+ 597,17
614,20
b5+-NH3 654,19
y6+-H2O 669,28
b5+ 671,22
y6+ 687,30
696,36
765,22
b6+ 799,27
841,41
850,98
859,49
b7+ 870,32
921,30
939,33
996,28
b8+ 1030,35
1080,83
b9+-NH3 1159,37
1176,39
b9+ 1193,41
1283,48
b2+ b+ G Q N Q pY Q A c Y P S S S F y+

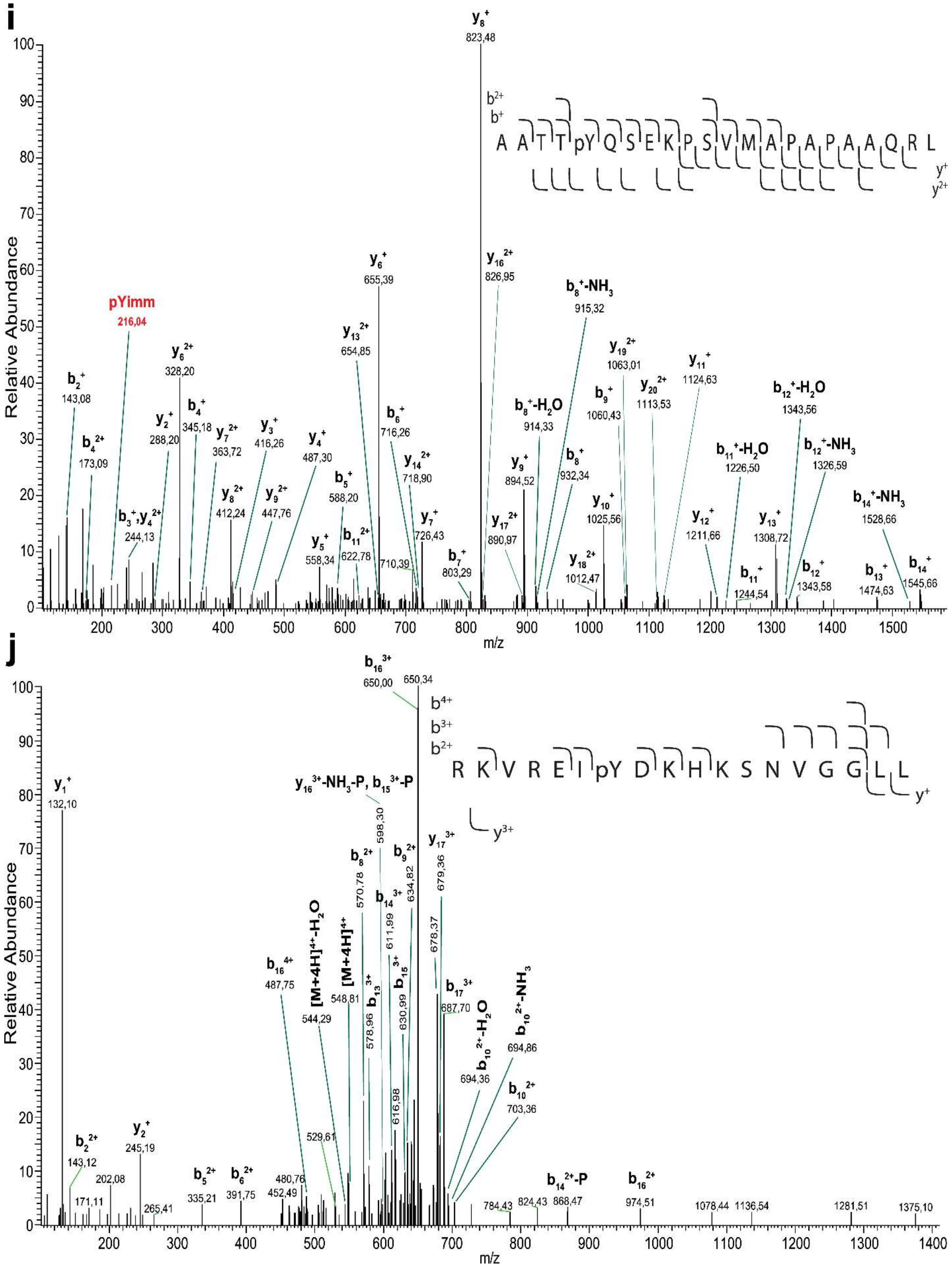
i
Relative Abundance
m/z
A A T T pY Q S E K P S V M A P A P A A Q R L
b2+
b+
y+
y2+
pYimm
216,04
y8+
823,48
y16 2+
826,95
b8+-NH3
915,32
y6+
655,39
y13 2+
654,85
y6 2+
328,20
b2+
143,08
b4 2+
173,09
y2+
288,20
b4+
345,18
y7 2+
363,72
y3+
416,26
y4+
487,30
b6+
716,26
y14 2+
718,90
b3+,y4 2+
244,13
y8 2+
412,24
y9 2+
447,76
b5+
588,20
y5+
558,34
b11 2+
622,78
710,39
y7+
726,43
b7+
803,29
b8+-H2O
914,33
y9+
894,52
y17 2+
890,97
y19 2+
1063,01
b9+
1060,43
b8+
932,34
y10+
1025,56
y18 2+
1012,47
y20 2+
1113,53
y11+
1124,63
b12+-H2O
1343,56
b11+-H2O
1226,50
b12+-NH3
1326,59
y12+
1211,66
y13+
1308,72
b11+
1244,54
b12+
1343,58
b14+-NH3
1528,66
b13+
1474,63
b14+
1545,66
j
Relative Abundance
m/z
R K V R E I pY D K H K S N V G G L L
b4+
b3+
b2+
y+
y3+
b16 3+
650,00
650,34
y1+
132,10
y16 3+-NH3-P, b15 3+-P
598,30
b8 2+
570,78
b9 2+
634,82
y17 3+
679,36
[M+4H]4+-H2O
544,29
[M+4H]4+
548,81
b14 3+
611,99
b13 3+
578,96
b15 3+
630,99
678,37
b17 3+
687,70
b16 4+
487,75
b10 2+-H2O
694,36
b10 2+-NH3
694,86
b10 2+
703,36
616,98
b2 2+
143,12
y2+
245,19
202,08
171,11
265,41
b5 2+
335,21
b6 2+
391,75
480,76
452,49
529,61
b14 2+-P
824,43
784,43
868,47
b16 2+
974,51
1078,44
1136,54
1281,51
1375,10

k

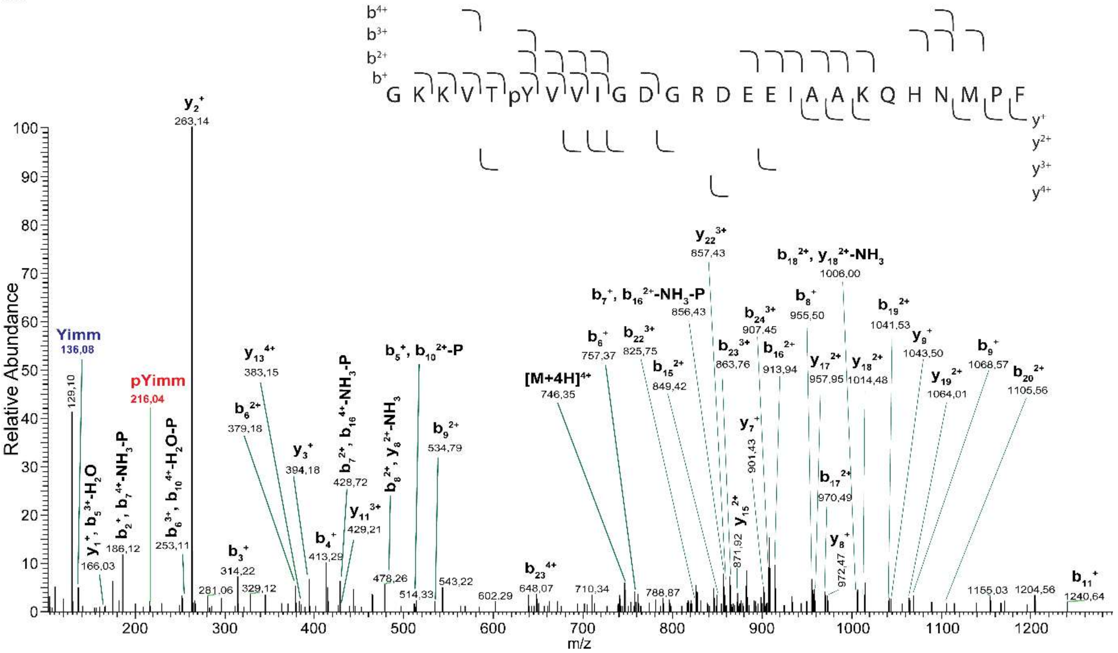

Figure S10. Tyrosine phosphorylated peptides of 6xHis-EYA3 D311N, detected by nLC-MS/MS analysis. Examples of product ion spectra corresponding to phosphopeptides used in the relative quantification analysis, to establish the evolution (5 min to 6 h) of phosphorylation for each of the detected Src-phosphorylated tyrosine residues.

ΔM represents the mass accuracy calculated in the search (made with Proteome Discoverer v1.4).

(**a**) Product ion spectrum of a tyrosine-phosphorylated peptide containing Y67 of 6xHis-EYA3 D311N as phosphorylation site:

IPRSSND**pY**TSQMY, having charge 2+, at m/z 821.33740 Da (ΔM is +3.01 mmu/+3.67 ppm; MH+: 1641.66753 Da). The peptide shown here was identified in the sample from 6 h time point. HCD fragmentation allowed for the identification of the characteristic immonium ion of phosphotyrosine (noted here - pYimm, at m/z 216.04). The immonium ion of tyrosine (Yimm, m/z 136.08) is also present here, but it does not prove the phosphorylation of a tyrosine residue.

(**b**) Product ion spectrum of a tyrosine-phosphorylated peptide containing Y72 of 6xHis-EYA3 D311N as phosphorylation site: TSQM**pY**SAKPY, having charge 2+, at m/z: 628.25903 Da (ΔM is +2.15 mmu/+3.42 ppm; MH+: 1255.51079 Da). The peptide shown here was identified in the sample from 6 h time point. Notice the presence of an ion at m/z of 216.04, identified by Proteome Discoverer as ion b_4^{2+} having a neutral loss of NH_3, but which can also be the pYimm ion.

(**c**) Product ion spectrum of a tyrosine-phosphorylated peptide containing Y77 of 6xHis-EYA3 D311N as phosphorylation site:

SAKP**pY**AHIL, having charge 2+, at m/z: 540.26923 Da (ΔM is +1.31 mmu/+2.43 ppm; MH+: 1079.53118 Da). The peptide shown here was identified in the sample from 5 min time point.

(**d**) Product ion spectrum of a tyrosine-phosphorylated peptide containing Y90 of 6xHis-EYA3 D311N as phosphorylation site:

SVPVSETA**pY**PGQTQY, having charge 2+, at m/z: 853.87262 Da (ΔM is +3.5 mmu/+4.1 ppm; MH+: 1706.73796 Da). The peptide shown here was identified in the sample from 30 min time point. Notice the presence of the pYimm ion at m/z of 216.04.

(**e**) Product ion spectrum of a tyrosine-phosphorylated peptide containing Y96 of 6xHis-EYA3 D311N as phosphorylation site:

AHILSVPVSETAYPGQTQ**pY**QTL, having charge 2+, at m/z: 1242.09924 Da (ΔM is +2.87 mmu/+2.31 ppm; MH+: 2483.19121 Da). The peptide shown here was identified in the sample from 6 h time point. Notice the presence of the pYimm ion at m/z of 216.04.

(**f**) Product ion spectrum of a tyrosine-phosphorylated peptide containing Y105 of 6xHis-EYA3 D311N as phosphorylation site: QTLQQTQP**pY**AVYPQATQTY, having charge 2+, at m/z: 1155.02869 Da (ΔM is +0.75 mmu/+0.65 ppm; MH+: 2309.05010 Da). The peptide shown here was identified in the sample from 2 h time point.

(**g**) Product ion spectrum of a tyrosine-phosphorylated peptide containing Y108 of 6xHis-EYA3 D311N as phosphorylation site:

AV**pY**PQATQTY, having charge 2+, at m/z: 611.26489 Da (ΔM is +1.87 mmu/+3.07 ppm; MH+: 1221.52251 Da). The peptide shown here was identified in the sample from 2 h time point. Notice the presence of the pYimm ion at m/z of 216.04.

(**h**) Product ion spectrum of a tyrosine-phosphorylated peptide containing Y208 of 6xHis-EYA3 D311N as phosphorylation site:

GQNQ**pY**QA**c**YPSSSF, monophosphorylated (Tyr5) and carbamidomethylated (Cys8), having charge 2+, at m/z: 858.82343 Da (ΔM is +1.78 mmu/+2.08 ppm; MH+: 1716.63957 Da). The peptide shown here was identified in the sample from 2 h time point. Notice the presence of the pYimm ion at m/z of 216.04.

(**i**) Product ion spectrum of a tyrosine-phosphorylated peptide containing Y237 of 6xHis-EYA3 D311N as phosphorylation site:

AATT**pY**QSEKPSVMAPAPAAQRL, having charge 3+, at m/z: 790.05176 Da (ΔM is +1.31 mmu/ +1.66 ppm; MH+: 2368.14072 Da). The peptide shown here was identified in the sample from 5 min time point. Notice the presence of the pYimm ion at m/z of 216.04.

(**j**) Product ion spectrum of a tyrosine-phosphorylated peptide containing Y426 of 6xHis-EYA3 D311N as phosphorylation site:

RKVREI**pY**DKHKSNVGGLL, having charge 4+, at m/z: 548.79968 Da (+1.71 mmu/+3.12 ppm; MH+: 2192.17690 Da. The peptide shown here was identified in the sample from 6 h time point.

(**k**) Product ion spectrum of a tyrosine-phosphorylated peptide containing Y532 of 6xHis-EYA3 D311N as phosphorylation site:

GKKVT**pY**VVIGDGRDEEIAAKQHNMPF, having charge 4+, at m/z: 746.36871 Da (ΔM is -0.36 mmu/-0.48 ppm; MH+: 2982.45302 Da). The peptide shown here was identified in the sample from 2 h time point. Notice the presence of the pYimm ion at m/z of 216.04.

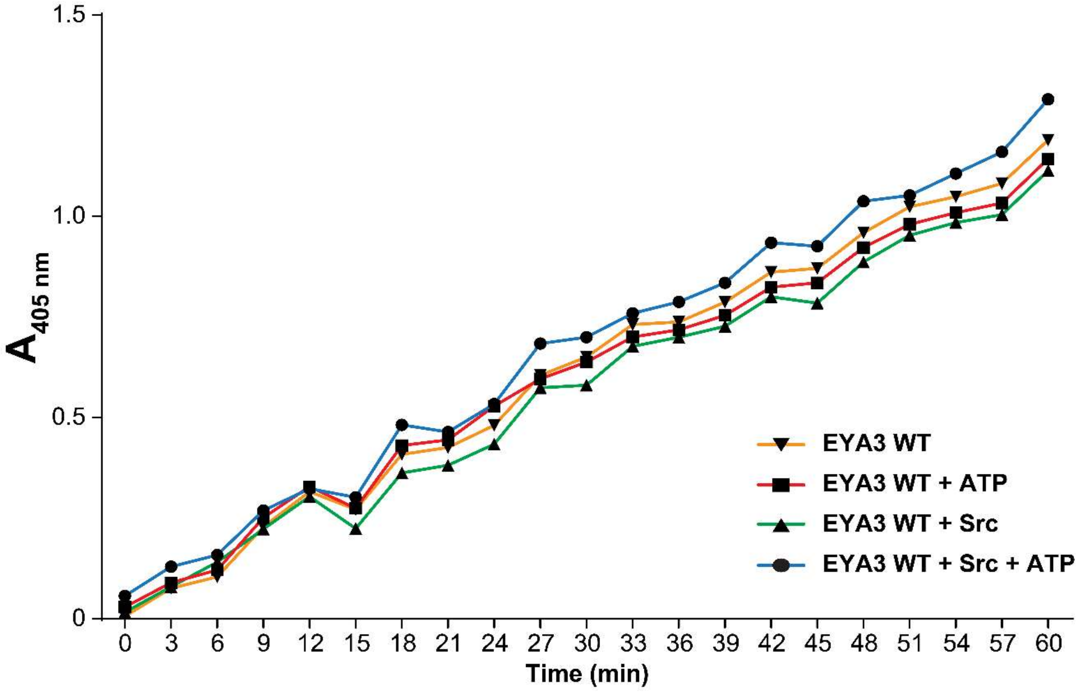

Figure S11. Influence of EYA3 phosphorylation on its activity. Progress curve of EYA3 in the presence and absence of Src phosphorylation. The bacterially expressed and purified 6xHis-EYA3 WT was incubated either alone, with ATP, with Src kinase or with ATP and Src, for 2 h at 37 °C. Next, the activity was assessed on pNPP and the product formed was measured at 405 nm for 1 h and 37 °C, using a spectrophotometer. At every time point, results show the mean A_{450nm} obtained from three technical replicates.

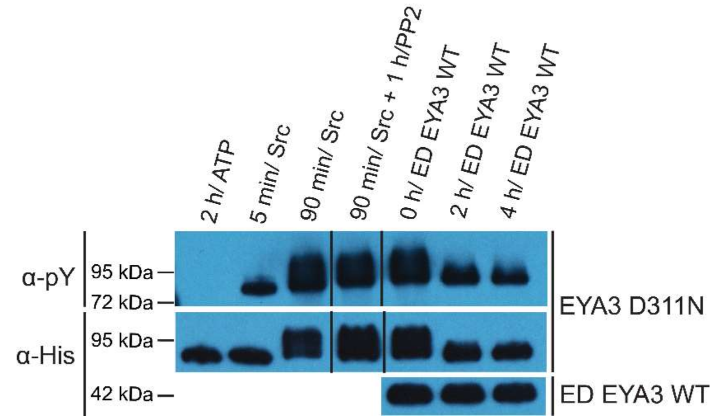

Figure S12. Western blot evidence of ED EYA3 WT dephosphorylation of the Src-phosphorylated EYA3 D311N. Verification of the experiment made in order to establish the dynamics of the in vitro EYA3 D311N tyrosine phosphorylation and dephosphorylation. Samples were verified by Western blot using anti-pTyr and anti-His antibodies. Equal amounts of 6xHis-EYA3 D311N were collected from the starting reaction, at each time point. Broader bands in lanes 3-5 are due to the different electrophoretic mobilities of the multiply phosphorylated molecules of 6xHis-EYA3 D311N. The images are spliced and include dividing lines (vertical black lines) at the splice junction.

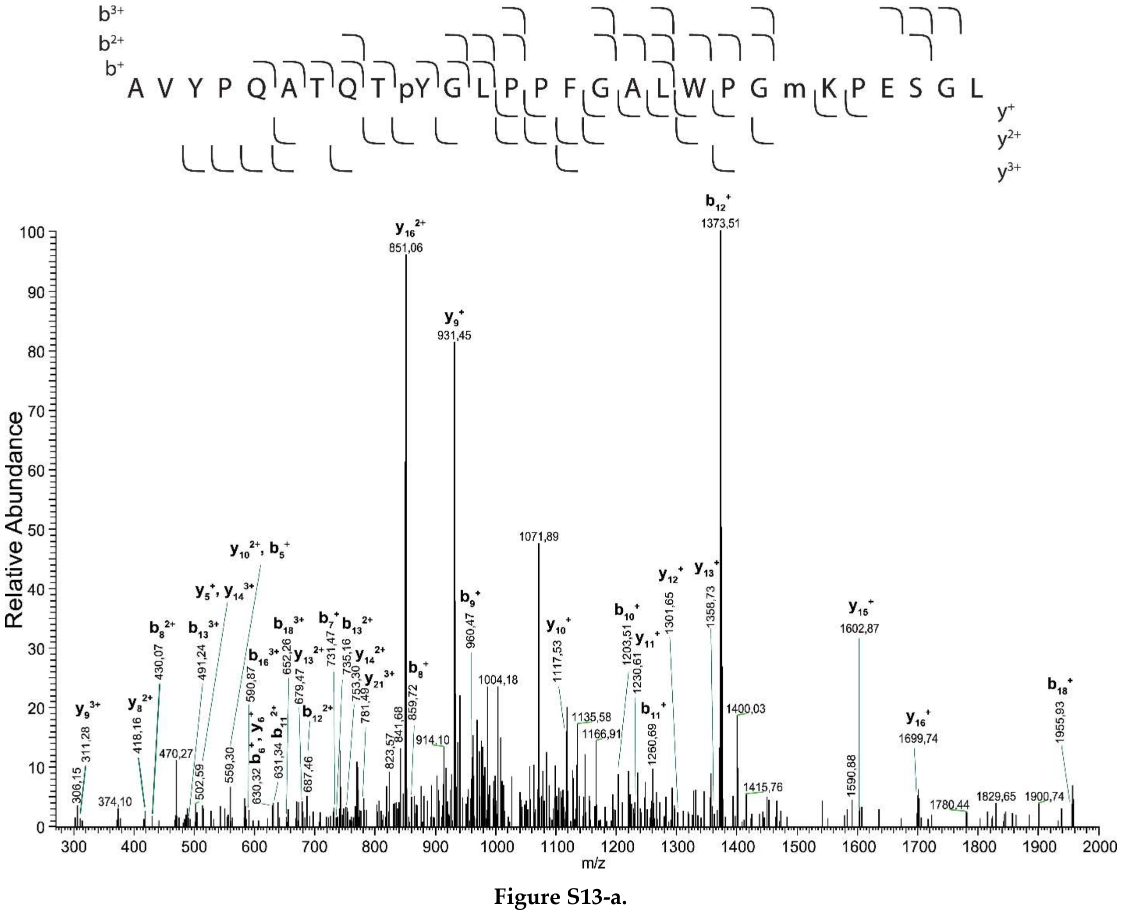

Figure S13-a.

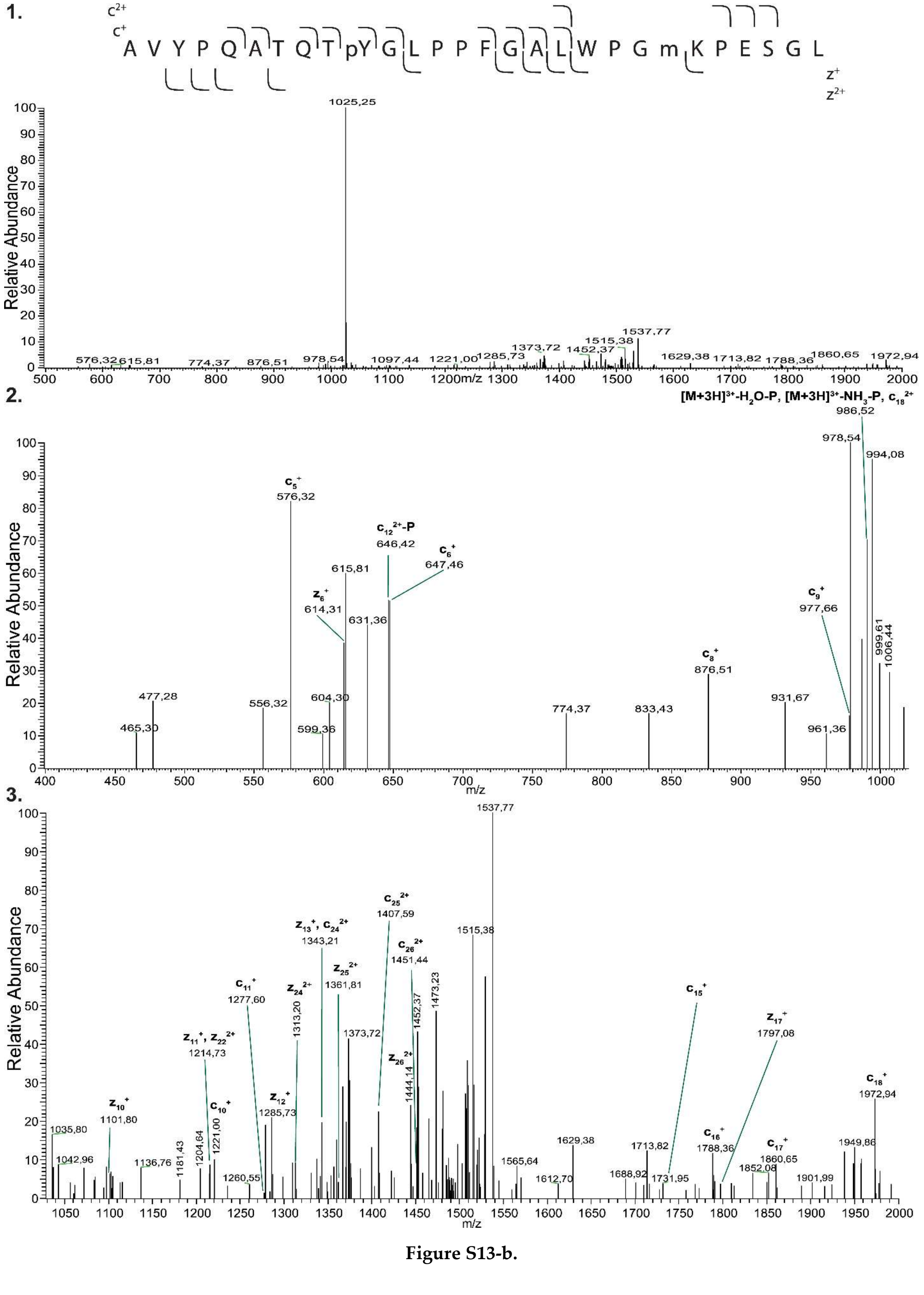
1.
A V Y P Q A T Q T pY G L P P F G A L W P G m K P E S G L
Relative Abundance
m/z
2.
[M+3H]3+-H2O-P, [M+3H]3+-NH3-P, c18 2+
Relative Abundance
m/z
3.
Relative Abundance
m/z

Figure S13-b.

1.

2.

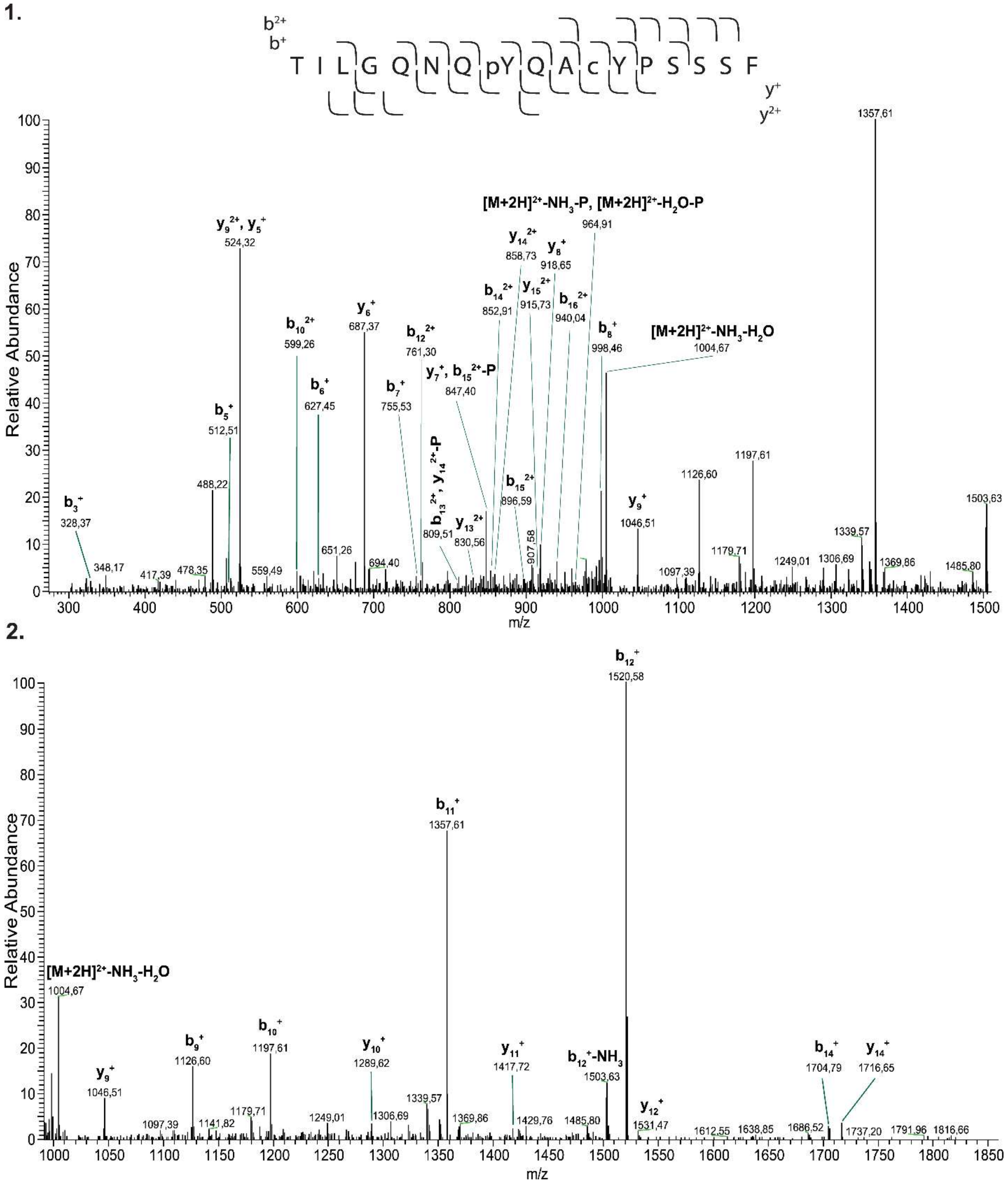

Figure S13-c.

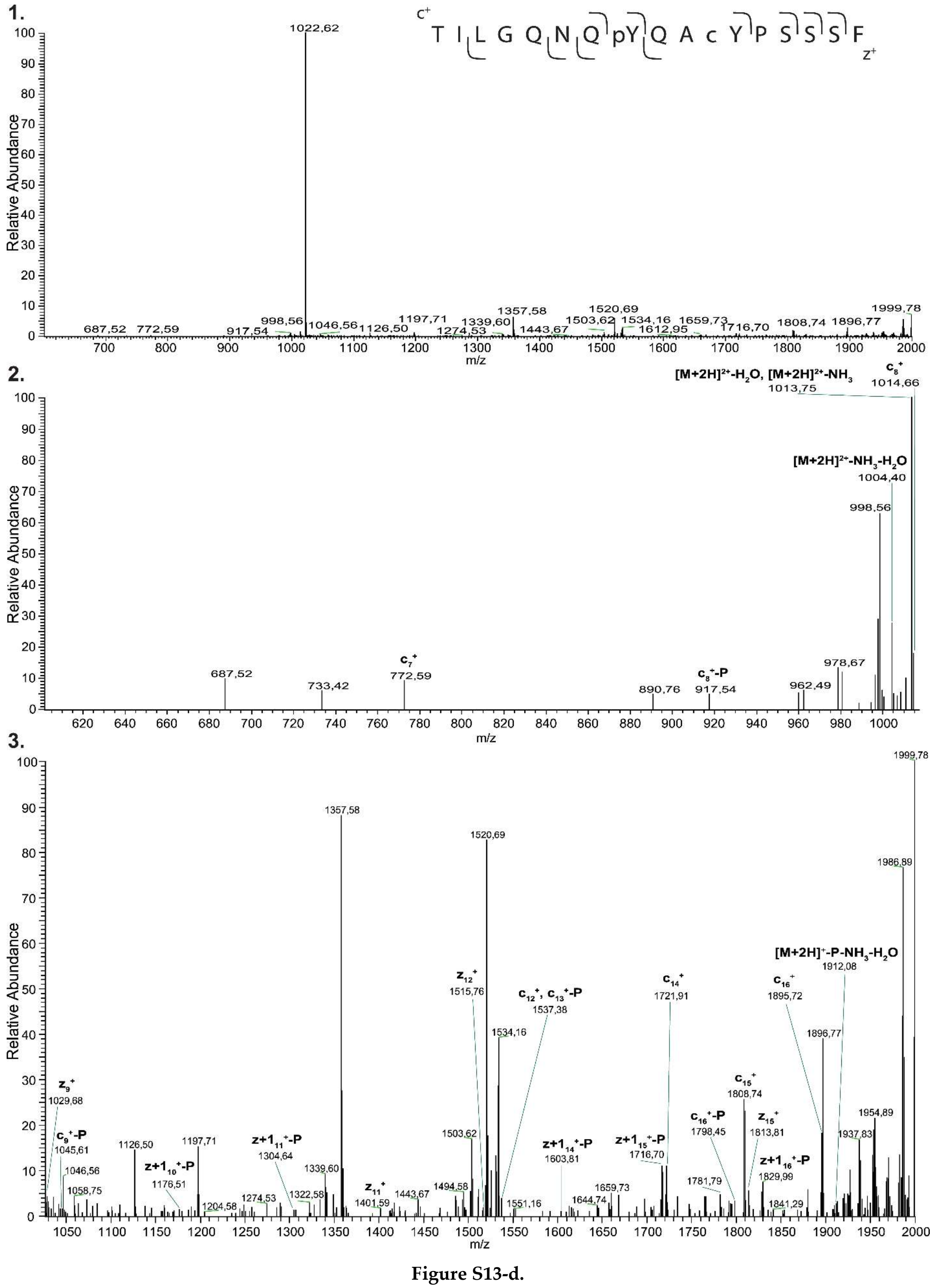

1.
2.
3.
c+ T I L G Q N Q pY Q A c Y P S S S F z+
Relative Abundance
m/z
1022,62
687,52
772,59
917,54
998,56
1046,56
1126,50
1197,71
1274,53
1339,60
1357,58
1443,67
1503,62
1520,69
1534,16
1612,95
1659,73
1716,70
1808,74
1896,77
1999,78
[M+2H]2+-H2O, [M+2H]2+-NH3
1013,75
c8+
1014,66
[M+2H]2+-NH3-H2O
1004,40
998,56
978,67
962,49
c8+-P
917,54
890,76
c7+
772,59
733,42
687,52
1999,78
1357,58
1520,69
1986,89
[M+2H]+-P-NH3-H2O
1912,08
z12+
1515,76
c12+, c13+-P
1537,38
c14+
1721,91
c16+
1895,72
1534,16
1896,77
z9+
1029,68
c15+
1808,74
c9+-P
1045,61
c16+-P
1798,45
z15+
1813,81
1954,89
1503,62
1937,83
z+115+-P
1716,70
z+114+-P
1603,81
z+116+-P
1829,99
1126,50
1197,71
z+111+-P
1304,64
1046,56
z+110+-P
1176,51
1339,60
z11+
1401,59
1443,67
1494,58
1781,79
1058,75
1322,58
1659,73
1274,53
1644,74
1204,58
1551,16
1841,29

Figure S13-d.

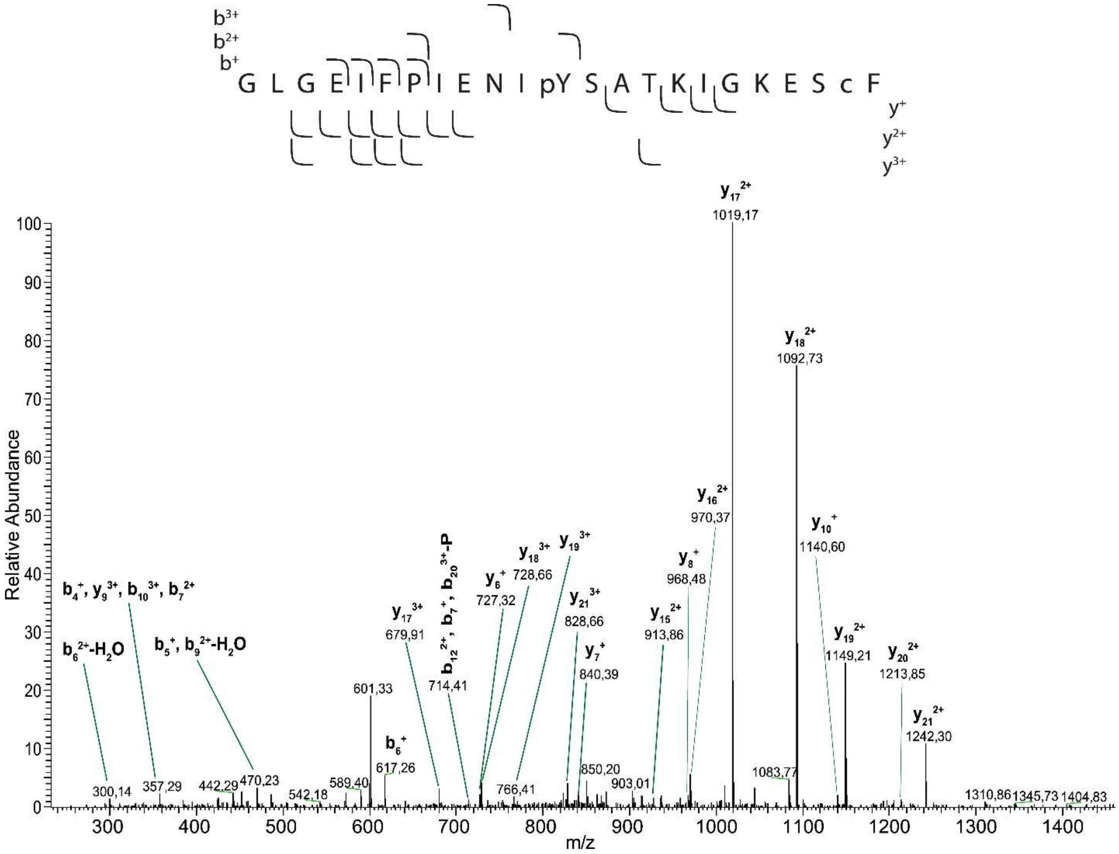

b3+
b2+
b+
G L G E I F P I E N I pY S A T K I G K E S c F
y+
y2+
y3+
Relative Abundance
m/z
y17 2+ 1019,17
y18 2+ 1092,73
y16 2+ 970,37
y10 + 1140,60
y8 + 968,48
y19 2+ 1149,21
y20 2+ 1213,85
y21 2+ 1242,30
y15 2+ 913,86
y21 3+ 828,66
y7 + 840,39
y18 3+ 728,66
y19 3+
y6 + 727,32
y17 3+ 679,91
b12 2+, b7 +, b20 3+-P 714,41
601,33
b6 + 617,26
b4 +, y9 3+, b10 3+, b7 2+
b6 2+-H2O
b5 +, b9 2+-H2O
300,14
357,29
442,29
470,23
542,18
589,40
766,41
850,20
903,01
1083,77
1310,86
1345,73
1404,83

Figure S13-e.

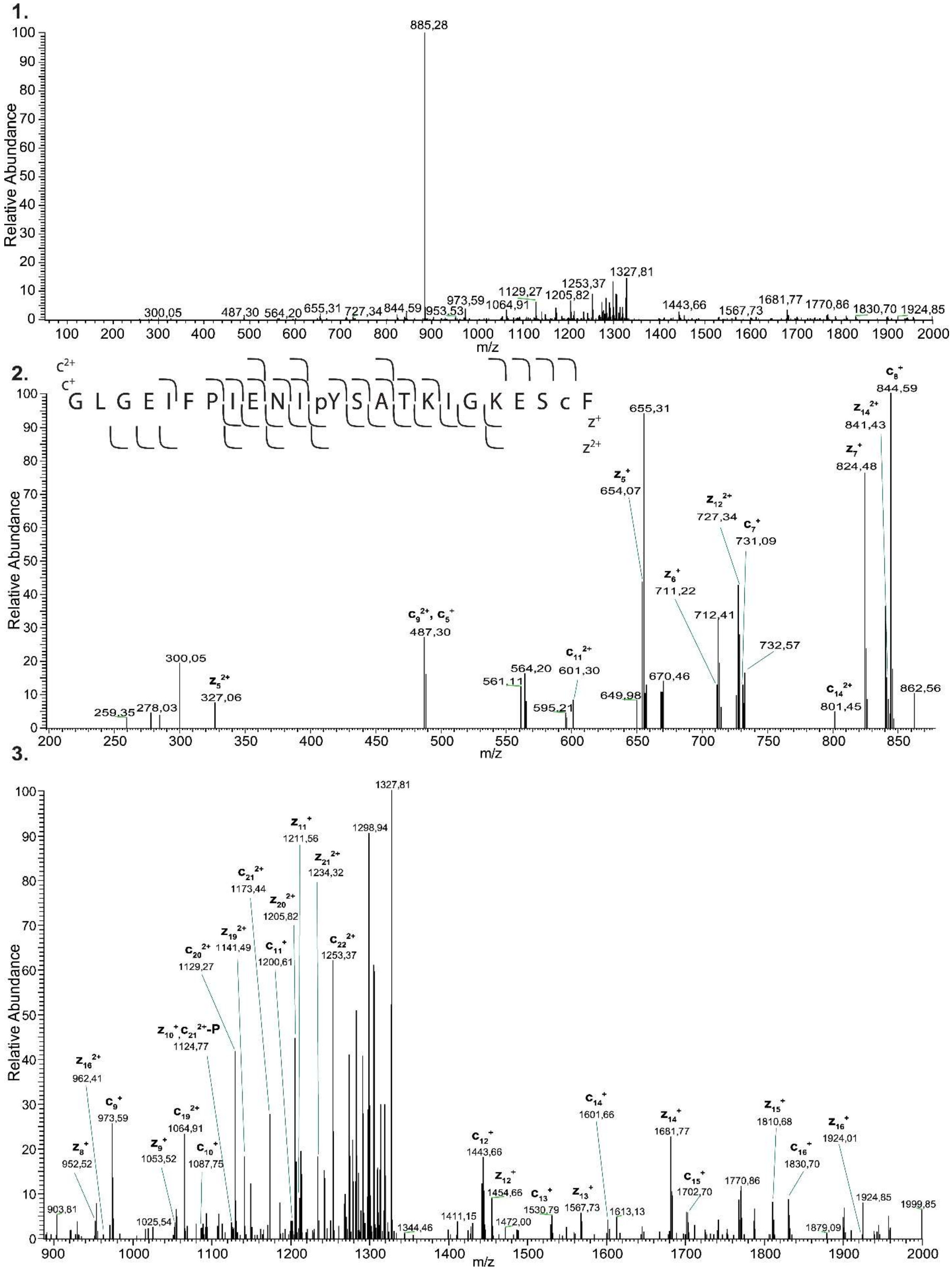

Figure S13-f.

Figure S13. Tyrosine phosphorylated peptides of 6xHis-EYA3 D311N, detected by nLC-MS/MS analysis. These are examples of product ion spectra corresponding to phosphopeptides which were used in the relative quantification analysis, to establish the evolution of phosphorylation for Y115, Y208 and Y508 (see Figure 4). Compared to the time-course in vitro phosphorylation of EYA3 D311N (Figure 3b), a different phosphopeptide representing evolution of phospho-Y208 was used. Phospho-Y115 and phospho-Y508 have not been detected in the time-course in vitro phosphorylation reaction of EYA3.

ΔM represents the mass accuracy calculated in the search (made with Proteome Discoverer v1.4).

(**a**) Product ion spectrum (obtained through CID fragmentation) of a tyrosine-phosphorylated peptide containing Y115 of 6xHis-EYA3 D311N as phosphorylation site:

AVYPQATQT**pY**GLPPFGALWPG**m**KPESGL, monophosphorylated (Tyr10) and oxidized (Met22), having charge 3+, at m/z: 1024.82227 Da (ΔM is -1.86 mmu/-1.81 ppm; MH+: 3072.45224 Da). The peptide shown here was identified in the sample from 0 h/ ED EYA3 WT time point. Not to complicate the spectrum, all 1+ charged, but not all 2+ and 3+ charged b and y ions, were indicated.

(**b**) Product ion spectrum (obtained through ETD fragmentation) of a tyrosine-phosphorylated peptide containing Y115 of 6xHis-EYA3 D311N as phosphorylation site:

AVYPQATQT**pY**GLPPFGALWPG**m**KPESGL, monophosphorylated (Tyr10) and oxidized (Met22), having charge 3+, at m/z: 1024.82227 Da (ΔM is -1.86 mmu/-1.81 ppm; MH+: 3072.45224 Da). The peptide shown here was identified in the sample from 0 h/ ED EYA3 WT time point.

1. Original product ion spectrum obtained through ETD fragmentation.
2. Zoom in on the 400 - 1020 m/z range.
3. Zoom in on the 1030 - 2000 m/z range.

(**c**) Product ion spectrum (obtained through CID fragmentation) of a tyrosine-phosphorylated peptide containing Y208 of 6xHis-EYA3 D311N as phosphorylation site:

TILGQNQ**pY**QA**c**YPSSSF, monophosphorylated (Tyr8) and carbamidomethylated (Cys11), having charge 2+, at m/z: 1022.42944 Da (ΔM is -0.11 mmu/-0.11 ppm; MH+: 2043.85161 Da). The peptide shown here was identified in the sample from 90 min/ Src + 1 h/ PP2 time point.

1. Zoom in on the 300 - 1500 m/z range.
2. Zoom in on the 1000 - 1850 m/z range.

(**d**) Product ion spectrum (obtained through ETD fragmentation) of a tyrosine-phosphorylated peptide containing Y208 of 6xHis-EYA3 D311N as phosphorylation site:

TILGQNQ**pY**QA**c**YPSSSF, monophosphorylated (Tyr8) and carbamidomethylated (Cys11), having charge: +2, at m/z: 1022.42944 Da (ΔM is -0.11 mmu/-0.11 ppm; MH+: 2043.85161 Da). The peptide shown here was identified in the sample from 90 min/ Src + 1 h/ PP2 time point.

1. Original product ion spectrum obtained through ETD fragmentation.
2. Zoom in on the 600 - 1015 m/z range.
3. Zoom in on 1030 - 2000 m/z range.

(**e**) Product ion spectrum (obtained through CID fragmentation) of a tyrosine-phosphorylated peptide containing Y508 of 6xHis-EYA3 D311N as phosphorylation site:

GLGEIFPIENI**pY**SATKIGKES**c**F, monophosphorylated (Tyr12) and carbamidomethylated (Cys22), having charge 3+, at m/z: 885.09125 Da (ΔM is -0.95 mmu/-1.08 ppm; MH+: 2653.25919 Da). The peptide shown here was identified in the sample from 0 h/ ED EYA3 WT time point.

(**f**) Product ion spectrum (obtained through ETD fragmentation) of a tyrosine-phosphorylated peptide containing Y508 of 6xHis-EYA3 D311N as phosphorylation site:

GLGEIFPIENI**pY**SATKIGKES**c**F, monophosphorylated (Tyr12) and carbamidomethylated (Cys22), having charge 3+, at m/z: 885.09125 Da (ΔM is -0.95 mmu/-1.08 ppm; MH+: 2653.25919 Da). The peptide shown here was identified in the sample from 0 h/ ED EYA3 WT time point.

1. Original product ion spectrum obtained through ETD fragmentation.
2. Zoom in on the 200 - 870 m/z range.
3. Zoom in on the 900 - 2000 m/z range.

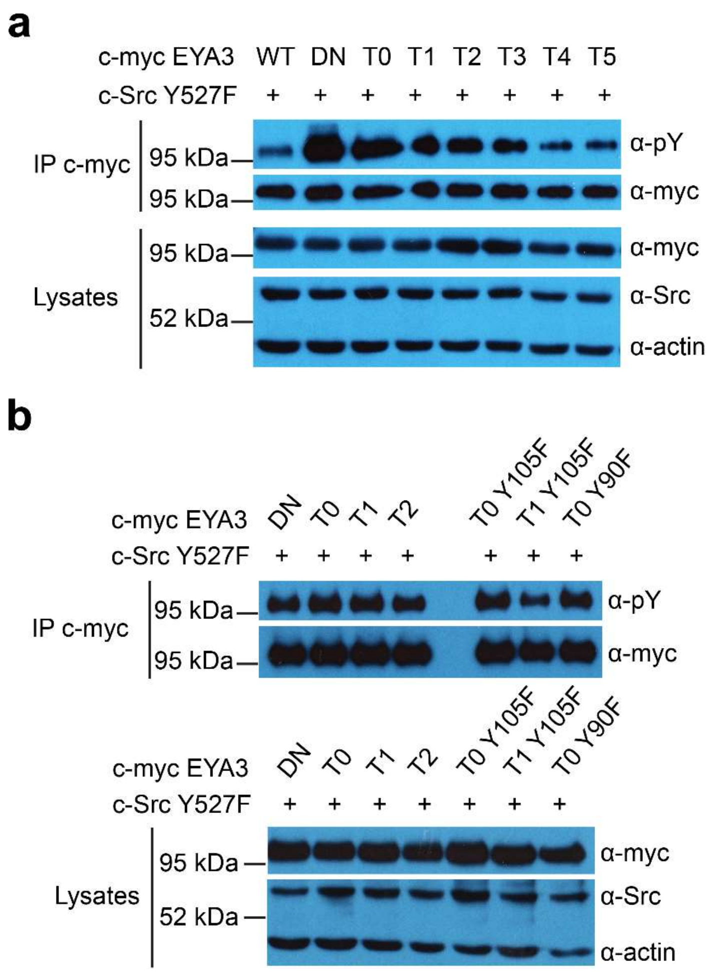

Figure S14. Representative Western blot results of experiments made in order to evaluate tyrosine phosphorylation of various EYA3 constructs. HEK293T cell lysates transiently overexpressing c-myc tagged EYA3 proteins and c-Src Y527F were verified by Western blot using anti-c-myc, anti-v-Src and anti-actin antibodies. Tyrosine phosphorylation of immunoprecipitated myc-tagged EYA3 proteins was evaluated using anti-pY and anti-c-myc antibodies. Representative Western blot results are shown in (**a**) and (**b**).

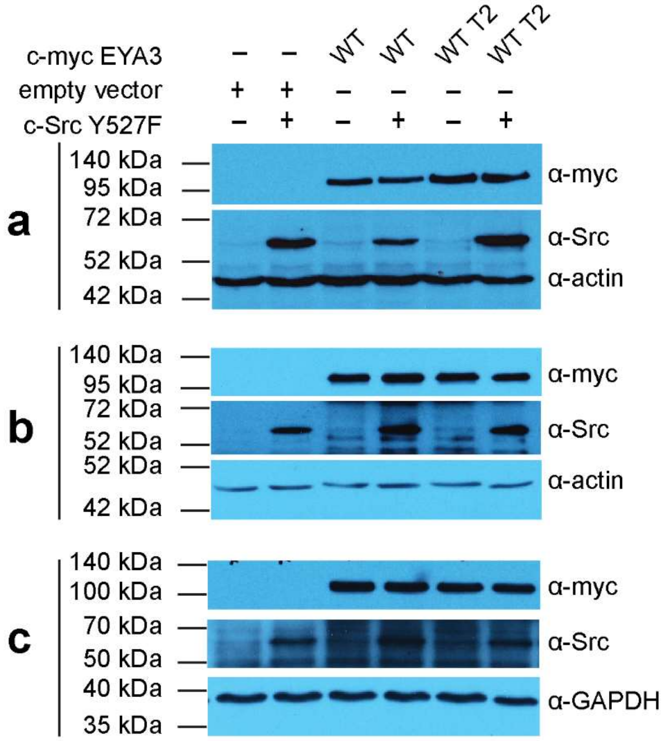

Figure S15. Western blot analysis of samples subjected to MTS assay.

Western blot analyses were made to verify the expression of c-myc-tagged EYA3 proteins and c-Src Y527F in HEK293T cells. GAPDH or actin were used as loading controls. Samples were taken 24 h post-transfection from the cells which were later subjected to MTS assay. (**a**), (**b**) and (**c**) All three independent experiments are shown.

a

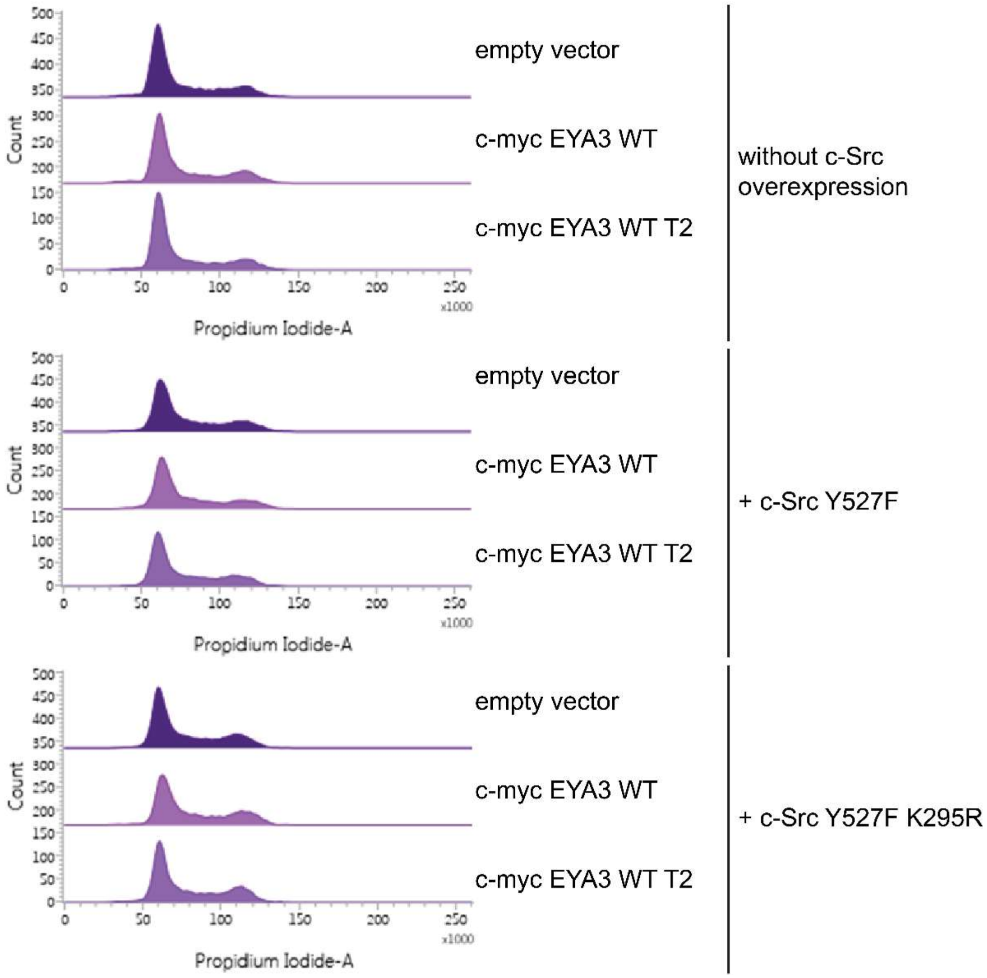

empty vector
c-myc EYA3 WT
c-myc EYA3 WT T2
without c-Src overexpression
+ c-Src Y527F
+ c-Src Y527F K295R
Count
Propidium Iodide-A
x1000

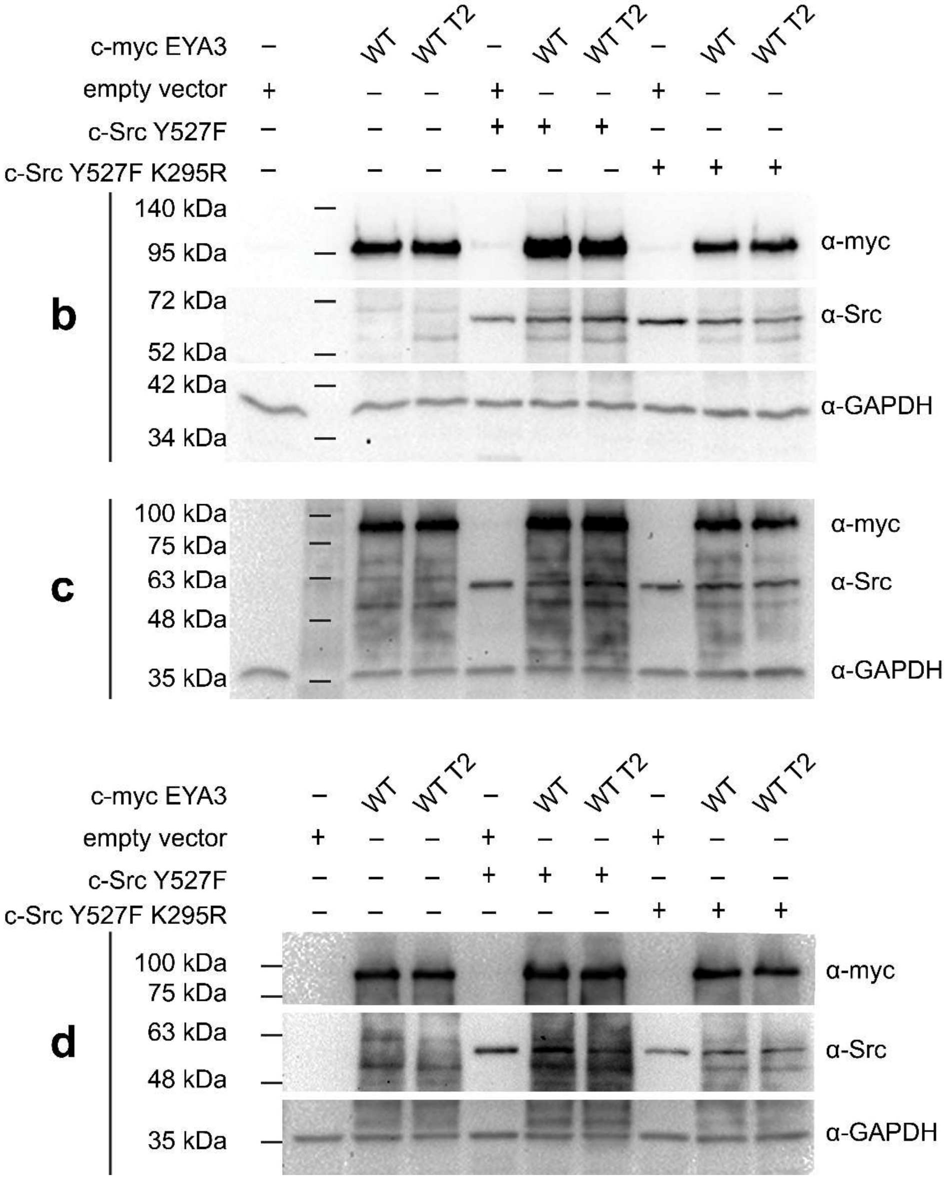

Figure S16. Cell cycle analysis by flow cytometry. (**a**) Raw data obtained from flow cytometry analysis of cells stained with propidium iodide, 24 h post-transfection (representative experiment out of the three performed). (**b**), (**c**) and (**d**) Western blot results from samples subjected to cell cycle analysis by flow cytometry. Twenty-four hours post-transfection, a fraction of the cells was prepared for cell cycle analysis by flow cytometry and the rest were lysed and subjected to Western blot analysis to verify the expression of c-myc-tagged EYA3 proteins, c-Src Y527F, c-Src Y527F K295R and the loading control, GAPDH. Three experiments are shown.

a

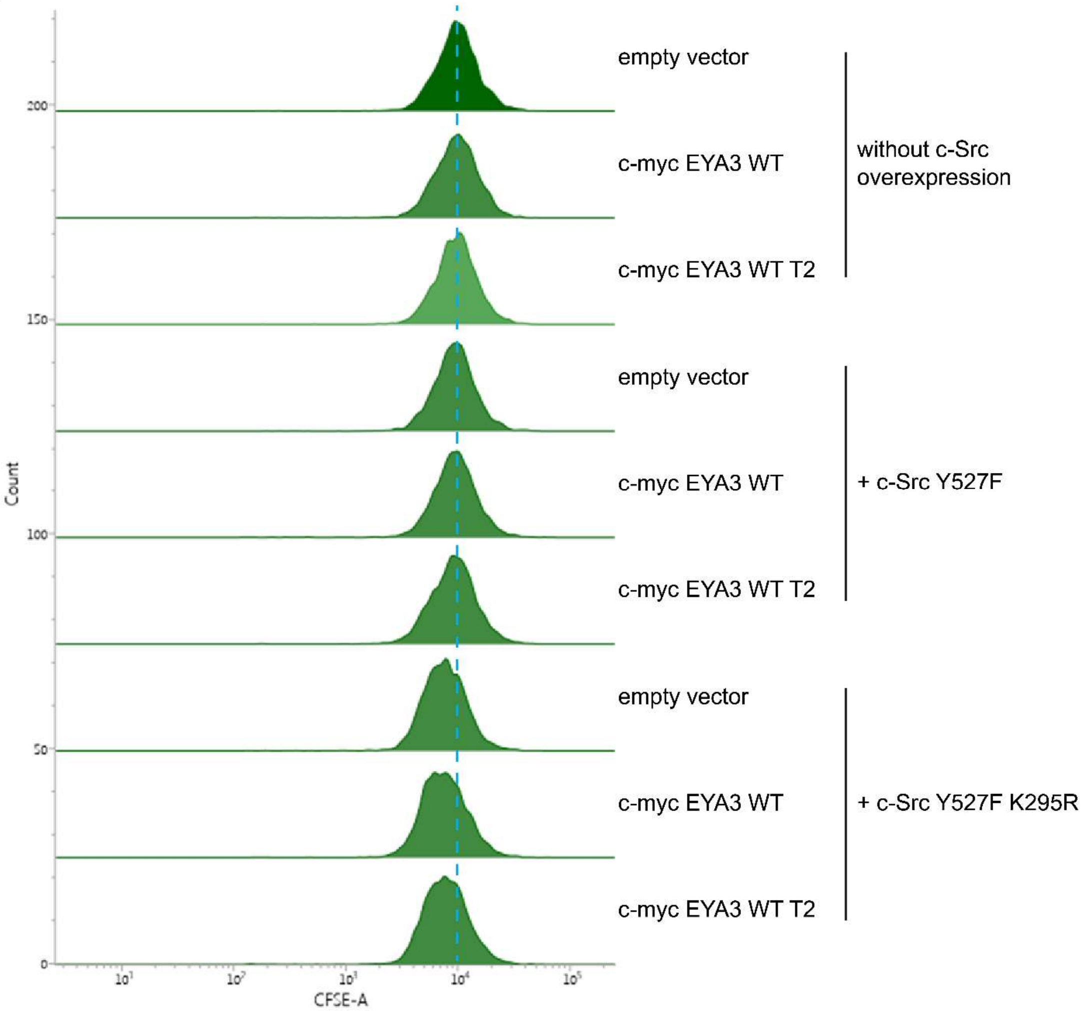
empty vector
c-myc EYA3 WT
c-myc EYA3 WT T2
without c-Src overexpression
empty vector
c-myc EYA3 WT
c-myc EYA3 WT T2
+ c-Src Y527F
empty vector
c-myc EYA3 WT
c-myc EYA3 WT T2
+ c-Src Y527F K295R
Count
200
150
100
50
0
CFSE-A

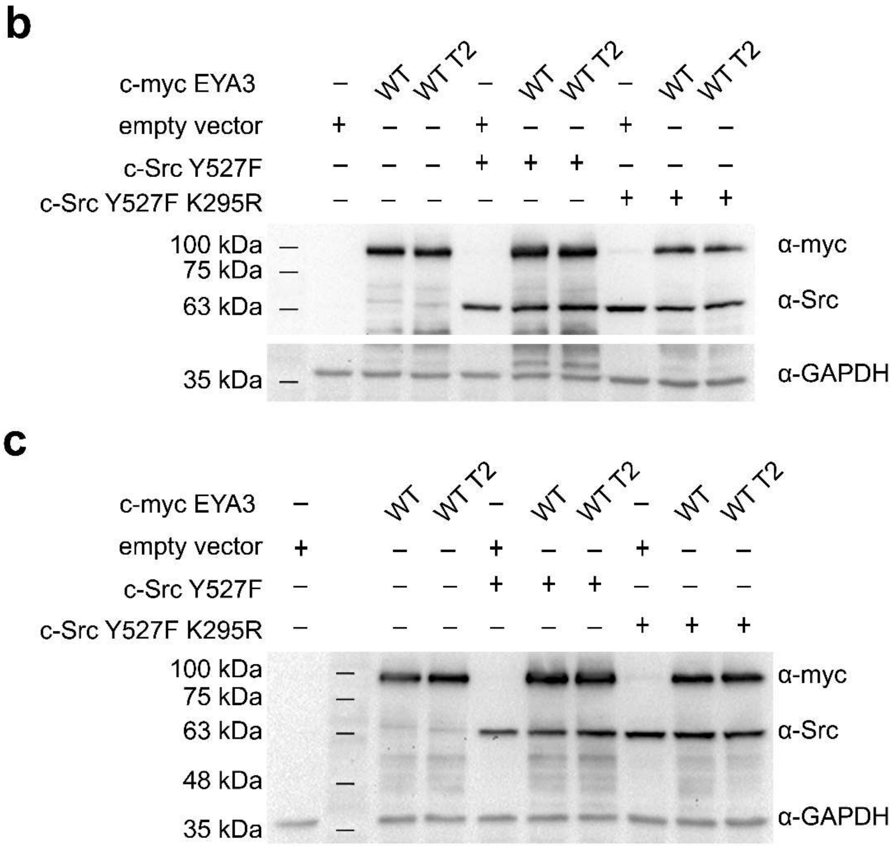

Figure S17. Flow cytometry analysis of CFSE stained cells. (**a**) Raw data obtained from flow cytometry analysis (48 h post-transfection) of cells stained with CFSE (representative experiment out of two performed). The blue dashed line evidences the peak of the CFSE-A distribution corresponding to the control sample (empty vector). (**b**) and (**c**) Western blot results from samples subjected to flow cytometry analysis after CFSE staining. Forty-eight hours post-transfection, after flow cytometry analysis, the remaining HEK293T cells were lysed and subjected to Western blot analysis to verify the expression of c-myc-tagged EYA3 proteins, c-Src Y527F, c-Src Y527F K295R and the loading control, GAPDH.

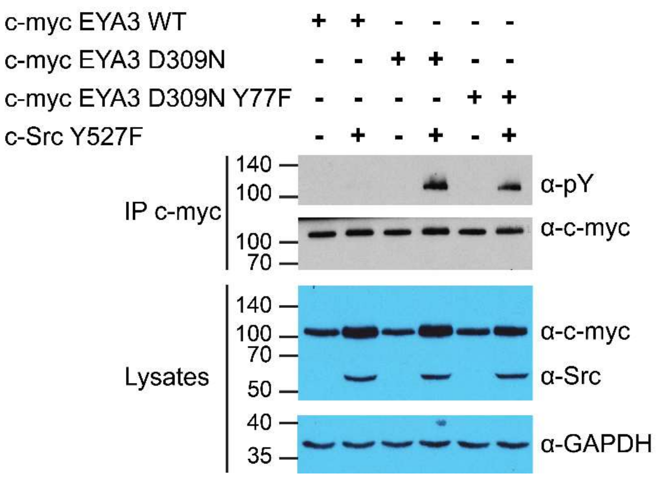

Figure S18. Representative Western blot analysis for detection of phosphorylated residues of transiently expressed EYA3. In order to identify the Src-induced phosphorylation sites of EYA3 overexpressed in HEK293T cells, after transient transfection followed by cell harvesting and lysis,

protein expression was verified by Western blot. First, Western blot analyses were made on the lysates to verify the expression of c-myc-tagged EYA3 proteins and c-Src Y527F. GAPDH was used as loading control. After IP of c-myc tagged EYA3 proteins was performed using anti-c-Myc (9E10, sc-40) antibody, the samples were again subjected to verification by Western blot. Anti-pY and anti-c-myc antibodies were used. This figure is representative for the two independent experiments made.

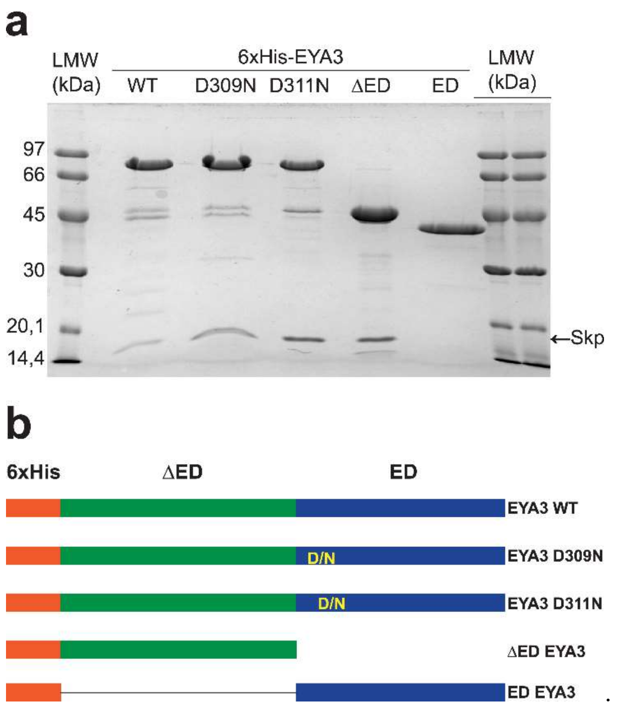

Figure S19. Skp is present in the prokaryotically expressed and purified EYA3 protein samples. (**a**) Coomassie stained SDS-PAGE gel with different purified N-terminal His-tagged EYA3 proteins: EYA3 WT, EYA3 D309N, EYA3 D311N, ΔED EYA3 WT and ED EYA3 WT (all purified after prokaryotic expression). Notice the presence of Skp chaperone in all the samples except ED EYA3 WT. LMW represents the protein ladder (Low Molecular Weight Marker). (**b**) Schematic representation of the purified N-terminal His-tagged EYA3 proteins.

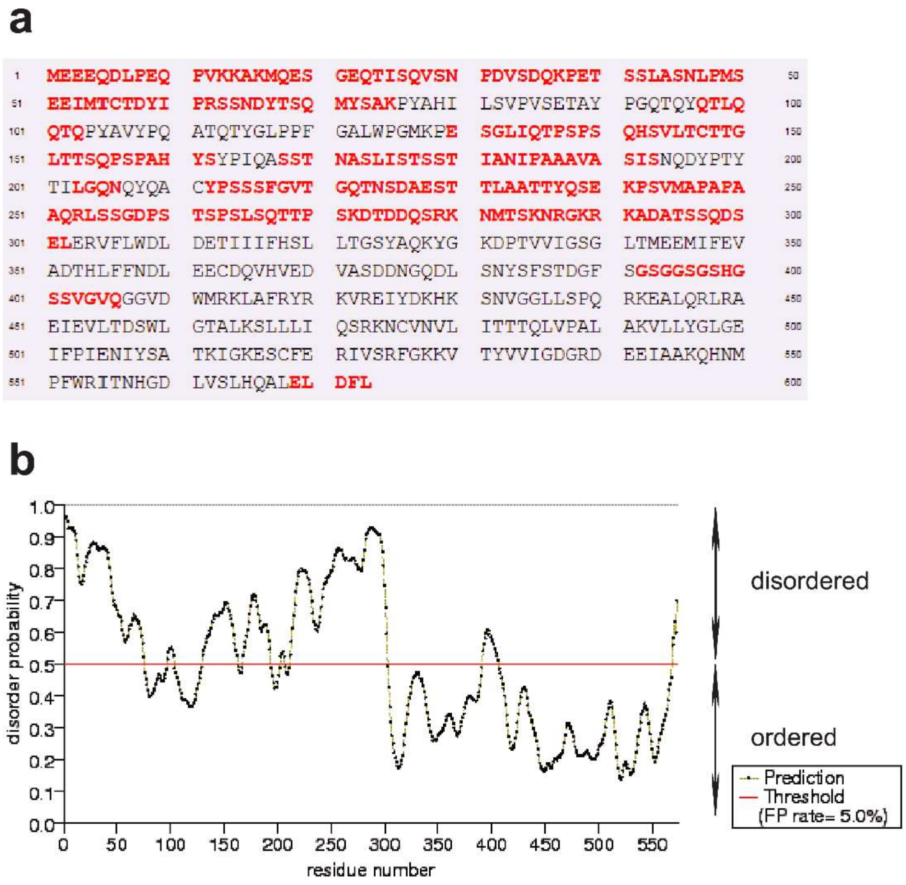

Figure S20. Disorder profile plot of EYA3 WT. (**a**) EYA3 WT disorder prediction using PrDos at a 5% false positive level, mapped on the protein sequence (red/ black - above/ bellow disorder threshold). (**b**) Correspondent disorder profile plot.

Table S1. EYA3 D309N Y → F mutants.

Table containing the successive Y → F mutants made starting from c-myc-EYA3 D309N and used to evaluate the contribution of identified phosphotyrosine residues to the overall EYA3 phosphorylation.

EYA3 D309N Y→F mutants obtained by site-directed mutagenesis	Short name
EYA3 D309N **Y237F**[1]	T0
EYA3 D309N Y237F **Y96F**	T1
EYA3 D309N Y237F Y96F **Y77F**	T2
EYA3 D309N Y237F Y96F Y77F **Y105F**	T3
EYA3 D309N Y237F Y96F Y77F Y105F **Y90F**	T4
EYA3 D309N Y237F Y96F Y77F Y105F Y90F **Y115F**	T5
EYA3 D309N Y237F Y96F Y77F Y105F Y90F Y115F **Y208F**	T6
EYA3 D309N Y237F Y96F Y77F Y105F Y90F Y115F Y208F **Y108F**	T7
EYA3 D309N Y237F Y96F Y77F Y105F Y90F Y115F Y208F **Y67F**	T6 Y67F
EYA3 D309N Y237F Y96F Y77F Y105F Y90F Y115F Y208F Y67F **Y72F**	T8
EYA3 D309N Y237F Y96F Y77F Y105F Y90F Y115F Y208F Y67F Y72F **Y108F**	T9
EYA3 D309N Y237F Y96F Y77F Y105F Y90F Y115F Y208F Y67F Y72F Y108F **Y508F**	T10
EYA3 D309N Y237F Y96F Y77F Y105F Y90F Y115F Y208F Y67F Y72F Y108F Y508F **Y426F**	T11

[1] The last Y → F mutation added is highlighted in bold.
